# Single-atom nanozymes in nanomedicine: catalytic precision, mechanistic insights, and translational opportunities

**DOI:** 10.1039/d6sc03929d

**Published:** 2026-09-17

**Authors:** Jinpei Cui, Xiaomin Xia, Xingyun Li, Dan Luo, Kaiqing Song, Sa Sun, Jie Liu, Shubiao Xia, Mostafa Kamal Masud, Yusuke Yamauchi, Xue Li

**Affiliations:** a Department of Stomatology, The Affiliated Hospital of Qingdao University, Qingdao University Qingdao 266000 China; b School of Stomatology, Qingdao University Qingdao 266003 China lixue@qdu.edu.cn; c Institute of Materials for Energy and Environment, College of Materials Science and Engineering, Qingdao University Qingdao 266071 China xingyun_2008@sina.cn; d Yunnan Key Laboratory of Crystalline Porous Organic Functional Materials, College of Chemical and Materials Engineering, Qujing Normal University China; e Australian Institute for Bioengineering and Nanotechnology (AIBN), The University of Queensland Brisbane QLD 4072 Australia m.masud@uq.edu.au; f Department of Materials Process Engineering, Graduate School of Engineering, Nagoya University 464-8603 Nagoya Japan

## Abstract

Nanozymes, engineered by integrating enzymatic catalytic properties into nanomaterials, hold significant potential for biomedical applications. However, achieving high catalytic efficiency and substrate selectivity remains challenging. Single-atom nanozymes (SANs), featuring well-defined metal–ligand coordination environments and high atomic utilization efficiency, may provide catalytic advantages over conventional nanoparticles in specific reaction systems and under defined structural and operating conditions. While previous reviews have provided comprehensive overviews, a systematic analysis of the structure–activity relationships that govern SANs' performance in biological environments is still lacking. This review fills this gap by focusing on the atomic-level engineering strategies including coordination environment modulation, spatial configuration control, dual-atom sites, and emerging non-metal systems that have advanced the field since 2022. We critically compare the catalytic performance of SANs with that of nanoparticles and nanoclusters, clarifying the scope and limitations of the size–performance relationship in biocatalysis. Biomedical applications including antimicrobial therapy, cancer treatment, biosensing, and oxidative-stress protection are examined through the lens of catalytic mechanisms, with particular attention to how structural features translate into biological outcomes. The translational landscape is critically assessed, identifying both the opportunities and the barriers that must be overcome for clinical implementation. By integrating mechanistic insights with engineering principles, this Review provides a conceptual framework for the rational design of next-generation SANs.

## Abstract 2

Atomic-level engineering of single-atom nanozymes (SANs) links rational design, catalytic mechanisms, and structure–activity relationships to translational opportunities in antibacterial and cancer therapy, biosensing, and cytoprotection.

## Graphical abstract

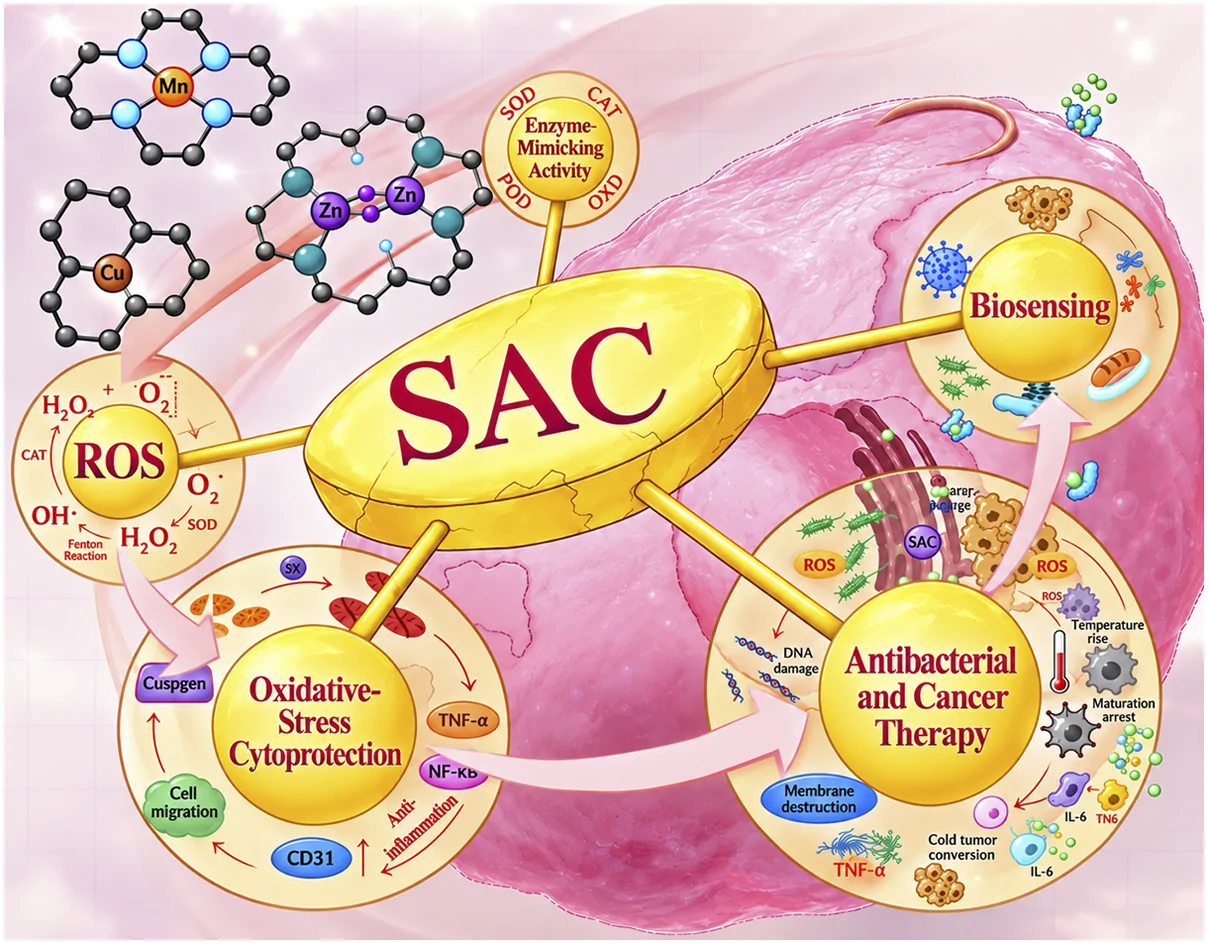

## 1. Introduction

The advancement of nanomaterial research has fostered the development of nanozymes, which are engineered nanomaterials tailored to mimic natural enzymatic activity and have emerged as a pivotal frontier in nanomedicine.^1^ The term “nanozymes” was formally introduced by Manea *et al.* in 2004.^2^ Yan's research team first reported the peroxidase-mimetic activity of Fe_3_O_4_ nanozymes in 2007, a work that firmly consolidated the concept of nanozymes.^3^ Recent studies have centered on the development of metal-based nanozymes with multi-enzyme active sites, such as gold nanoparticles (GNPs),^4^ TiO_2_,^5^ CaO_2_,^6^ Fe_3_O_4_,^7^ and CeO_2_.^8,9^ These materials enable targeted enhancement of catalytic performance through precise modulation of the active site coordination environment, thus exhibiting substantial potential in tumor therapy^10–13^ and anti-infection^14^ applications. However, nanozymes still confront substantial challenges in practical deployment, including limited catalytic efficiency, insufficient substrate selectivity, and unresolved long-term biosafety concerns. To address these bottlenecks, systematic solutions must be developed through the intimate integration of material innovation and mechanistic investigation.

Indeed, intrinsic physicochemical properties and external stimuli significantly influence the catalytic activity of nanozymes. Key factors including size, elemental composition, crystal structure, surface states, and heteroatom doping exert critical regulatory effects on their catalytic behavior. Thus, the rational design of nanozymes with well-defined mechanisms and precise active sites is pivotal for advancing the biomedical applications of nanozyme-triggered catalytic reactions. Notably, the catalytic performance of metallic nanostructures is highly dependent on their dimensional and morphological characteristics, which directly modulate the electronic properties of active sites. Comparative studies of nanoscale and sub-nanoscale metal clusters have revealed that nanoscale counterparts exhibit relatively better catalytic activity and selectivity. This size- and shape-dependent behavior extends across nanoscale architectures, from nanoparticles and nanoclusters to single-atom systems.^15^

To improve the performance of nanocatalysts, Qiao, Zhang and co-workers pioneered the concept of single-atom catalysts (SACs), demonstrating CO oxidation catalyzed by individual Pt atoms anchored on FeO_*x*_ supports (Fig. 2).^2,3,16–21^ SACs exhibit exceptional catalytic activity and selectivity owing to their maximized atomic utilization efficiency and well-defined active sites. In 2019, Zhao *et al.* reported a heterogeneous single-iron-atom catalyst with peroxidase (POD)-like activity, whose catalytic efficiency was 40-fold higher than that of Fe_3_O_4_ nanozymes. This milestone marked the formal entry of single-atom nanozymes (SANs) into the field of biomedical applications.^20^ This innovation laid the foundation for SANs, which have since emerged as transformative platforms in nanomedicine.

Precise modulation of the coordination environment of the metal center and optimization of support properties can further enhance the enzyme-mimetic activity of such systems. SANs synergistically combine the atom-economic advantages with the enzymatic functionality of natural proteins, addressing critical limitations of conventional nanozymes such as low atomic utilization efficiency and ambiguous active site configurations.^19,22^ Studies have shown that SANs exhibit notable POD-like activity, with high substrate specificity, exceptional turnover frequencies, and precise reactive oxygen species (ROS) generation capabilities for targeted tumor ablation and sensitive biosensing applications.^23–29^ Their superoxide dismutase (SOD)-like activity mitigates oxidative stress through efficient free radical scavenging.^30–33^ Moreover, catalase (CAT)-like activity alleviates tumor hypoxia and enhances the efficacy of photodynamic and radiotherapeutic treatments.^34–37^ Furthermore, oxidase (OXD)-like activity enables synergistic applications in antibacterial and anticancer photodynamic therapy (Fig. 1).^38–42^ Additionally, certain redox enzyme-mimetic activities such as glutathione peroxidase (GPx)-like activity catalyze the oxidation of glutathione (GSH). This intracellular GSH depletion enhances tumor cell sensitivity to oxidative therapies.^43–46^ Meanwhile, benefiting from high structural stability and high theoretical atomic utilization efficiency, SANs may reduce the potential for cytotoxic metal leakage compared with conventional nanoparticles, thereby contributing to favorable biosafety profiles under the evaluated conditions.^47^ These attributes position SANs as promising platforms for next-generation nanocatalytic medicine. Notably, the structurally well-defined nature of SANs enables precise identification of catalytic sites, thereby facilitating mechanistic understanding and atomic-level design of high-performance SANs for biological environments. Consequently, SANs represent promising biocatalysts for a range of biomedical applications.^48^

**Fig. 1:**
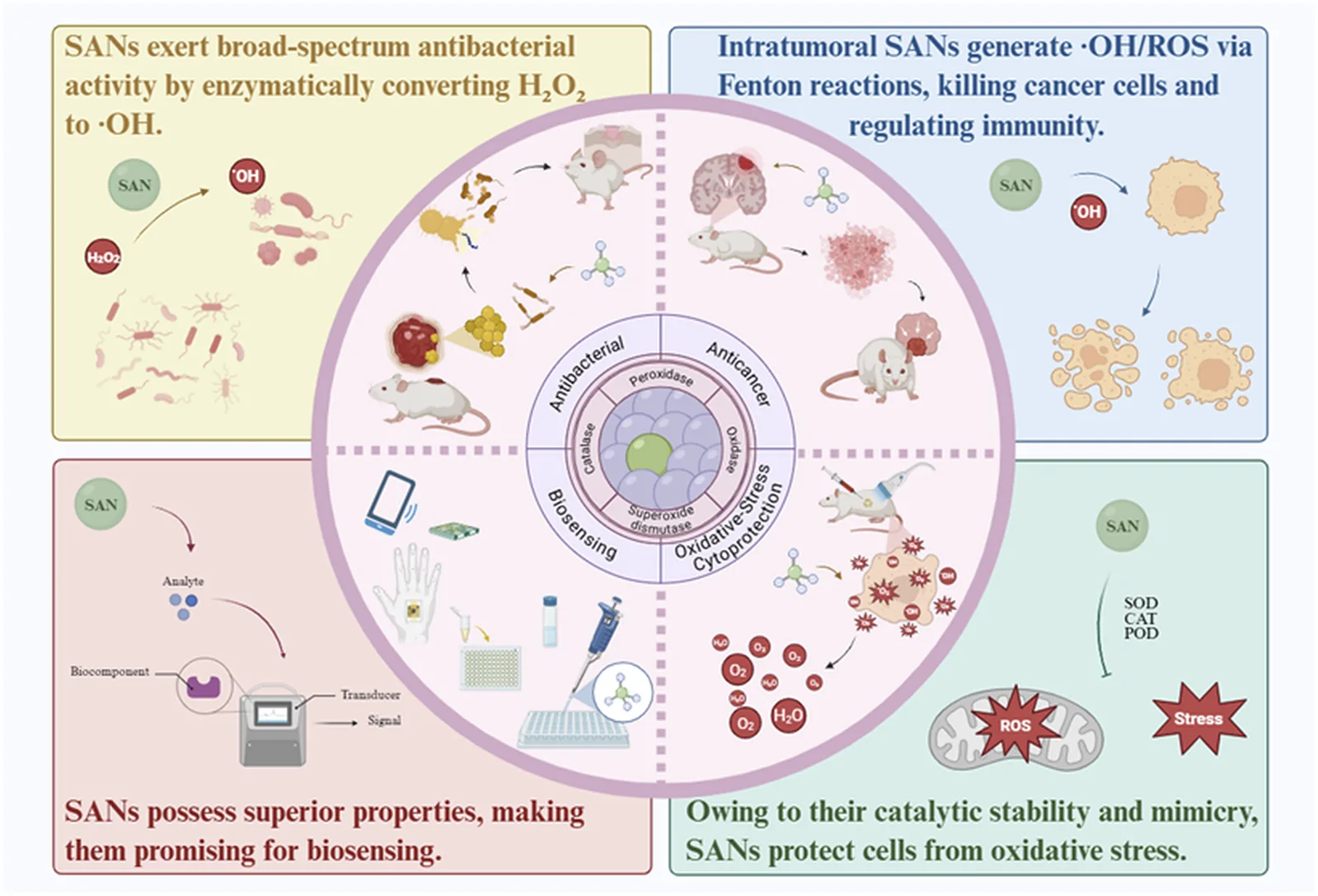
Biomedical applications of single-atom nanozymes. Schematic overview of the major biomedical applications of single-atom nanozymes (SANs), including antibacterial activity, anticancer therapy, biosensing, and regulation of oxidative stress and cytoprotection.

**Fig. 2:**
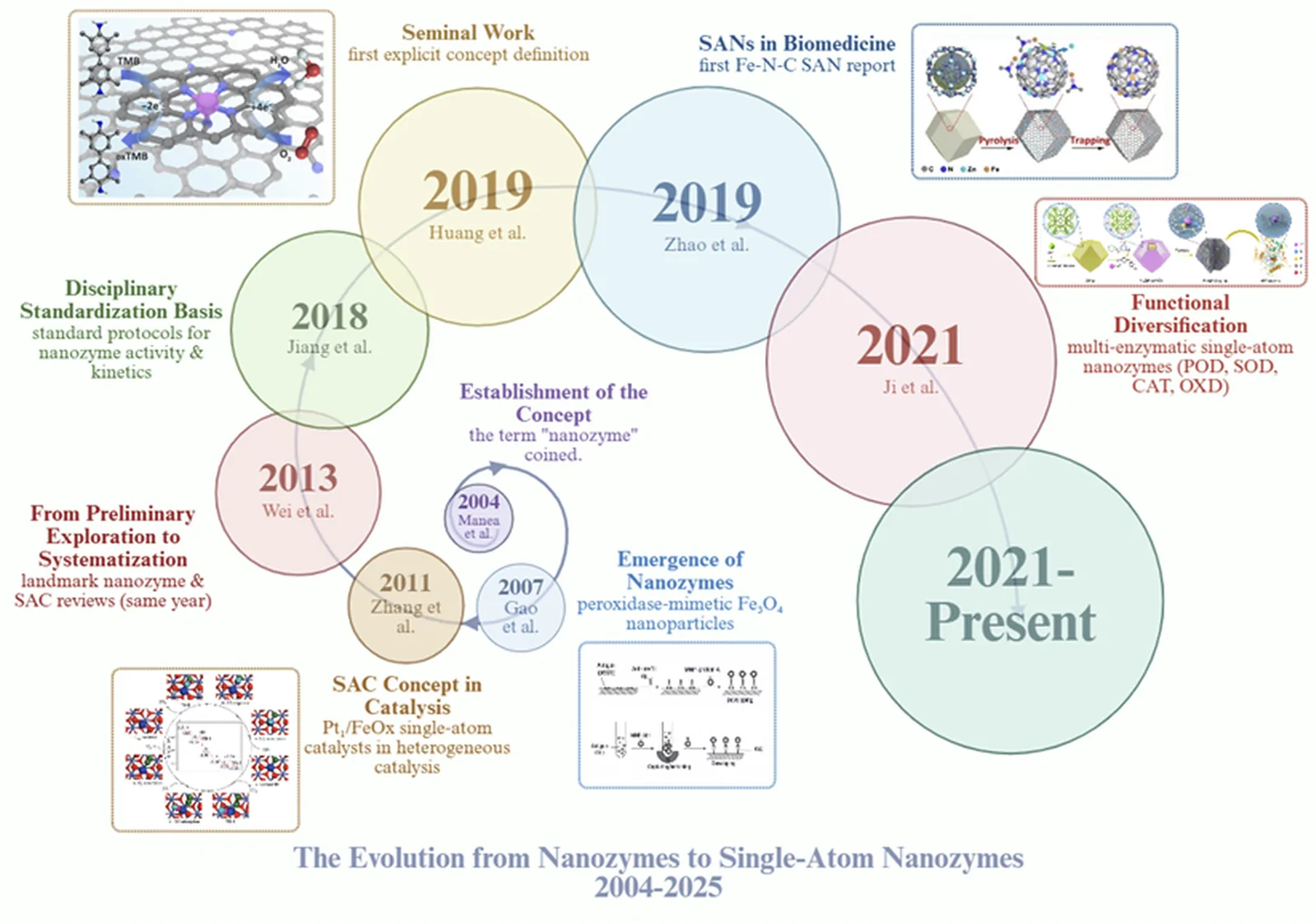
Evolution of single-atom nanozymes. Timeline depicting key milestones in the development of single-atom nanozymes (SANs), from the emergence of single-atom catalysis and the nanozyme concept to recent advances in biomedical applications, spanning 2004–2025.

Existing reviews by Xiang *et al.* and Shen *et al.* have comprehensively examined the biomedical applications of SANs with a focus on their intrinsic enzymatic activities.^49–57^ Zhao *et al.* subsequently evaluated the comparative advantages and limitations of SANs relative to conventional nanozymes, highlighting a “smaller is better” paradigm in which reduced dimensions may enhance catalytic activity and selectivity.^48^ More recently, Chang *et al.* systematically compared SANs, few-atom clusters, and nanoparticles in terms of their electronic structures, geometric configurations, synthesis strategies, and catalytic properties.^58^

Despite these significant contributions, recent advances including spatial-position engineering, dual-atom nanozymes, non-metal single-atom systems, and dynamic coordination engineering have substantially expanded the design principles and functional capabilities of SANs, warranting an updated and more critical perspective. Accordingly, this review adopts a mechanism-driven framework that emphasizes how atomic coordination environments and electronic structures influence catalytic behaviour and biomedical performance. Rather than providing another descriptive overview, we critically compare the catalytic characteristics of single-atoms, few-atom clusters, nanoclusters, and nanoparticles across different reaction systems, highlighting when isolated active sites are advantageous and when cooperative multi-site catalysis may offer enhanced performance. We further discuss how these structure–activity relationships translate into biomedical applications, including antimicrobial therapy, cancer treatment, biosensing, and oxidative stress regulation, while critically evaluating the remaining challenges in catalytic stability, biosafety, scalable synthesis, reproducibility, and clinical translation. By integrating recent advances with mechanistic insights and translational perspectives, this review provides a complementary and forward-looking framework for the rational design and future clinical translation of next-generation single-atom nanozymes.

## 2. Synthetic strategies of SACs

The synthesis methods strongly influence the atomic structure and coordination environment of SACs, thereby determining their active-site geometry, catalytic activity, and selectivity.^59^ However, SACs generally require more stringent synthesis and characterization than nanoparticles, creating substantial challenges in controlling metal loading, stability, and structural uniformity. As SAC synthesis has already been extensively reviewed,^60–64^ this section does not aim to provide a comprehensive account. Instead, we briefly summarize the principal synthetic approaches and then focus specifically on strategies relevant to SANs for biomedical applications. It is important to distinguish conventional heterogeneous SACs used in energy conversion and gas-phase catalysis from biomedical single-atom nanozymes (SANs) designed to mimic natural enzymes. Although these systems share some synthetic approaches, their design principles, support selection, process parameters, and post-treatment requirements differ considerably. Moreover, catalytic activity and selectivity may vary markedly with atomic nuclearity and particle size because of changes in geometric and electronic structures; therefore, no universal size–performance relationship applies across all catalytic systems. We first outline the main synthesis methods, followed by three key considerations for SAN fabrication—metal loading, stability, and structural precision—before systematically comparing SAC and SAN synthesis requirements.

### 2.1 Overview of principal synthesis methods for SACs

Universal synthetic strategies capable of producing multi-element SACs on diverse substrates using a single method significantly reduce application barriers. Such strategies provide versatile SAC platforms, which are easy to operate and exhibit excellent applicability across diverse scenarios.

#### 2.1.1 Pyrolysis strategy

Pyrolysis is widely used to synthesize carbon-supported SACs, with metal–organic frameworks (MOFs) frequently employed as precursors or sacrificial templates (Fig. 3a).^65–68^

**Fig. 3:**
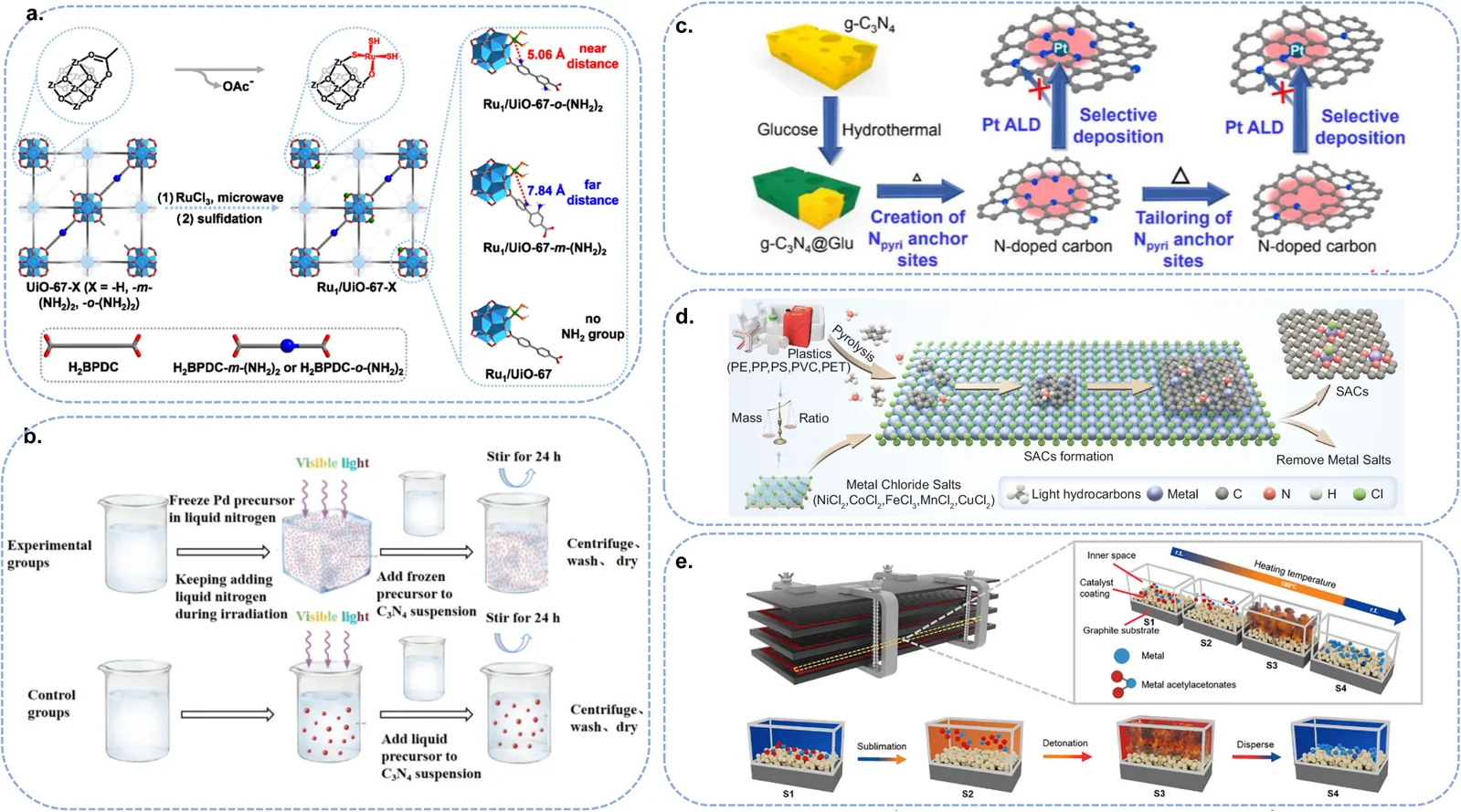
Schematic illustration of representative synthetic strategies for single-atom catalysts. (a) Illustration showing the synthetic strategy for Ru_1_/UiO-67-X (X = -H, -*m*-(NH_2_)_2_, -*o*-(NH_2_)_2_).^68^ Copyright 2024, American Chemical Society. (b) Schematics illustrating the preparation of the experimental groups of SAC samples using the frozen precursor solution and the control groups using the liquid precursor solutions in photochemical routes.^71^ Copyright 2023, Royal Society of Chemistry. (c) Schematic illustration of the synthesis of Pt_1_/NC SACs.^72^ Copyright 2021, American Chemical Society. (d) Schematic illustration of SACs derived from plastics and transition metal salts. PE, PP, PS, PET and PVC denote polyethylene, polypropylene, polystyrene, polyethylene terephthalate and polyvinyl chloride respectively.^73^ Copyright 2025, Springer Nature. (e) Structure of the micro-explosion reactor and the internal catalyst synthesis procedure and schematic representation of the different stages of catalyst synthesis using the MD method.^74^ Copyright 2025, Wiley-VCH GmbH.

A typical synthesis involves incorporation of metal precursors, controlled pyrolysis under an inert atmosphere, and post-treatment to obtain carbon-supported SACs. For example, Huang *et al.* prepared Fe SACs with Fe–N_5_ configurations *via* MOFs at 900 °C,^19^ while Yin *et al.* achieved Co SACs with 4 wt% loading using bimetallic Zn/Co-MOFs.^69^ Zhao *et al.* extended this approach to Ni SACs using CdS@3-AF nanostructures.^70^ Although pyrolysis enables efficient SAC synthesis, two major challenges persist. One challenge lies in achieving uniform dispersion of SACs within biological matrices. Another is the need for precise surface functionalization to meet medical application requirements. Ongoing efforts aim to address these issues through post-synthetic modifications that improve dispersibility and surface functionality.

Pyrolysis is the most widely adopted method for SAN synthesis because the carbon matrix derived from MOF precursors offers structural tunability, but its biocompatibility is not universal and typically requires careful design and post-synthetic surface functionalization to ensure favorable biological performance. However, the high temperatures required (typically 700 to 1000 °C) can lead to uncontrolled nitrogen doping patterns and metal aggregation, which directly affect enzyme-mimetic activity. For biomedical translation, the pyrolysis conditions must be optimized not only for metal dispersion but also for the retention of catalytically favorable coordination configurations (such as M–N_*x*_), as these significantly influence the specific ROS-generating pathways in physiological environments.

#### 2.1.2 Wet-chemistry approach

Wet-chemical synthesis is particularly suitable for SANs. This approach typically involves immobilization of metal precursors (*e.g.*, Fe^3+^, Pt^2+^) onto supports, followed by thermal treatment to stabilize single-atom sites.^75,76^ For instance, Cheng *et al.* anchored Fe–N_4_ sites on carbon nanotubes *via* this method.^77^

Co-precipitation and related approaches have also been applied,^78,79^ though these variants are less commonly used for SANs due to challenges in maintaining atomic dispersion during processing.

Despite these advances, significant challenges remain for the biomedical application of SACs. For instance, intravenously administered SACs with larger hydrodynamic sizes may be rapidly cleared by the reticuloendothelial system, thereby compromising their catalytic efficacy. The wet-chemistry approach offers distinct advantages for SANs because it operates under mild conditions and enables direct integration with biocompatible coatings or targeting ligands during synthesis. However, the weak binding strength between metal precursors and supports often results in metal aggregation during subsequent processing. For biomedical applications, strategies that strengthen metal–support interactions under aqueous conditions are therefore critical to achieving the stable atomic dispersion required for consistent therapeutic performance.

#### 2.1.3 Atomic layer deposition strategy

Atomic layer deposition (ALD) enables atomic-level precision in controlling metal loading and coordination environment,^65^ making it valuable for fundamental structure–activity studies of SANs.^72,80^ However, its high cost and low throughput restrict biomedical applications, although ALD-derived coatings have shown antimicrobial potential for surface engineering of medical devices.^81–83^ For SAN synthesis intended for therapeutic use, ALD is currently more suitable for mechanistic investigations than for scalable production.

#### 2.1.4 Templated synthesis

Templated synthesis employs sacrificial scaffolds to prevent pore collapse during calcination, generating hierarchically porous structures with high surface areas.^84–86^ This approach is particularly attractive for SANs because the porous architecture facilitates mass transport of substrates in biological environments, while high surface area enhances active-site exposure at low metal loadings. Moreover, the tunable pore structure also allows subsequent loading of therapeutic agents, positioning templated SANs as multifunctional platforms for combined catalytic therapy and drug delivery.^73^ Specific examples include NaCl-templated Fe/Co/Ni SACs,^85^ ZIF-derived Fe–N_4_ catalysts,^86^ and *in situ* templated M–N–C systems,^84^ notably, plastic-derived Cu SACs have demonstrated gram-scale production capability and specific surface areas up to 2795 m^2^ g^−1^ (Fig. 3d),^73^ illustrating the scalability of templated approaches for potential biomedical translation.

#### 2.1.5 Ball milling

Ball milling offers a solvent-free, scalable route for SAN fabrication. The mechanical force can create strong metal–support interactions that stabilize single atoms without high-temperature treatment, potentially preserving catalytically active configurations that might be destroyed by pyrolysis.^87,88^ This simplicity meets the demand for repeatable preparation in biomedical research. However, controlling milling parameters to achieve uniform atomic dispersion without introducing metallic impurities remains challenging, particularly for SANs intended for *in vivo* applications where purity is critical.

#### 2.1.6 Photochemical routes

Photochemical synthesis uses light energy to achieve atomic dispersion under mild conditions (room temperature, atmospheric pressure), offering several advantages for SAN fabrication.^89,90^

The ambient conditions effectively inhibit metal agglomeration and preserve fragile coordination configurations essential for enzyme-like activity. Importantly, the absence of high-temperature treatment enables direct integration of heat-labile biomolecules (*e.g.*, targeting ligands, biocompatible polymers) during synthesis, facilitating the creation of multifunctional SAN platforms.^91–93^ Photochemical routes are also versatile across diverse metal precursors and support materials.^94^ However, strict control of reaction parameters and limited scalability remain challenges for mass production of SANs for therapeutic applications.

Several synthetic strategies have been established for SAC fabrication, as summarized in Table 1. The principal synthetic methods include pyrolysis, wet-chemical approaches, atomic layer deposition, template-assisted synthesis, ball milling, photochemical routes, vacancy-directed synthesis, and chemical vapor deposition. Although each method presents inherent technical limitations, they collectively provide valuable tools for advancing biomedical applications and nanocatalytic systems. Additional strategies, such as surface modification and protective coatings, have also been explored to enhance the biocompatibility of SACs while preserving their catalytic function. These advances address key biocompatibility challenges while preserving the essential catalytic functions required for therapeutic intervention.

**Table 1:** Synthetic strategies of SACs and their advantages and disadvantages

Synthetic methods	Advantages	Disadvantages	Ref.
Pyrolysis strategy	Operationally simple	Uncontrollable structure	111
Atomic layer deposition	Controlled deposition	Expensive	65
Chemical vapor deposition	Precise structure and high purity	Limited productivity	65
Vacancy-directed synthesis	Clear coordination structure	Manufacturing complexity and low throughput	65
Impregnation method	Convenient to synthesize	Poor controllability	61
Coprecipitation method	Uniform loading	Low binding strength	112
Electrochemical displacement deposition	Inexpensive and time-saving	Complicated synthesis	113
Direct conversion	Mild reaction conditions	Easy to agglomerate	114
Ion exchange method	Convenient to synthesize	Low metal loading	115
Redox method	Wide applicability	Complicated synthesis	116
Ball milling	Green, scalable synthesis	Precise milling parameter control	117
Photochemical routes	Efficient single-atom dispersion	Stringent operating conditions	71

### 2.2 Synthesis of SACs with high metal loading

For biomedical applications, high metal loading is not always necessary. SANs with relatively low loadings (typically 0.1–2 wt%) can achieve potent therapeutic effects because the atomically dispersed nature ensures nearly every metal atom is accessible as a catalytic site. Moreover, lower loading reduces the risk of metal ion leakage upon partial degradation in physiological environments, directly addressing a critical biosafety concern that is less relevant for industrial catalysis.

In industrial catalysis, by contrast, high-metal-loading SACs enhance catalytic activity through dense active sites,^95^ but this approach may increase metal aggregation risks.^96^ Various approaches have achieved loadings exceeding conventional limits, including vacuum-annealed Pt SACs (up to 44.8 wt%),^97^ stepwise pyrolyzed Pt SACs (5 wt%),^98^ and Fe-N/C catalysts (11.8 wt%).^99^ Beyond these, a coordination-polymerization method has enabled Cu SAC production at 3.565 kg per batch with 16.03 mol% loading, demonstrating industrial scalability.^100^ The distinction between industrial and biomedical requirements underscores that SAN design should prioritize catalytic efficiency per metal atom and biosafety over maximizing total metal content.

### 2.3 Synthesis of SACs with high stability

For industrial SACs, stability typically refers to resistance to sintering and leaching under harsh catalytic conditions.^101^ Various strategies have been developed to enhance stability, including micro-burst synthesis for rapid metal anchoring (Fig. 3e),^74^ mesopore confinement in carbon matrices,^102^ vacancy-directed stabilization on MXenes,^103^ and olefin-mediated aggregation prevention.^104^

The concept of stability in SANs, however, differs fundamentally from that in conventional SACs. For biomedical applications, stability encompasses: (1) resistance to metal ion release in physiological environments; (2) maintenance of catalytic activity in the presence of biological macromolecules (*e.g.*, proteins, glutathione); and (3) controlled degradation behavior to avoid long-term accumulation. The strategies that emphasize strong metal–support interactions—such as robust M–N_*x*_ coordination and surface passivation—are directly relevant to SANs, as these interactions help prevent metal leakage in biological settings.^105^ This distinction is critical for designing SANs with both catalytic efficacy and biosafety.

### 2.4 Synthesis of SACs with well-defined structures

Structurally defined SACs offer an ideal platform for establishing structure–activity relationships. MOFs and COFs are widely used as host structures because their coordination networks contain uniformly distributed metal nodes with well-defined geometries.^106^

For biomedical applications, MOF-derived SANs have demonstrated therapeutic potential. Yu *et al.* constructed Fe/MOF-derived SANs.^107^ Qin *et al.* developed bimetallic ZnRu-MOFs with twin-phase structures that catalyze specific ROS generation, enabling biosensing of cardiovascular biomarkers when integrated with Pt SACs (Fig. 4).^108^ Among MOF-based structures, conductive metal–organic frameworks (cMOFs) exhibit high porosity and electrical conductivity. Park *et al.* showed that Pd SAC-functionalized cMOFs preserve porosity while stabilizing single atoms *via* Pd–N_4_ coordination.^109^ Pyrolysis-free strategies using COF precursors have also yielded well-defined Fe–N_4_ SACs.^110^

**Fig. 4:**
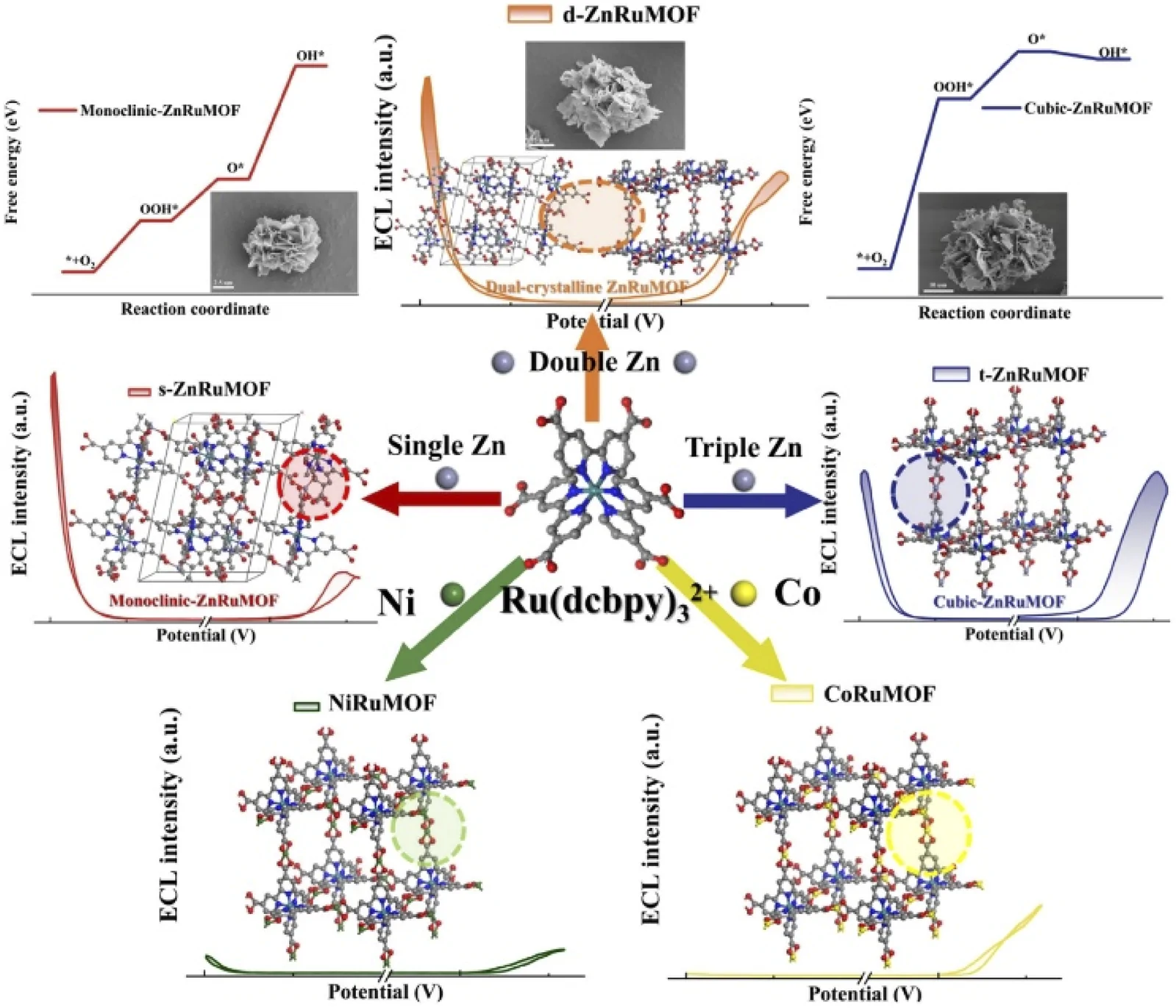
Schematic diagram of the dual-crystalline bimetallic MOF structure with Ru(dcbpy)3Cl_2_ as the main body and different metals as nodes, and comparison with ECL signals. Reproduced with permission.^108^ Copyright 2025, Wiley-VCH GmbH.

Despite the advantages of MOFs and COFs for constructing structurally precise SACs, their high synthesis cost remains a barrier to large-scale application. Thus, developing more economical preparation strategies is urgently needed. Beyond mechanistic studies, well-defined SAN structures offer a unique advantage for biomedical translation. The precise coordination environment enables systematic structure–activity relationship studies that link atomic-level features to biological outcomes, facilitating regulatory evaluation. However, the high cost and low throughput of certain precise synthetic methods limit their practical translation, highlighting the need for cost-effective strategies that retain atomic-level precision while remaining compatible with biomedical manufacturing standards.

### 2.5 A systematic comparison of synthesis strategies: SACs and SANs

While the preceding sections have surveyed mainstream synthetic strategies for SACs, industrial heterogeneous SACs and SANs represent two fundamentally distinct material systems, differing in design rationale, material selection, synthetic constraints, and post-treatment requirements.

Industrial SACs are tailored for high-temperature gas–solid reactions such as oxidation, electrocatalysis, and hydrogenation, where the overriding priorities are high active-site density, thermal robustness, and resistance to sintering and coking;^118^ biomimetic activity is neither required nor expected. SANs, by contrast, are engineered to mimic the M–N_*x*_ coordination centers of natural metalloenzymes and to function under physiological conditions.^119,120^ These divergent goals dictate distinct synthetic protocols.

The two systems also diverge in precursor and support selection. Industrial SACs can be deposited on a wide range of supports, including toxic or non-degradable metal oxides, carbon matrices, and zeolites.^121^ For SANs, however, the substrate must be biocompatible. Nitrogen-doped carbons derived from ZIF-8 or polydopamine are widely adopted for their favorable biocompatibility and structural tunability, while only low-toxicity metal oxides are permissible and toxic heavy-metal precursors are excluded to forestall metal leakage and organ accumulation *in vivo*.^122^

Synthetic constraints further separate the two classes. Industrial protocols routinely involve ultra-high-temperature calcination and toxic templates, with little need for post-synthetic modification. Synthesizing SANs demands precise control over pyrolysis temperature to preserve the desired coordination environment. Heteroatom doping is commonly employed to tune the electronic configuration of the metal center, and post-synthetic functionalization, most notably PEGylation,^123^ is performed to optimize *in vivo* performance and biocompatibility.^124^

Another distinguishing feature is the tolerance to metal clusters. In industrial catalysis, metal clusters are typically eliminated to prevent side reactions and ensure selectivity. SANs, on the other hand, can accommodate or even deliberately construct diatomic or few-atom clusters, thereby enabling synergistic biocatalysis.^125,126^ This departure from the conventional size-reduction paradigm of industrial SACs constitutes a key insight from structure–activity studies of nanozymes.

Finally, rigorous post-processing and biosafety optimization are mandatory for SANs but unnecessary for industrial SACs. The former requires thorough impurity removal, hydrophilic modification, and systematic biotoxicity evaluation, including assessments of metal leaching, cytotoxicity, hemocompatibility, and *in vivo* clearance, to meet the demands of clinical translation.^127^

Taken together, although SANs share fundamental synthetic concepts with industrial SACs, they demand a distinct, biology-centered design philosophy tailored for biomedical applications.^128^

## 3. Characterization techniques of SACs

The atomically dispersed nature of single-atom catalysts (SACs) presents unique characterization challenges that cannot be adequately addressed using conventional techniques alone. Unlike nanoparticles, SACs require complementary atomic-resolution imaging and synchrotron-based spectroscopic analyses to unambiguously determine the dispersion, coordination environment, oxidation state, and electronic structure of isolated metal atoms, which are essential for establishing reliable structure–activity relationships. Therefore, an integrated characterization strategy combining microscopic, spectroscopic, and theoretical approaches has become indispensable for validating SAC structures and understanding their catalytic mechanisms. The principal characterization techniques employed for the structural and electronic analysis of SACs are summarized in Table 2.

**Table 2:** Characterization methods of SACs

Characterization techniques	Atomic-level information obtained	Role in SAC characterization	Ref.
HAADF-STEM	Direct visualization of isolated metal atoms as bright atomic contrast	Confirms atomic dispersion and excludes nanoparticle aggregation; often combined with EDS/EELS for elemental mapping	16
HRTEM	Crystal structure, lattice fringes, defects, and interfaces	Characterizes support crystallinity and structural defects but cannot independently verify isolated single atoms	10
XAFS	Local coordination environment, oxidation state, electronic structure, coordination number, bond distances, and neighboring atomic species surrounding isolated metal atoms	Provides comprehensive atomic-scale structural and electronic information, validates atomic dispersion, and establishes structure–activity relationships by combining XANES and EXAFS analyses	134
XANES	Oxidation state, electronic configuration, and coordination symmetry	Determines valence state and local electronic environment of single metal atoms	135
EXAFS	Coordination number, bond length, and neighboring atomic species	Quantitatively determines local coordination environment (*e.g.*, M–N_4_, M–O_4_)	136
FT-EXAFS	Radial distribution of neighboring atoms and metal–metal interactions	Confirms atomic dispersion by the absence of M–M coordination and enables quantitative fitting of coordination structures	137
XRD	Crystal phase and crystallinity	Characterizes support structure and excludes crystalline metal nanoparticles, although insensitive to isolated atoms	138
XPS	Surface elemental composition, chemical state, and electronic interaction	Evaluates surface chemistry and metal–support electronic interactions	139
DFT	Electronic structure, charge distribution, adsorption energy, and reaction pathway	Interprets experimental observations and establishes atomic-level structure–activity relationships	140

Aberration-corrected high-resolution transmission electron microscopy (AC-HRTEM) and HAADF-STEM have been established as invaluable tools in the field. AC-HAADF-STEM can capture clear images of SACs at atomic resolution. This technique allows researchers to directly observe single metal atoms and clearly identify their spatial distribution. Qiao *et al.* successfully utilized this technique to confirm the homogeneous dispersion of Mn atoms within Mn-N/C nanozyme structures.^29^ Complementing this approach, Xu *et al.* combined HAADF-STEM with energy-dispersive X-ray spectroscopy (EDS) elemental mapping to verify the uniform distribution of Ru atoms in UiO-67-Ru catalysts.^129^

Although atomic-resolution microscopy confirms the presence of isolated atoms, it cannot determine their local coordination environment or electronic structure. These crucial structural parameters are obtained using synchrotron-based X-ray absorption spectroscopy (XAS), including X-ray absorption near-edge structure (XANES) and extended X-ray absorption fine structure (EXAFS). XANES provides information on oxidation state and electronic configuration, whereas EXAFS quantitatively determines the coordination number, bond distance, and neighboring atomic species surrounding the active metal center. For example, Zhao *et al.* applied EXAFS to confirm Ni–N coordination in Ni SAs@S/N-FCS catalysts and identified their intermediate oxidation state.^70^ Yang *et al.* further demonstrated the combination of AC-HAADF-STEM images with XANES/EXAFS data to validate atomic dispersion in Fe-SAs/N-C systems.^130^

In addition to the aforementioned characterization techniques, Fourier-transform EXAFS and FTIR spectroscopy provide complementary insights into the local coordination environment and catalytic processes of SACs. FT-EXAFS is particularly valuable for confirming atomic dispersion by distinguishing isolated metal atoms from metal clusters or nanoparticles through the absence of metal–metal coordination, while quantitative fitting further determines coordination numbers and bond lengths. Meanwhile, *in situ* FTIR spectroscopy enables real-time monitoring of surface adsorbates and reaction intermediates, providing mechanistic insights into catalytic pathways. For example, Zhang *et al.* developed a nanoisland-encapsulated single cobalt atom catalyst (Co_SA/ZnO_–ZnO), in which HAADF-STEM, XRD, XANES, and FT-EXAFS analyses (Fig. 5) collectively confirmed the formation of isolated Co sites on ZnO nanoislands responsible for its highly efficient Fenton-like catalytic activity.^131^

**Fig. 5:**
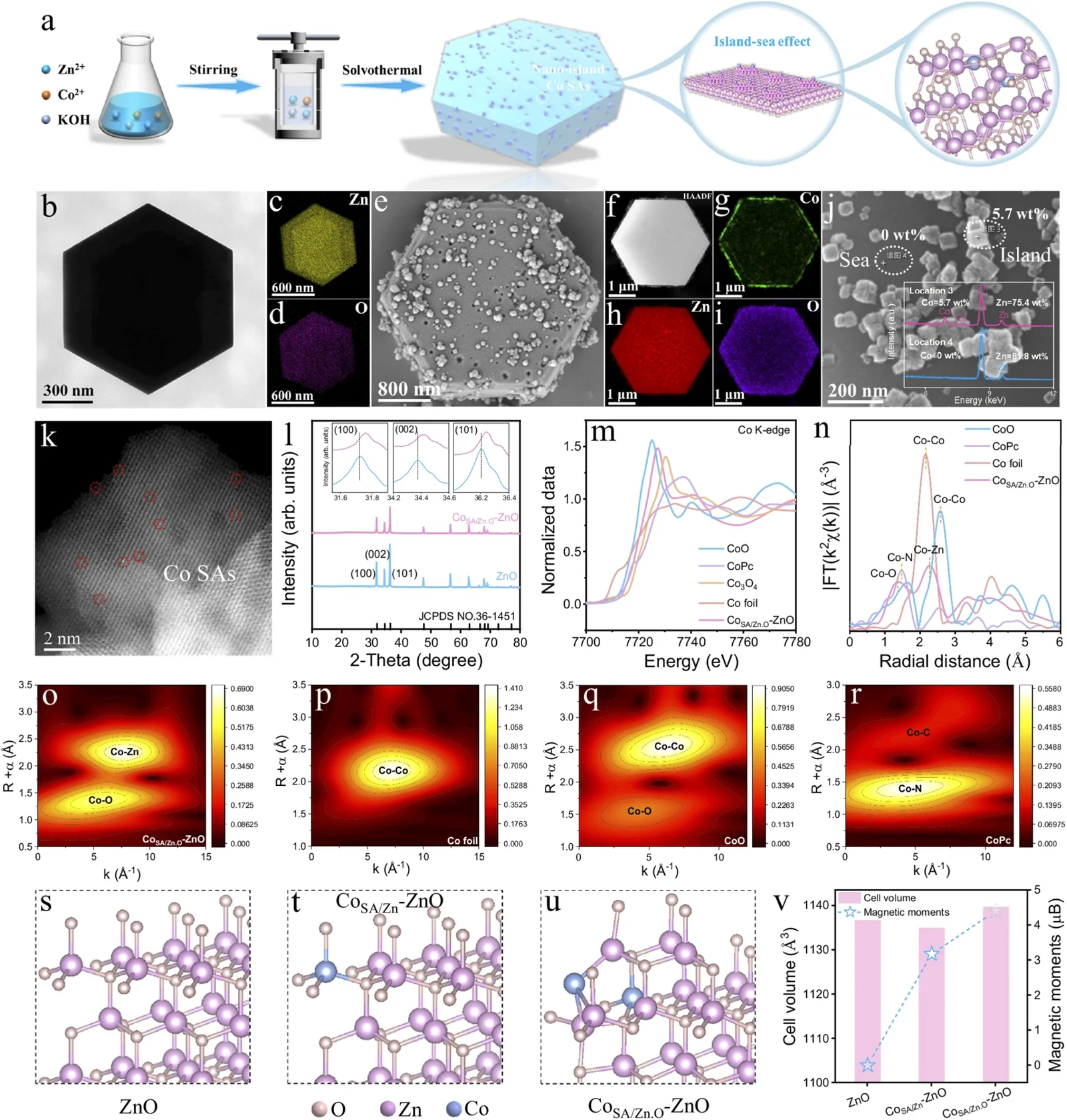
Validation of the nano-island structure of Co SACs. (a) Synthesis procedure of Co_SA/ZnO_–ZnO. (b) TEM image of ZnO. TEM elemental mapping of ZnO with (c) Zn and (d) O. (e) SEM image of Co_SA/ZnO_–ZnO. HAADF-STEM elemental mapping of Co_SA/ZnO_–ZnO with (f) original, (g) Co, (h) Zn, and (i) O. (j) SEM image of small particles on the surface of Co_SA/ZnO_–ZnO (inset: distribution of Co content in different locations of Co_SA/ZnO_–ZnO catalyst). (k) HAADF-STEM image of Co_SA/ZnO_–ZnO (Co SAs marked by red circles). (l) XRD pattern of ZnO and Co_SA/ZnO_–ZnO (inset: enlarged view of (100), (002) and (101) crystal facets). (m) XANES spectra of Co_SA/Zn.O_–ZnO at the Co K-edge. (n) FT k_3_-weighted EXAFS spectra of Co_SA/ZnO_–ZnO and references. WT-EXAFS for K-edge for (o) Co_SA/ZnO_–ZnO, (p) Co foil, (q) CoO and (r) CoPc. Three configurations of (s) monomer ZnO, (t) Co replaced Zn and (u) Co replaced Zn, O. (v) Comparison of the cell volume and magnetic moment of the three configurations. Reproduced with permission.^131^ Copyright 2025, Springer Nature.

Alongside experimental characterization, density functional theory (DFT) calculations have become indispensable for understanding the electronic structure and catalytic mechanisms of SACs.^132^ By modeling atomic structures, electronic configurations, adsorption energies, charge transfer, and reaction pathways, DFT provides atomic-level mechanistic insights that complement experimental observations. For instance, Yang *et al.* employed DFT calculations to investigate charge-transfer behavior during caffeic acid oxidation in Fe SAs/N-C systems, demonstrating that Ni incorporation significantly enhanced electron transfer between the catalyst and substrate, thereby improving catalytic activity.^130^

Fig. 6 further illustrates the importance of integrating multiple characterization techniques to establish reliable structure–activity relationships in single-atom nanozymes. Using the representative Fe-based single-atom catalyst (Fe@BNC-800-L1) recently reported by Ma *et al.*, HAADF-STEM imaging (Fig. 6b and c) directly visualizes uniformly dispersed isolated Fe atoms without detectable aggregation, while XPS (Fig. 6d) confirms Fe–N bond formation through the characteristic Fe–N_*x*_ peak.^133^ XANES analysis (Fig. 6e) identifies the Fe^3+^ oxidation state, whereas the FT-EXAFS spectra with quantitative fitting (Fig. 6f–h) provide strong atomic-level structural evidence. The dominant Fe–N scattering peak (∼1.8 Å), together with the absence of an Fe–Fe coordination peak (∼2.2 Å), supports the absence of detectable metallic Fe or FeO_*x*_ nanoparticles. Quantitative EXAFS fitting further reveals an Fe–N coordination number of 4.6 ± 0.7 and an average Fe–N bond length of 1.96 ± 0.01 Å, confirming the well-defined Fe–N coordination environment responsible for the catalyst's activity. This integrated characterization workflow combines atomic-resolution imaging, synchrotron-based spectroscopy, quantitative EXAFS analysis, and theoretical calculations to establish the structural and electronic features of isolated active sites. Such an approach is indispensable for correlating atomic structure with catalytic performance and provides a foundation for the rational development of next-generation single-atom nanozymes.

**Fig. 6:**
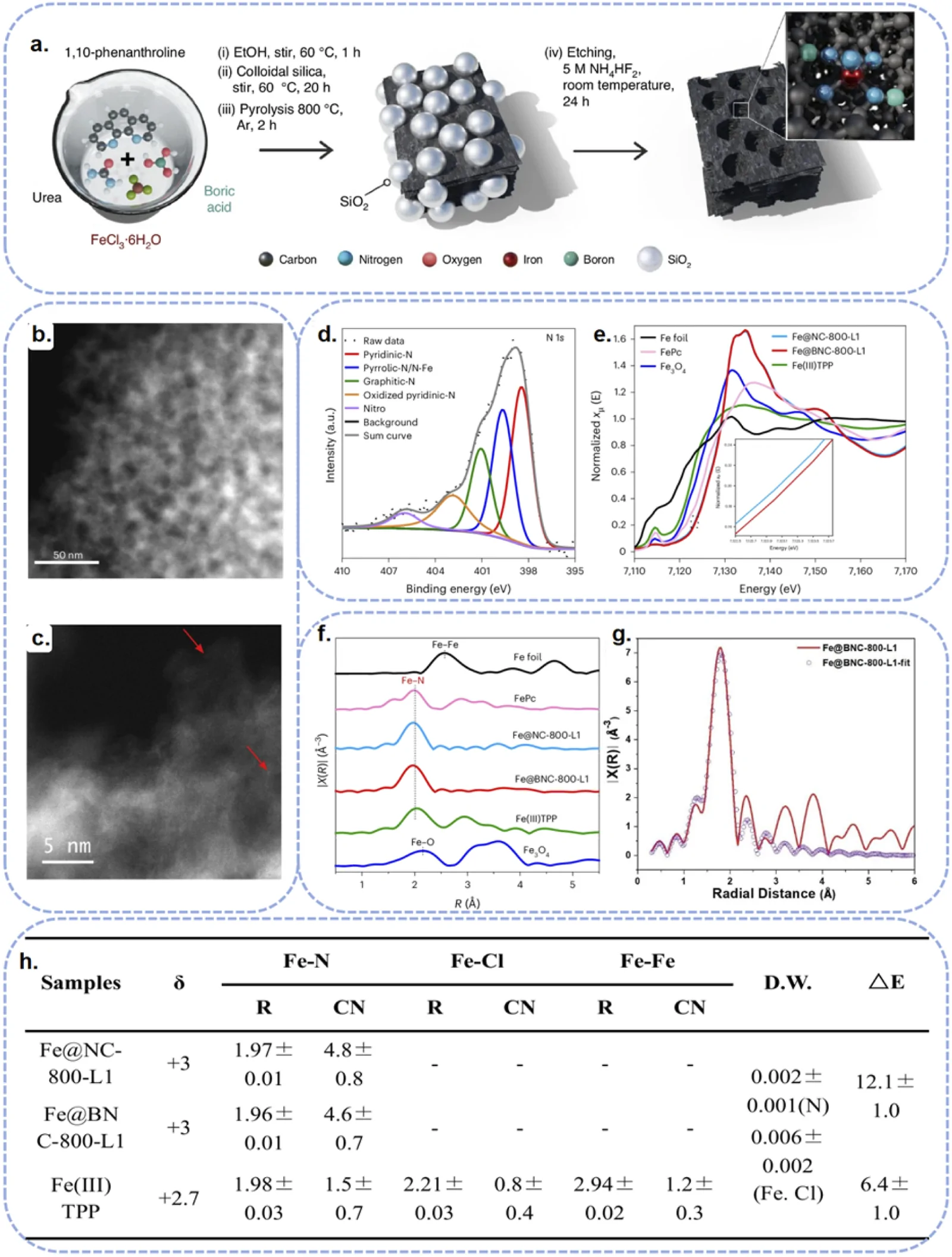
Atomic-level characterization of Fe-based single-atom nanozymes. (a) Schematic of catalyst preparation. (b) and (c), HAADF-STEM image revealing isolated Fe atoms (red arrows) uniformly dispersed on a B, N-co-doped carbon matrix. (d) N 1s XPS spectrum deconvoluted into five components: pyridinic-N (398.4 eV), Fe–N_*x*_ (399.6 eV), graphitic-N (401.5 eV), oxidized pyridinic-N (402.9 eV), and nitro groups (405.9 eV). (e) XANES spectrum confirming the Fe^3+^ oxidation state by comparison with reference compounds (Fe foil, Fe_2_O_3_, and FePc). (f–h) Fourier-transformed k^2^-weighted EXAFS spectra in R-space (experimental data, black line; fitted curve, red line). The dominant peak at ∼1.8 Å corresponds to Fe–N scattering, while the absence of an Fe–Fe peak at ∼2.2 Å shows no detectable Fe–Fe coordination; together with microscopy, it supports predominantly isolated Fe sites within the limits of these measurements. Quantitative EXAFS fitting yields an Fe–N coordination number of 4.6 ± 0.7 and a mean Fe–N bond length of 1.96 ± 0.01 Å.^133^ Copyright 2026, Springer Nature.

## 4. Catalytic mechanisms of SANs in nanomedicine

The uniform atomic dispersion in SANs enhances catalytic activity, selectivity, and stability. This enables the broad application of SANs in nanomedicine for Fenton-like reactions and POD-like, SOD-like, CAT-like, and OXD-like activities (Fig. 7). Depending on catalytic function and conditions, SANs can generate ROS for antibacterial/anticancer treatment or scavenge ROS for cytoprotection and anti-inflammatory therapy Given that such catalytic functions are mediated by metal species of varying nuclearity, it is instructive to examine how catalytic behaviour changes when the active sites transition from nanoparticles to nanoclusters to isolated single atoms.

**Fig. 7:**
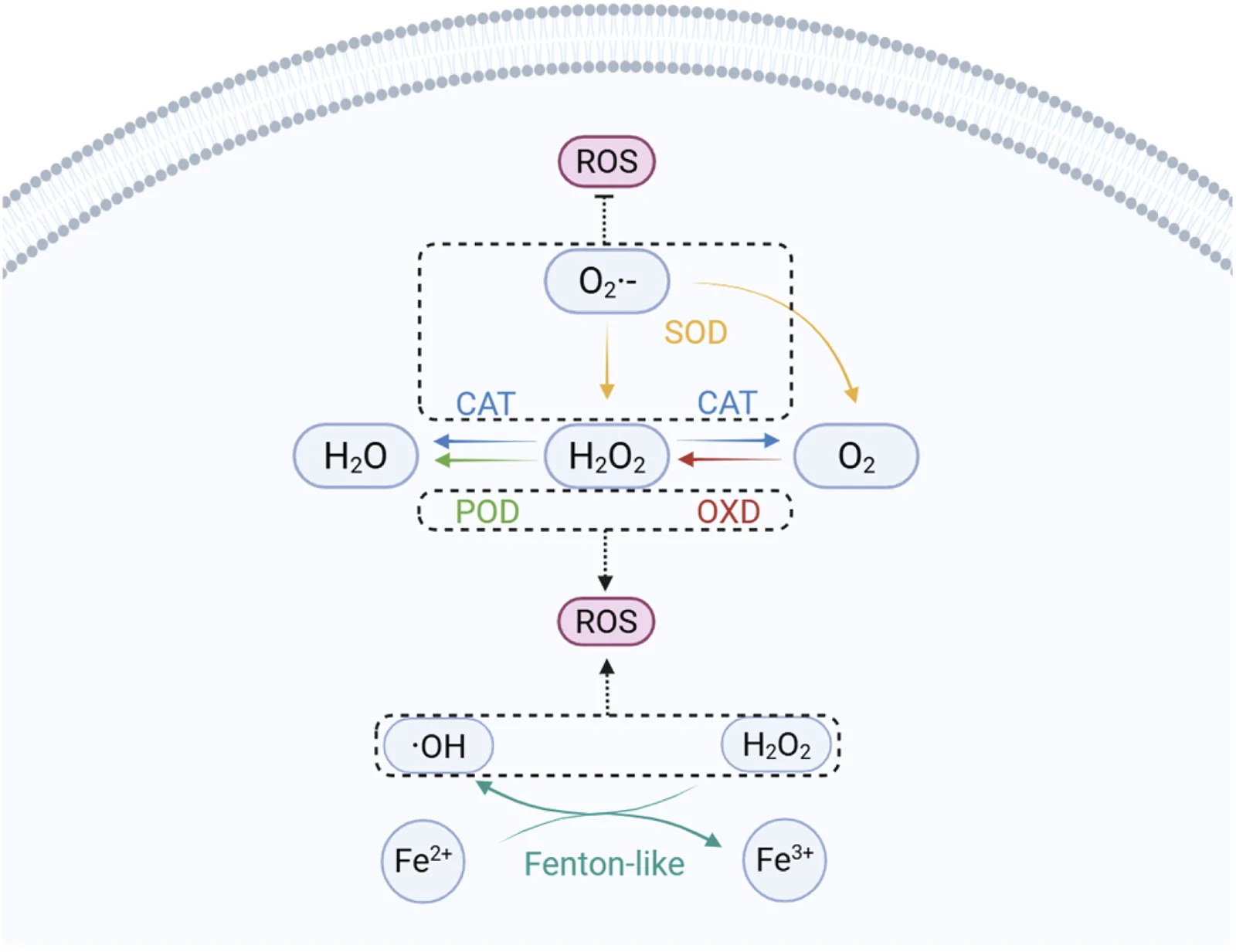
Schematic overview of the major enzyme-mimetic activities of SANs in regulating reactive oxygen species (ROS). SANs can exhibit Fenton-like activity by catalysing the conversion of H_2_O_2_ into highly reactive ·OH through Fe^2+^/Fe^3+^ redox cycling. They can also mimic SOD, CAT, POD, and oxidase (OXD), enabling the catalytic conversion of O_2_·^−^, H_2_O_2_, H_2_O, and O_2_. Depending on their catalytic activity and reaction environment, SANs can either generate ROS for oxidative applications or scavenge ROS to alleviate oxidative stress. The figure was created using https://BioRender.com.

### 4.1 Fenton-like reaction

The Fenton-like reaction, a catalytic process in which transition metal ions (*e.g.*, Fe^2+^) activate peroxides (*e.g.*, H_2_O_2_) to generate ·OH and ROS, serves as a key mechanism for biomedical applications of SANs.^141,142^ The catalytic efficiency and selectivity of this reaction are governed by the coordination environment of the metal center, which determines both the binding affinity toward peroxides and the subsequent radical generation pathway. Li *et al.* systematically investigated Co SACs with Co–N_4_ coordination anchored on N-doped graphene for peroxymonosulfate (PMS) activation.^143^ Their DFT calculations revealed that the CoN_4_ site provides optimal binding energy for PMS activation, while adjacent pyrrolic nitrogen sites facilitate adsorption and subsequent oxidation of organic molecules. This dual-active-site structure elucidates a key mechanistic pathway for achieving high-efficiency Fenton-like catalytic performance, which is fundamental for the effective generation of ROS in therapeutic contexts.

In physiological environments, SANs exhibit enhanced Fenton-like catalytic activity and generate ROS in a targeted manner, showing strong potential to inhibit tumor and bacterial growth. The therapeutic advantage of SANs over conventional nanomaterials lies in their ability to confine ROS generation to pathological tissues through microenvironment-responsive catalysis. While conventional metal nanoparticles (*e.g.*, Fe, Cu, Mn) may induce excessive ROS accumulation *via* mitochondrial membrane potential disruption, potentially causing adverse effects,^144^ these catalysts can utilize endogenous H_2_O_2_ under the mildly acidic conditions often found in the tumor microenvironment (TME) to generate ·OH through Fenton-like reactions, leading to apoptosis and necrosis of tumor cells. However, TME acidity and H_2_O_2_ levels can vary substantially across and within tumors.

The catalytic efficiency of Fenton-like SANs is further determined by the identity of the metal center and its electronic coupling with the support. Fe-based SANs with Fe–N_4_ coordination are designed for tumor therapy and demonstrate substantially higher catalytic efficiency than Fe_3_O_4_ nanoparticles due to optimized metal–support interactions, while other metal centers also play important roles and show considerable potential in related applications.^145^ Expanding beyond iron systems, Lu *et al.* developed copper SANs (Cu-HNCS) where isolated Cu atoms catalyze O_2_/H_2_O_2_ conversion into superoxide ions (O_2_·^−^) and ·OH. In an acidic tumor microenvironment, this process enhances the inhibition of tumor growth. It is noteworthy that the Fenton-like reaction turnover frequency of Cu in Cu-HNCS was approximately 5000 times higher than that of Fe in Fe_3_O_4_ nanoparticles.^146^ These comparative studies establish that the choice of metal center must be matched to the target application, with Cu-based systems offering superior intrinsic activity for Fenton-like catalysis while Fe-based systems provide a more established safety profile. In conclusion, this capability highlights SANs' dual therapeutic potential in both tumor suppression and antibacterial applications. Nevertheless, further investigation is required to expand their biomedical utility.

### 4.2 Enzyme-mimicking catalytic activities

Nanozymes have two fundamental characteristics that determine their enzymatic activity: (1) the capability to mimic multiple enzyme classes, and (2) precisely adjustable catalytic performance. Similar to natural enzyme systems, these artificial enzymes demonstrate catalytic functionalities analogous to several natural enzyme classes including oxidoreductases, hydrolases, lyases, and isomerases. Among these, redox-type nanozymes represent the most extensively studied group, exhibiting POD-like, SOD-like, CAT-like, and OXD-like activities. In 2018, Ma *et al.* reported the pioneering finding that SACs exhibit enzyme-mimetic activities.^147^ The catalytic mechanism and substrate specificity of SANs are dictated by the electronic structure of the isolated metal sites, which determines the activation barrier for each distinct enzymatic pathway.

#### 4.2.1 Peroxidase (POD)-like activity

This mechanism enables biological effects, such as tumor cell elimination under physiological conditions. For POD-like activity, the catalytic efficiency is governed by the ability of the metal center to facilitate O–O bond cleavage in H_2_O_2_, a process that is highly sensitive to the coordination geometry and heteroatom doping of the active site. SANs exhibiting POD-like activity utilize various metal centers, including Fe, Cu, Mn, and Ru. For example, Fe-based SANs with Fe–N–C configurations demonstrate POD-like activity, although their catalytic efficiency may more closely resemble that of natural metalloenzymes than that of conventional nanozymes.^51^ Ji *et al.* prepared FeN_3_P SANs. These materials showed marked POD-like activity in both *in vitro* and *in vivo* tests. These catalysts could selectively enhance oxidant production in acidic tumor microenvironments, providing a practical therapeutic approach to inhibit tumor growth.^21^ Similarly, Zhang *et al.* anchored Fe single atoms on heteroatom-doped carbon nanospheres, synthesizing atomically dispersed FeN_3_P-SAN with POD-like activity and kinetics comparable to those of natural enzymes (Fig. 8). This nanozyme exhibited significant tumor penetration and synergized chemotherapy with photothermal effects to inhibit tumor cell proliferation *in vitro*.^148^ The successful implementation of phosphorus doping in both studies highlights a general strategy: introducing heteroatoms with distinct electronegativity into the first or second coordination sphere can modulate the electron density at the metal center, thereby fine-tuning the activation energy for H_2_O_2_ decomposition. Manganese-based SANs also show significant potential. Qiao *et al.* systematically compared Mn-N/C SANs with MnO_2_ nanoparticles by measuring the absorbance of 3,3′,5,5′-tetramethylbenzidine oxidation at 652 nm. Their results revealed pH-dependent POD-like catalytic activity, with significant activity observed under acidic conditions but not under alkaline conditions. Furthermore, combined treatment of Mn-N/C and H_2_O_2_ led to time-dependent reduction in viability of CT26 and MC38 tumor cells. Importantly, Mn-N/C exhibited higher catalytic activity than MnO_2_ nanoparticles, demonstrating its enhanced therapeutic potential.^29^

**Fig. 8:**
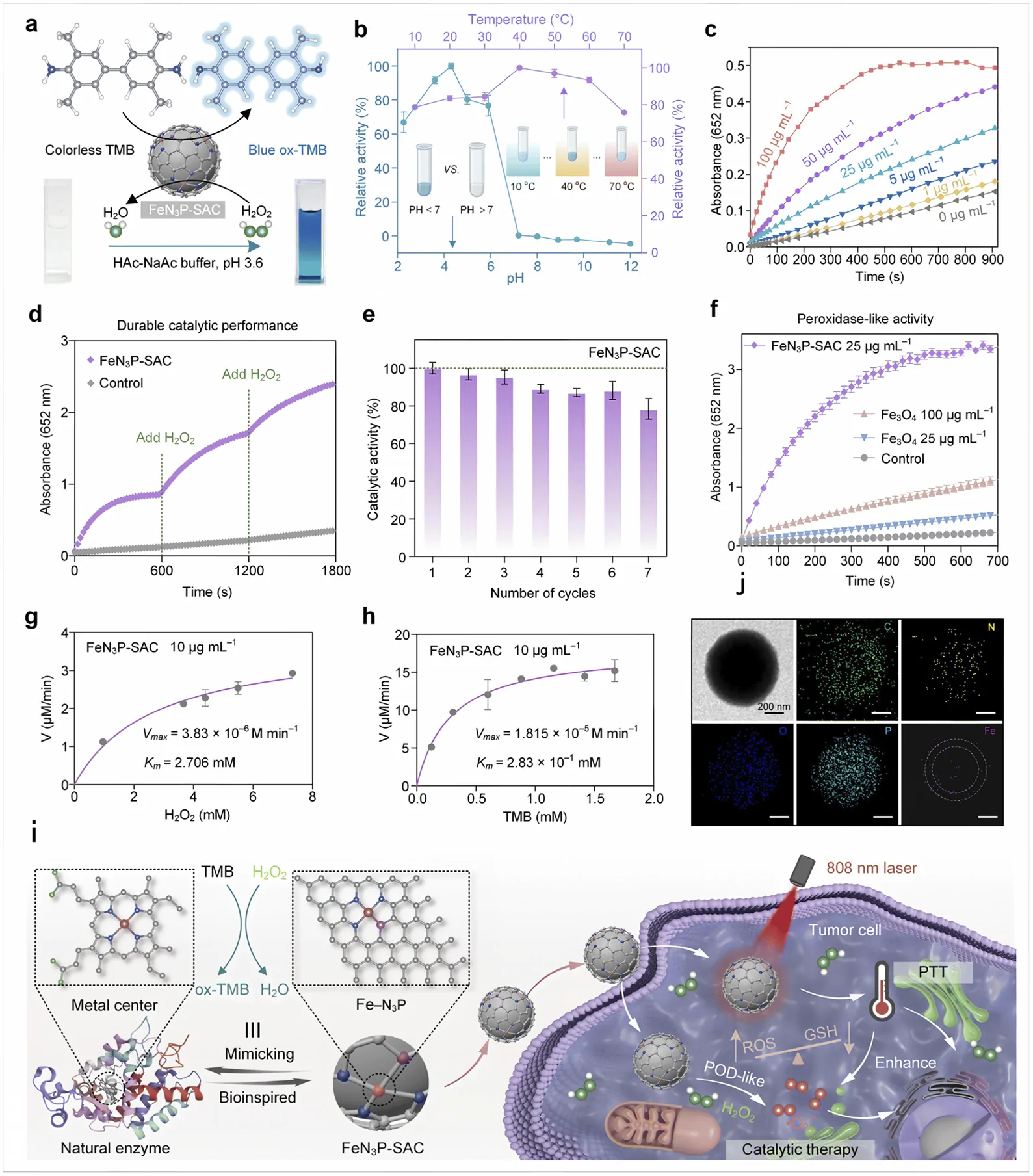
Peroxidase-like properties of FeN_3_P-SAC. (a) FeN_3_P-SAC exhibit POD-like activity by catalyzing the oxidation of peroxidase substrates (TMB) to enable colorimetric reactions. (b) Effects of pH and temperature on the catalytic activity of FeN_3_P-SAC. (c) Time-dependent TMB chromogenic curves obtained for different concentrations of FeN_3_P-SAC over time in HAc-NaAc buffer (pH = 3.6) under H_2_O_2_ (0.915 mM) and TMB (0.064 mM). (d) TMB chromogenic curves for FeN_3_P-SAC (55.56 µg mL^−1^) upon the addition of H_2_O_2_ at 600 s, 1200 s, and 1800 s in HAc-NaAc buffer (pH 3.6) under H_2_O_2_ (0.867 mM) and TMB (0.275 mM). The control indicates no addition of samples. (e) Catalytic recyclability of FeN_3_P-SAC (50 µg mL^−1^) in the presence of H_2_O_2_ (1.462 mM) and TMB (0.623 mM). (f) Reaction-time curves of the TMB colorimetric reaction catalyzed by FeN_3_P-SAC (25 µg mL^−1^), Fe_3_O_4_ (25 and 100 µg mL^−1^), and the control under H_2_O_2_ (14.625 mM) and TMB (0.465 mM). The control indicates no addition of samples. (g and h) Characterization of the catalytic kinetics by FeN_3_P-SAC. The initial reaction velocity *v* was measured in HAc-NaAc buffer (pH = 3.6). When the concentration of H_2_O_2_ was varied, the concentration of TMB used for FeN_3_P-SAC was 0.622 × 10^−3^ M. When the concentration of TMB was varied, the concentration of H_2_O_2_ was 0.141 M. (i) Mimicry of natural enzyme active sites and schematic of the therapeutic mechanism of FeN_3_P-SAC in nanocatalytic therapy. (j) TEM image and the corresponding EDX elemental mapping images of FeN_3_P-SAC. The white dashed circles indicate the distribution of Fe element within FeN_3_P-SAC. Scale bars, 200 nm. Reproduced with permission.^148^ Copyright 2025, the Royal Society of Chemistry.

#### 4.2.2 Catalase (CAT)-like activity

CAT-like activity is another critical antioxidant mechanism involving H_2_O_2_ decomposition into water and oxygen. This activity helps alleviate hypoxic conditions in tumor microenvironments, thereby inhibiting cancer progression. Unlike POD-like activity, CAT-like catalysis requires the metal site to divert the reaction pathway from ·OH generation toward O_2_ formation, a distinction that is governed by the binding mode of H_2_O_2_ and the availability of adjacent sites for proton transfer. In a recent study, Cao *et al.* developed a cobalt-based single-atom catalyst (Co/PMCS) that mimics both CAT and SOD activities.^149^ The catalyst effectively eliminated O_2_·^−^, H_2_O_2_, and ·OH, reducing reactive oxygen and nitrogen species (RONS) in septic mice. This treatment reduced pro-inflammatory cytokine levels, protected organs from damage, and improved survival rates.^150^ The dual CAT/SOD functionality of Co/PMCS demonstrates that certain SANs can orchestrate sequential antioxidant reactions through a single metal site, mimicking the cascade defense mechanism of natural antioxidant enzymes. Compared to the single-atom Fe_1_NC variant, Fe_2_NC demonstrated enhanced catalytic performance. When H_2_O_2_ was present, Fe_2_NC could generate ·OH and possessed photothermal conversion ability. These properties allowed Fe_2_NC to achieve synergistic antimicrobial therapy.

#### 4.2.3 Superoxide dismutase (SOD)-like activity

SOD-like nanozymes have emerged as pivotal antioxidant therapeutics that scavenge O_2_·^−^, which is the primary source of endogenous ROS. The efficiency of SOD-like SANs depends on the redox potential of the metal center and its ability to undergo reversible oxidation-state changes with minimal energy penalty. Yan *et al.* designed a Pt/CeO_2_ single-atom nanobandage. This material showed SOD-like activity four times higher than that of CeO_2_ clusters.^79^ This improved catalytic performance allows steady neutralization of ROS. This material can continuously reduce oxidative stress and work well in non-invasive neurotrauma treatment. The enhancement over CeO_2_ clusters underscores a critical design principle: the support material actively participates in redox cycling through metal–support electronic coupling, rather than serving solely as a dispersant for the active sites. The catalytic function of Pt-based SANs can be further strengthened by photonic excitation. Xiang *et al.* developed graphitic carbon nitride-supported Pt SANs (Pt SA/C_3_N_4_), demonstrating dual enzyme-mimetic activity, SOD-like O_2_·^−^ scavenging and CAT-like H_2_O_2_ decomposition.^151^ This system regulated mitochondrial ATP synthesis while removing ROS and reactive nitrogen species (RNS), which effectively slowed the progression of osteoarthritis. The catalyst had high photothermal conversion efficiency and showed tunable activity in response to near-infrared (NIR) light. This created a new therapeutic platform for inflammatory diseases driven by oxidative stress.

#### 4.2.4 Oxidase (OXD)-like activity

OXD-like nanozymes play a key role in SAN systems. These redox-active catalysts utilize molecular oxygen (O_2_) as a substrate, and their reaction specificity is dependent on the properties of hydrogen donors. Nanozymes with moderate reducing activity can catalyze O_2_ reduction to H_2_O. Those with stronger reducing ability can reduce O_2_ to H_2_O_2_.^51^ The OXD-like pathway is intrinsically linked to the adsorption and activation of O_2_ at the metal site, which is governed by the d-band center position and the degree of O–O bond weakening upon adsorption. In practical clinical applications, Qiu *et al.* built a nanocatalytic platform by combining l-arginine-functionalized copper SANs (l-Arg@Cu-SAzymes) with low-dose H_2_O_2_. This study demonstrated the potential of such a system for further preclinical evaluation.^152^ The system operates through sequential catalytic processes, where CAT-like activity decomposes H_2_O_2_ into O_2_, followed by OXD-like activity converting O_2_ to cytotoxic ·O_2_^−^. This dual functionality achieved potent antimicrobial effects while enhancing tissue regeneration through collagen matrix restructuring. Further advancing this concept, Wang *et al.* designed bioinspired Mn-anchored nitrogen-doped hollow carbon spheres (Mn/N-HCN).^153^ Shen *et al.* developed an AOC-Co SAzyme with a transition metal active center for oral cancer treatment. With strong O_2_ adsorption capacity, it effectively weakens the O=O bond in OOH*, demonstrating high OXD-like activity. Moreover, AOC-Co SAzyme enables efficient CAT-like therapy through substrate recycling by decomposing H_2_O_2_-generated O_2_.^154^ These examples illustrate that OXD activity can be amplified by structural features that stabilize O_2_ adsorption intermediates, offering a pathway to enhance therapeutic efficacy in oxygen-rich or H_2_O_2_-supplemented environments.

#### 4.2.5 Multi-enzyme catalytic systems

The integration of multiple enzyme-mimetic activities within a single SAN platform represents an advanced design paradigm that enables synergistic therapeutic effects beyond the sum of individual pathways. Iron single-atom catalysts (FeNC-FA) exhibited quadruple enzyme-mimetic activity (POD, CAT, OXD and GPx), producing highly cytotoxic ·OH and singlet oxygen species (^1^O_2_) while inhibiting up-regulated antioxidants (glutathione) in tumors, enabling combined cancer therapy. According to the findings of Yang *et al.*, a closed-loop catalytic method employing folate-functionalized FeNC-FA was proposed, predicated on the multi-enzyme-mimetic activity of SANs.^155^ By leveraging its POD and OXD enzyme-mimicking capabilities, combined with photothermal therapy (mPTT), FeNC-FA can induce ROS and consume GSH in the specific TME to initiate ferroptosis. Concurrently, the accumulation of ROS effectively cleaves heat shock proteins (HSPs), thereby enhancing mPTT (Fig. 9). The multi-enzyme capability of FeNC-FA demonstrates that SANs can be engineered as autonomous therapeutic systems that simultaneously generate cytotoxic species, deplete protective antioxidants, and sensitize tumors to external stimuli, providing a blueprint for next-generation catalytic nanomedicines.

**Fig. 9:**
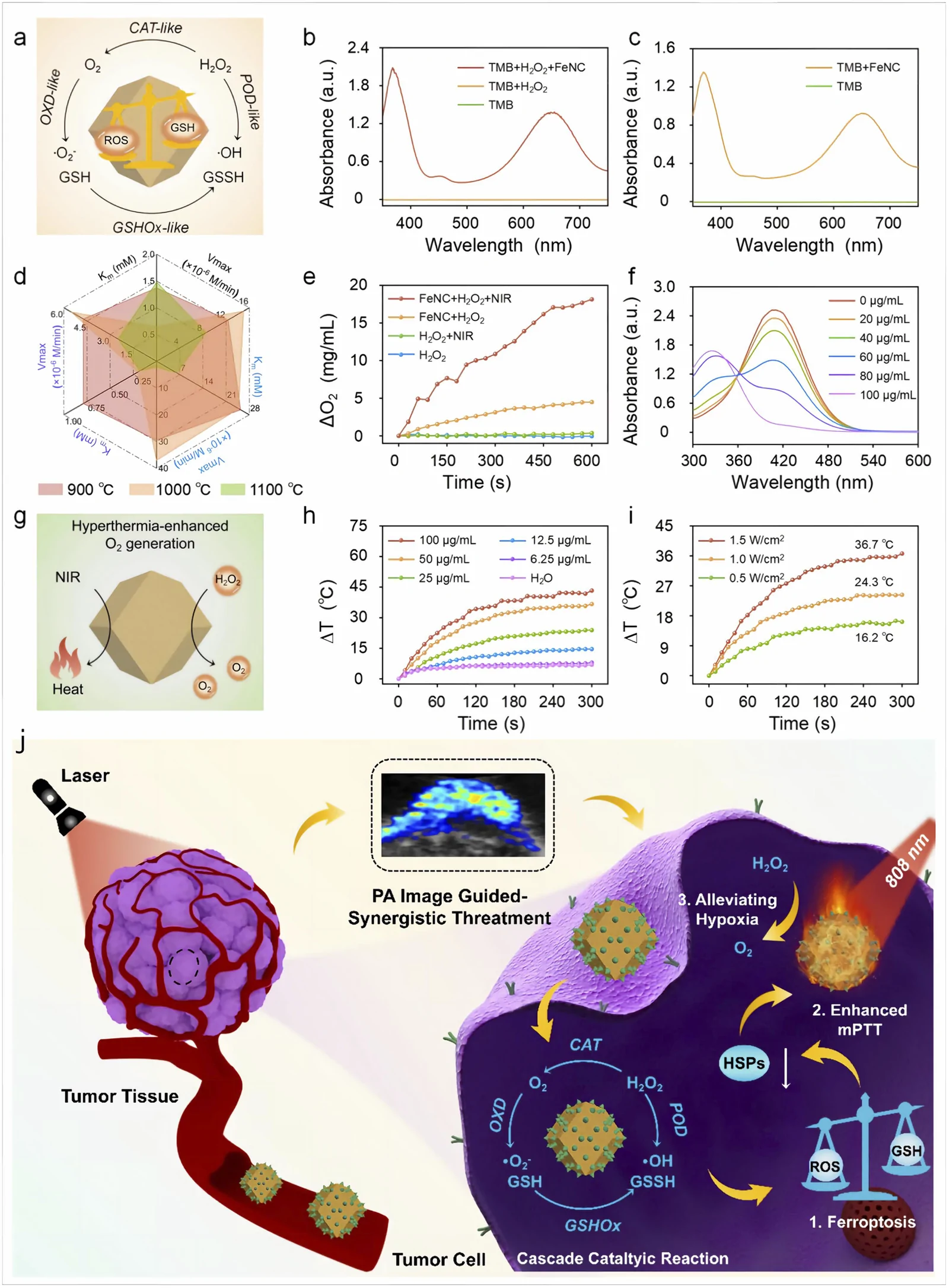
The multienzyme activity and photothermal performance of FeNC. (a) Schematic presentation of multienzyme catalytic activities of FeNC. TMB assay for (b) POD-like and (c) OXD-like activities of FeNC. (d) Michaelis–Menten kinetic analysis of FeNC pyrolyzed at 900, 1000 and 1100 °C, respectively. The black-marked *K*_m_ and *V*_max_ represent POD-like activity with TMB as a substrate. The blue-marked *K*_m_ and *V*_max_ represent POD-like activity with H_2_O_2_ as a substrate. The purple-marked *K*_m_ and *V*_max_ represent OXD-like activity with TMB as a substrate. (e) O_2_ generation of FeNC under different treatments. (f) GSH depletion after treatment with different concentrations of FeNC. (g) Schematic image of hyperthermia-enhanced O_2_ generation. (h) Temperature change of water and FeNC aqueous solutions at different concentrations irradiated with a laser (808 nm, 1 W cm^−2^). (i) Temperature change of the aqueous dispersion (50 µg mL^−1^) under 808 nm laser irradiation at different power densities. (j) The mechanism of PA image guided catalytic-mild temperature photothermal synergistic strategy. Reproduced with permission.^155^ Copyright 2025, Springer Nature.

## 5. Size-dependent structure–activity relationships

Nanozymes can be systematically classified into three principal categories based on their constituent structural characteristics: nanoparticles, nanoclusters, and SANs. This classification system mainly considers the quantitative configuration and structural arrangement of catalytic components. Although these categories bear fundamental interrelationships, metal-containing nanostructures exhibit clear differences in catalytic performance. These differences depend on their composition, even when the elemental composition is exactly the same.

### 5.1 Nanoparticles *versus* single-atom nanozymes

SANs may exhibit higher catalytic activity and reaction selectivity than conventional nanocatalysts owing to their high atomic utilization and well-defined active sites. Their theoretically complete atomic utilization may reduce the required metal dose; however, potential toxicity, metal-ion release, biodistribution, and pharmacokinetics depend on the entire nanoplatform and the stability of the metal–support interaction. The performance advantage of SANs over conventional nanozymes originates from two interconnected factors: the complete exposure of active sites, which eliminates the mass-transport limitations inherent to buried catalytic centers in nanoparticles, and the precisely defined coordination environment, which enables rational modulation of electronic structure for optimized substrate activation. Current research reveals that the catalytic activities of numerous reported nanozymes (including Mn_3_O_4_, MnO_2_, Prussian blue, CeO_2_, metallic nanoparticles, Fe_3_O_4_, Co_3_O_4_, and graphene quantum dots) remain substantially inferior to those of natural enzymes. To solve this problem, Cao *et al.* developed a Co/PMCS single-atom catalyst. It has marked catalytic improvement and cost-effectiveness, and can serve as a good substitute for natural enzyme systems.^149^ The enhanced performance of Co/PMCS exemplifies how atomic dispersion overcomes the inherent inefficiency of nanoparticle catalysts, where only surface atoms contribute to catalysis while interior atoms remain inaccessible.

In related studies, Yan *et al.* found that single-atom Pt-doped CeO_2_ nanostructures had much better catalytic performance than traditional CeO_2_ clusters.^79^ Beyond mere atomic dispersion, the Pt/CeO_2_ system reveals that the support plays an active role in determining catalytic function. The study revealed that single Pt atoms preferentially disperse on CeO_2_ crystal planes, inducing 2% lattice expansion. This structural change exposes more catalytic active sites on the surface. It also forms stable Pt–O–Ce bonds. These bonds prevent the agglomeration of active sites. Furthermore, electronic structure modulation elevates the Ce^3+^/Ce^4+^ ratio, enhancing electron transfer capacity and reducing energy barriers for RONS scavenging and enzymatic reactions. The single-atom configuration also optimizes Pt utilization, creating synergistic effects that enhance both multi-enzyme-mimetic activities and radical scavenging efficiency. When this optimized Pt/CeO_2_ structure is used as a clinical anti-inflammatory agent, it improves RONS scavenging ability by 2 to 10 times. It also raises multi-enzyme mimetic activity by 3 to 10 times. These enhancements underscore a general principle: the electronic coupling between isolated metal atoms and the support can generate emergent catalytic properties that are absent in the corresponding nanoparticle or cluster systems.

The structural uniformity of SANs further enables precise control over the coordination geometry, offering a distinct advantage over the heterogeneous surface of conventional nanozymes. Chang *et al.* engineered atomically dispersed Ce nanozymes (CeN_4_-SANs) with symmetrical Ce–N_4_ coordination.^156^ Unlike the aggregated CeO_2_ nanoparticles with sparse and uneven active sites, CeN_4_-SANs feature fully exposed active centers and structural uniformity. The Ce–N electronic interaction facilitates efficient electron transfer, enabling flexible Ce^3+^/Ce^4+^ redox cycling. This electronic structure raises the adsorption energy of H_2_O_2_ to −2.01 eV. It helps H_2_O_2_ break down into ·OH efficiently. This gives the material strong peroxidase-like activity. The CeN_4_–SAN example demonstrates that the well-defined coordination environment of SANs allows for atomistic-level understanding of structure–activity relationships—a level of mechanistic insight that is challenging to achieve with polydisperse nanoparticle systems. These findings indicate that SANs can exhibit superior catalytic performance relative to conventional nanozymes in specific metal-based systems.

### 5.2 Nanoclusters *versus* single-atom nanozymes

Despite these advances, SANs still encounter practical limitations and may not always achieve the desired catalytic performance. While Pt-based catalysts offer high activity, their clinical translation often necessitates integration with complementary systems. In contrast, Au exhibits weaker intrinsic activity but possesses a broader repertoire of enzyme-mimetic functions. These functions give Au considerable potential value. The catalytic behavior of metal nanostructures is not solely determined by the metal identity; it is equally governed by the nuclearity and atomic arrangement, which dictate the electronic structure and the availability of cooperative active sites. The methods used to make Au materials have always been complex. These methods also need strict and careful spatial coordination. For a long time, these two problems stopped researchers from studying Au at the atomic level. This changed when Liu *et al.* developed new Au-based nanocluster enzymes.^157^ Replacing single Cu or Cd atoms changes the electronic structure in Au_24_Cu_1_ and Au_24_Cd_1_. This change creates more empty spaces in the 5d orbitals of Au atoms. It also greatly improves the electron exchange ability of these atoms. The antioxidant activities of Au_24_Cu_1_ and Au_24_Cd_1_ are 41 times and 48 times that of Au_25_ respectively. S–Cu bonds are short and rigid, while S–Cd bonds are long and flexible. These two different kinds of bonds form different active sites. Au_24_Cu_1_ gets high CAT-like activity from this structure. Au_24_Cd_1_ gets efficient SOD-like performance from it. In contrast, Au_25_ exhibits almost no antioxidant or enzyme-mimetic activity because its fully saturated electronic structure lacks well-defined catalytic centers. The Au_24_Cu_1_ and Au_24_Cd_1_ systems demonstrate that precise control over atomic composition and bonding geometry at the cluster level can direct catalytic selectivity toward distinct enzyme-mimetic pathways, a degree of functional differentiation that is difficult to achieve with either larger nanoparticles or isolated single atoms. These clusterzymes make use of their targeted catalytic functions. They can precisely remove the byproducts of oxidative stress. They can also suppress specific inflammatory factors. Au_24_Cu_1_ can inhibit TNFα. Au_24_Cd_1_ can suppress IL-1β and IL-6. These effects break the vicious cycle of oxidative damage and neuroinflammation. In contrast, Au_25_ fails to elicit any anti-inflammatory effect due to its catalytically inert structure.

The comparison between single atoms and nanoclusters is further illuminated by the distinct mechanistic pathways they promote. Hu *et al.* prepared Se single atoms supported on NC frameworks (Se SA@NC) and employed DFT to compute the adsorption energies of OOH*, O*, and OH* intermediates on N–C, Se SA@NC, and Se clusters. For Se SA@NC, the rate-determining step was the transformation from O* to OH* at *U* = 0 V. The energy barrier Δ*G* of this step was 0.23 eV. For Se clusters, the rate-determining step was the conversion from O_2_ to OOH*. Its energy barrier was 0.70 eV, which was much higher. These results prove that Se single atoms can serve as efficient active sites for the oxygen reduction reaction (ORR). They also show better catalytic performance than Se clustered structures.^158^ This mechanistic analysis reveals that the rate-determining step differs fundamentally between single atoms and clusters, implying that the choice of nuclearity must be matched to the specific reaction coordinate of the target catalytic process.

The principle that smaller nanostructures are generally more active has been widely adopted in nanoscale catalysis. For this reason, the size of metal particles strongly affects catalytic performance. These particles include nanoparticles, nanoclusters and single atoms. However, this design rule does not apply universally: in biological reactions that require cooperation between multiple catalytic sites, SANs show lower catalytic activity than nanoclusters. Bimetallic and polymetallic interactions can improve catalysis. They achieve this by changing active surfaces or controlling catalytic sites. Nanoclusters use atoms more efficiently than nanoparticles. However, they are less stable than single atoms. This shows that these structures have special physical and chemical properties. Non-metal coordinated clusters display hybrid nanoparticles/single-atom characteristics from the coexisting metal–metal and metal–heteroatom bonds.^159^ The catalytic activity of SANs and nanoclusters is influenced by multiple structural factors, including atomic coordination and particle size, and the optimal structure depends on whether the reaction requires isolated active centers or ensemble effects. Liu *et al.* synthesized platinum catalysts spanning single atoms, clusters, and nanoparticles. Photocatalytic H_2_ evolution under UV irradiation revealed that Pt–SANs showed minimal activity compared to Pt clusters and nanoparticles, with Pt clusters exhibiting superior performance.^160^ Similarly, Ji *et al.* investigated ruthenium-based systems and demonstrated that Ru_3_ clusters displayed enhanced catalytic activity and selectivity relative to both Ru_1_ SANs and Ru nanoparticles in phenylacetylene hydrogenation, highlighting the critical role of atomic ensemble effects.^161^ These findings underscore that the catalytic performance of SANs and nanoclusters is governed by multiple structural factors, and a nuanced understanding of these effects is essential for the rational design of nanozymes for specific biomedical reactions. Huang and his research team found that Ru NC/NC catalysts show excellent hydrogen evolution reaction (HER) performance. This good performance comes from the strong electronic interaction between Ru nanoclusters and the nitrogen-doped carbon support. This interaction downshifts the d-band center and promotes favorable hydrogen adsorption on Ru active sites. The catalytic effect of Ru NC/NC is much better than that of Ru SAs/NC and Ru NPs/NC.^162^ Sun *et al.* further compared Ru SANs and Ru clusters in biomedical applications.^163^ Ru clusters showed improved ROS scavenging efficiency and accelerated H_2_O_2_ decomposition kinetics relative to isolated Ru atoms. These traits improve the survival, attachment and differentiation abilities of human mesenchymal stem cells (MSCs). This proves the good therapeutic value of nanoclusters. The better performance of clusters comes from cooperative effects between nearby atoms. It also comes from electronic properties that rely on cluster size. Researchers can control the number of atoms in catalytic groups very accurately. This allows them to adjust catalytic activity for special nanomedicine uses. These uses include ROS control and tissue regeneration.

These comparisons demonstrate that the catalytic advantage of SANs is not universal. Although isolated single atoms offer high atomic efficiency and well-defined coordination environments, nanoclusters may be more effective for reactions requiring cooperative interactions between neighbouring metal sites (Table 3). The optimal nuclearity is therefore reaction-dependent and should be selected according to the required catalytic mechanism rather than the general assumption that smaller structures are inherently superior. This structure–activity relationship provides a rational basis for the classification and design strategies discussed below.

**Table 3:** Representative summary of nanozymes, single-atom nanozymes, nanoclusters and metal nanoparticles^a^

Comparison index	SANs	Nanoclusters	Nanoparticles
Size	Metal sites < 0.2 nm; support particle size typically 20–80 nm ^16,48^	∼0.2–2 nm sub-nano scale^58^	∼2–100 nm (≥2 nm)^58^
Number of metal nuclei	Fully isolated single metal atom^164^	∼2–144 atom clusters^165^	Continuous metal lattice (typically >1000 atoms)^166^
Metal loading (wt%)	∼0.1–2 (commonly ultra-low, *e.g.*, Co/PMCS = 1.2)^99,149^	∼1–5 (commonly medium, *e.g.*, Ru_3_@ZIF-8 = 2.1)^162^	∼5–30 (commonly high, *e.g.*, Ru NPs@ZIF-8 = 8.5)^3,162^
Atomic utilization rate	Theoretical metal-atom utilisation may approach 100% when all isolated sites are accessible; actual catalytic accessibility depends on the support and reaction environment^16,20,48^	∼30–80%; the accessible fraction depends on cluster size, geometry, and the definition used^58^	Generally lower than that of SANs and small clusters in the cited systems; for example, approximately 15% was estimated for particles of approximately 20 nm^58^
Active site homogeneity	Highly homogeneous, identical coordination^48,105,167^	Moderate heterogeneity (*e.g.*, vertex/edge difference)^58^	Highly heterogeneous (mixed crystal facets/defects)^58^
Coordination environment	M–N/M–O/M–C; no metal–metal bonds^16^	M–M core + surface heteroatom bonds^58,158^	Continuous M–M lattice, sparse surface M–O bonds^58^
Structural stability	Generally resistant to oxidation; prone to aggregation under reducing or high-temperature conditions^101,102^	Kinetically stabilized; may gradually ripen into larger particles over long-term use^58,160^	Thermodynamically more stable than clusters; suffer from sintering at high temperature^58^
Enzyme-mimicking activity	Multi-enzyme (POD/CAT/SOD/GPx) synergistic catalysis^149,158^	Multi-site synergy, tunable substrate selectivity^58,159,160^	Activity strongly size/shape-dependent; often lower selectivity; non-specific side reactions possible^58^
Biomedical applications	Reported examples include sepsis treatment, antibacterial therapy, neuroprotection, and antioxidant therapy^168^	Reported examples include anti-inflammatory therapy, ROS scavenging, and cytoprotection^169^	Extensively investigated in both *in vitro* and *in vivo* applications, including imaging, drug delivery, photothermal therapy, and catalytic therapy; biodistribution and tissue accumulation remain system-dependent concerns^170^
Core drawbacks	Potentially low metal loading or active-site density, possible aggregation, demanding atomic-level characterisation, and challenges in reproducible large-scale fabrication^59,61^	High cost and complexity of atomically precise synthesis, structural evolution during use, and potentially slow or variable *in vivo* clearance^171–173^	Lower metal-atom efficiency, heterogeneous active sites, potentially reduced selectivity, and composition- and dose-dependent toxicity or organ accumulation^174,175^

a The quantitative values shown including dimensions, number of metal atoms, metal loadings, and atomic-utilization estimates are representative ranges derived from the specific systems and experimental conditions reported in the cited references. They are not universal limits or defining classification boundaries. Reported values may vary substantially with metal composition, support material, synthesis method, particle-size measurement, surface chemistry, reaction environment, and the definition used for atomic utilization. Accordingly, comparisons among the three material classes should be interpreted qualitatively rather than as absolute performance rankings.

## 6. Engineering strategies to enhance the catalytic performance of SANs

Some SANs can outperform conventional nanozymes or natural enzymes under specific conditions. They exhibit highly precise catalytic activity at the atomic level. They also have strong environmental adaptability, including good tolerance to different pH and temperature conditions. Their structural stability is also substantially higher than that of conventional nanozymes. Even with these clear advantages, researchers continue to explore the multifunctional catalytic properties of SANs by using new and creative engineering methods. This section critically evaluates state-of-the-art strategies for optimizing SANs (Fig. 10), focusing on atomic-scale engineering techniques that bridge fundamental catalysis science with emerging applications in precision nanomedicine.

**Fig. 10:**
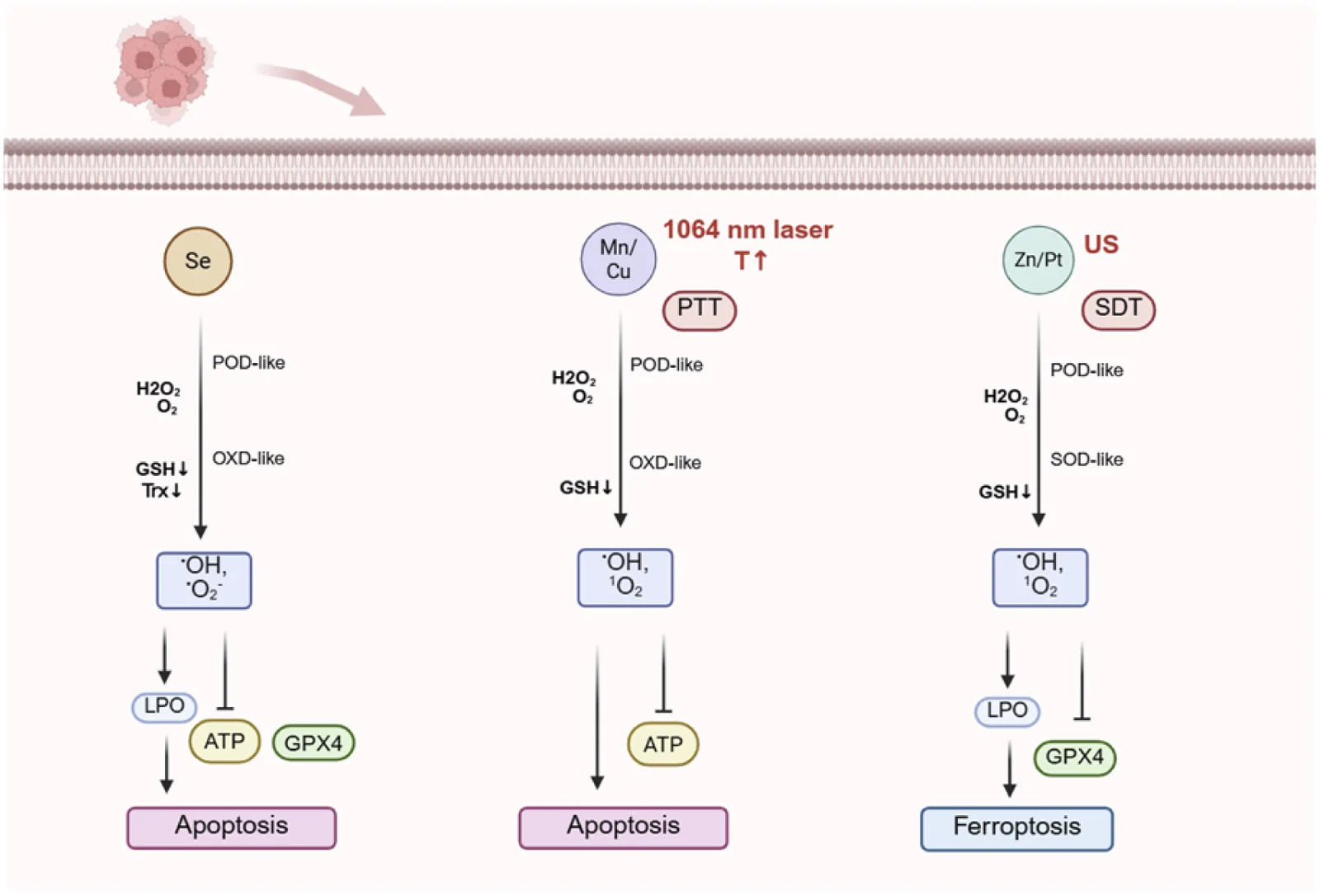
Schematic illustration of three representative strategies for enhancing the catalytic and therapeutic performance of single-atom nanozymes (SANs). Left: Se-based SANs exhibit POD- and OXD-like activities, promoting ROS generation and disruption of intracellular redox homeostasis through glutathione (GSH) and thioredoxin (Trx) depletion, leading to lipid peroxidation (LPO), ATP depletion, GPX4 inhibition, and apoptosis. Middle: near-infrared (1064 nm) laser-induced photothermal therapy (PTT) enhances the POD- and OXD-like activities of Mn/Cu-based SANs, promoting ROS generation and GSH depletion and disrupting cellular energy metabolism, ultimately inducing apoptosis. Right: ultrasound (US)-mediated sonodynamic therapy (SDT) enhances the catalytic activity of Zn/Pt-based SANs, promoting ROS generation and GSH depletion, suppressing GPX4 activity, and enhancing lipid peroxidation, ultimately inducing ferroptosis. The figure was created using https://BioRender.com.

### 6.1 Metal-based SANs

Metal-based SANs are the main focus of current SAN research. Isolated metal atoms are fixed onto solid supports in these catalysts. They have the highest atomic utilization efficiency and clear active sites. The chemical stability of metal atoms keeps the structure stable under working conditions. However, this stability also brings difficulties. It is hard to improve catalytic performance because these metal atoms have natural electronic inertness.

#### 6.1.1 Spatial engineering of single-atom active sites

The catalytic activity of SANs can be tuned by axial coordination methods. These methods use structural adjustments outside the plane to improve the function of metal active sites.^176^ Beyond axial coordination, the spatial positioning of active sites within the host framework has been relatively overlooked, despite being an equally important design parameter. Ou *et al.* addressed this gap. They demonstrated that adjusting the position of Cu single-atom sites on PHI can greatly improve enzymatic activity.^177^ By employing distinct synthesis pathways, two Cu-SAN configurations were achieved: interlayer-localized Cu (Cu L/PHI) and intraplanar-localized Cu (Cu P/PHI). While both structures retained similar macroscopic morphology, detailed analysis revealed critical differences in atomic coordination. In Cu L/PHI, copper atoms connect between PHI layers to form Cu–N_4_ structures, whereas in Cu P/PHI, copper atoms adopt Cu–N_2_ coordination within the PHI planes. The interlayer configuration enabled superior electron transfer between layers, allowing precise control over coordination parameters (*e.g.*, bond length, coordination number) and electronic structure compared to their intraplanar counterparts. This improvement facilitated electron transfer during photocatalysis and activated O_2_ more effectively, enabling efficient formation of ROS (·OH and ·O_2_^−^) under 405 nm LED irradiation. The bactericidal efficacy of Cu L/PHI stems from ROS-induced oxidative damage. ROS act on both Gram-negative (*Escherichia coli*) and Gram-positive methicillin-resistant *Staphylococcus aureus* (MRSA). ROS directly penetrate bacterial membranes, oxidize intracellular contents, and compromise structural integrity, collectively leading to bacterial cell death. Importantly, Cu L/PHI had the most ROS produced in bacterial microenvironments. Its antibacterial effect was comparable to that of traditional antibiotics. The interlayer configuration of Cu L/PHI outperforms its intraplanar counterpart because the spatial arrangement creates an electron transfer pathway between layers that is absent in the planar geometry. This finding establishes that spatial positioning is not merely a structural curiosity but a tunable parameter that can determine catalytic efficiency independent of metal identity or coordination number.

Wang *et al.* applied spatial engineering to construct a dual-site SAzyme, FePc@2D-Cu-N-C, with vertically stacked Fe–N_4_ and Cu–N_4_ sites. Compared to single-atom and conventional bimetallic systems, FePc@2D-Cu-N-C shows enhanced spatial integration and electronic structure modulation.^178^ Local charge polarization and electronic coupling can increase the work function and raise the spin states. These changes help electrons transfer efficiently from the substrate. The vertical Fe–Cu geometry performs better than planar bimetallic structures. It improves structural support, substrate affinity, and electron kinetics. This result provides new ideas for designing nanozymes at the atomic level. This vertical stacking strategy extends the concept of spatial regulation from single-site positioning to the relative arrangement of two distinct metal centers. The enhanced performance arises from proximity-induced electronic coupling between vertically aligned Fe and Cu sites, which facilitates charge transfer and substrate activation more effectively than planar configurations where the two metal centers are spatially separated.

Expanding on spatial regulation strategies, Shi *et al.* developed interlayer-coupled FeN_4_–FeN_4_ diatomic peroxidase nanozymes (IC-DAN) by atomic-scale modulation of dual Fe sites.^150^ IC-DAN retained nanocubic morphology with distinctive concave surface features while exhibiting pH-dependent POD-like activity. Shi *et al.* employed weakly acidic conditions (pH = 4) to mimic the microenvironment of infected wounds. Under these conditions, IC-DAN showed much stronger POD-like activity. It can break down H_2_O_2_ into toxic ·OH radicals. These radicals oxidize bacterial contents and lead to bacterial cell death. Furthermore, IC-DAN displayed remarkable photothermal conversion efficiency under 808 nm NIR irradiation. In synergistic antibacterial experiments (IC-DAN + H_2_O_2_ + NIR), localized hyperthermia from photothermal therapy (PTT) enhanced membrane permeability, facilitating ·OH penetration and amplifying oxidative damage. This combined method uses both enzymatic ROS production and photothermal effects. It achieved strong bacterial inactivation, which shows IC-DAN has the potential to be a multifunctional antibacterial agent. The IC-DAN system demonstrates that spatial engineering can be extended to homometallic diatomic configurations, where the interlayer proximity of two Fe sites creates a cooperative catalytic environment that outperforms isolated single Fe sites. The photothermal enhancement further illustrates that spatial positioning can facilitate multifunctionality by maintaining structural integrity under external stimuli.

Collectively, these studies show that spatial and coordination engineering is very important for SAN design. The spatial configuration of active sites, whether interlayer, intraplanar, or vertically stacked, determines the electron transfer pathways and substrate accessibility, thereby governing catalytic activity and selectivity. Three distinct strategies emerge from these studies: positioning single atoms between layers to create electron transfer channels, vertically stacking heterometallic pairs to enable electronic coupling, and engineering homometallic diatomic proximity for cooperative catalysis. These approaches collectively demonstrate that spatial regulation constitutes a design dimension orthogonal to compositional optimization for enhancing SAN performance in antibacterial applications. Researchers can improve electron transfer, substrate activation and microenvironment response by adjusting atomic-scale structures. This helps create better nanozyme-based treatments for antibiotic-resistant bacteria.

#### 6.1.2 Dual-atom nanozymes

Dual-atom nanozymes (DANs), an emerging subclass of single-atom catalysts, have garnered significant interest due to their unique catalytic mechanisms and enhanced performance.^179^ Unlike single-atom systems, DANs exploit synergistic interactions between paired metal atoms to amplify catalytic activity. These synergistic effects address three inherent limitations of isolated single-atom sites. First, bimetallic coordination structures prevent atomic aggregation. This improves metal loading and the density of active sites.^180^ Second, heteronuclear bimetallic pairs change electronic structures by shifting the d-band center. This speeds up electron transfer and makes substrate adsorption better.^181^ Third, the synergistic effect between dual-metal sites allows coordinated catalysis of multiple reaction steps. It can adapt to complex enzymatic processes and realize efficient cascade reactions.^182,183^ Through these mechanisms, DANs create catalytic networks rather than isolated catalytic events.

For instance, Li *et al.* developed Mo–Cu dual-atom nanozymes (Mo–Cu DAzyme). These nanozymes have multiple enzyme-mimetic abilities, and they outperform conventional Mo single-atom nanozymes.^183^ The Mo–Cu DAzyme integrates POD-like, OXD-like, GSH-depleting, and NADPH oxidase-like activities. The coexistence of Mo–Cu diatomic sites and Cu monoatomic sites enhances the generation of ROS, including ^1^O_2_, O_2_^−^, and ·OH, while simultaneously depleting GSH and NADPH. This dual-action mechanism disrupts tumor redox homeostasis, providing a potent strategy for catalytic cancer therapy. The Mo–Cu system exemplifies cascade catalysis, where ROS production and NADPH consumption are coupled to create a self-amplifying oxidative stress cycle. Similarly, Zhang and his team reported Mn/Fe dual-atom catalysts (Mn/Fe DANs). Fe atoms in these catalysts have POD-like activity, which decomposes H_2_O_2_ into ·OH. At the same time, Mn atoms catalyze the disproportionation of H_2_O_2_ to produce O_2_.^184^ Moreover, the Mn/Fe DANs also have GSH oxidase-like activity. This activity increases ROS production and forms a self-reinforcing therapeutic cycle. The Mn/Fe system illustrates a complementary division of labor between two metals, with Fe and Mn performing distinct catalytic functions that collectively sustain a therapeutic cycle.

Innovative applications of DANs extend to multimodal therapeutic platforms. Zhu *et al.* developed a dual-single-atom FeCo nanozyme supported on nitrogen-doped carbon (NC), featuring Fe single-atom sites, Co single-atom sites, and Fe–Co dual-atom sites.^186^ Compared to Fe-NC and Co-NC, FeCo-NC shows a lower Michaelis constant (*K*_m_) and higher maximum reaction rate (*V*_ma*x*_) with H_2_O_2_ as the substrate, indicating enhanced substrate affinity, faster turnover rate, and superior POD-like activity. FeCo-NC also depletes more GSH over equal time, demonstrating notable GSHOx-like activity. Under combined photothermal and chemotherapeutic treatment, FeCo-NC regulates bacterial genes involved in growth, metabolism, and membrane integrity, effectively eliminating 99.36% of MRSA and 99.76% of *E*. *coli*.

The enhanced kinetics of FeCo-NC originate from electronic modulation induced by the coexistence of Fe–Co dual sites alongside monometallic sites. Wen *et al.* designed Zn–Mn diatomic catalysts synergized with sono-dynamic therapy (SDT).^187^ Under ultrasonic (US) irradiation, Zn and Mn atoms exhibit distinct roles, where Zn enhances electronic conductivity and Mn facilitates efficient electron excitation. This dual-site configuration amplifies ROS generation during SDT and promotes acoustic cavitation, significantly augmenting ferroptosis in tumor cells. The integration of acoustic energy with DANs exemplifies a promising strategy to enhance therapeutic efficacy through physical–chemical synergy. The Zn–Mn system shows that DANs can be designed to couple catalytic activity with external energy conversion, enabling sonodynamic effects that are inaccessible to either metal alone. Ning *et al.* integrated Fe–Cu diatomic nanozymes with photothermal effects.^185^ Under near-infrared irradiation, FeCu-DA demonstrated synergistic treatment enhancement. The Fe–Cu diatomic sites synergistically enhanced POD-like and CAT-like activities, achieving higher catalytic efficiency and rates than Fe SANs. The FeCu-DA showed strong POD-like activity. The specific activity was 948.05 U mg^−1^. This value is nearly three times higher than that of the material containing only Fe single atoms. The FeCu-DA material also has another useful feature. It can work like GSH-OXD. In this role, it helps change GSH into H_2_O_2_. This cascade catalysis sustainably generates ·OH while depleting GSH to reduce ROS resistance (Fig. 11). In FeCu-DA, GSH depletion not only removes an antioxidant barrier but also supplies additional H_2_O_2_ to sustain the POD cycle, accounting for its nearly three-fold enhancement over Fe SANs. Hu *et al.* developed a hybrid catalyst (Co–Se/Co/NC) incorporating both Co single-atom and Co–Se dual-atom sites through innovative integration of non-metallic selenium. The introduced Se atoms effectively regulate charge redistribution around Co active centers. Moreover, the electronic interaction between Co single-atom and Co–Se dual-atom sites adjusts the position of the d-band center. This improves the adsorption and desorption of intermediates and speeds up the kinetics of the oxygen reduction reaction.^188^ The Co–Se system extends DAN design beyond bimetallic combinations by incorporating a non-metal heteroatom to modulate the electronic structure of Co sites.

**Fig. 11:**
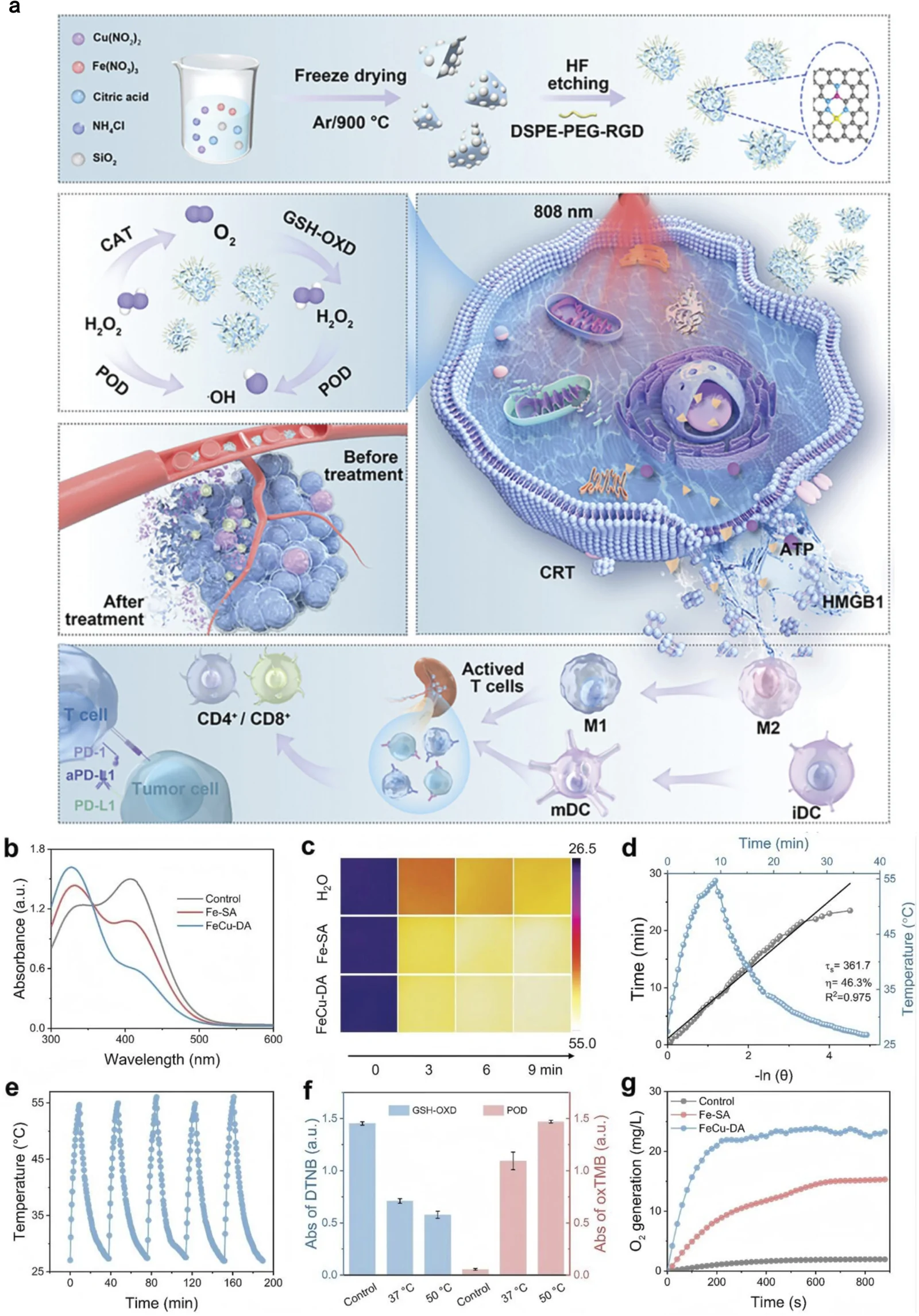
(a) Schematic illustration of the synthetic process and therapeutic mechanism of FeCu-DA. (b) GSH-OXD activity of FeCu-DA and Fe-SA. (c) Thermal images under NIR irradiation. (d) Photothermal conversion efficiency (*η*) of FeCu-DA. (e) Temperature variation over five on/off cycles of FeCu-DA under 808 nm laser irradiation. (f) Comparison of POD and GSH-OXD activities at different temperatures. (g) CAT activity of FeCu-DA and Fe-SA. Reproduced with permission.^185^ Copyright 2024, Wiley-VCH GmbH.

Expanding beyond heterometallic systems, Tian *et al.* engineered homometallic double Fe atom counterparts (Fe_2_NC), where two Fe atoms are bridged by a carbonyl group.^189^ Compared to Fe single-atom counterparts (Fe_1_NC), Fe_2_NC exhibits superior SOD-like activity, efficiently scavenging O_2_^−^ radicals. Additionally, Fe_2_NC shows stronger CAT-like activity, facilitating the decomposition of H_2_O_2_ into O_2_ and suppressing ·OH generation *via* Fenton-like reactions. This dual function allows precise control of oxidative stress. Fe_2_NC also speeds up the oxidation of 3,3′,5,5′-tetramethylbenzidine (TMB). This proves that its OXD-like activity is higher than that of Fe_1_NC. Fe_2_NC represents a distinct category of DANs where two identical Fe atoms are bridged by a carbonyl group. Unlike heterometallic DANs that rely on electronic differences between two elements, this homometallic system leverages proximity between identical metal centers to enable dual functions inaccessible to isolated Fe single atoms.

Collectively, these studies demonstrate that DANs are versatile and can overcome the limitations of single-atom systems. Three design paradigms emerge from these examples. The first is cascade amplification, exemplified by Mo–Cu and FeCu-DA, where sequential reactions are coupled to enhance overall efficacy. The second is heterometallic complementarity, exemplified by Mn/Fe, where two metals perform distinct catalytic functions. The third is physical–chemical synergy, exemplified by Zn–Mn and FeCo–NC, where catalytic activity is integrated with external energy inputs. Additionally, Co–Se and Fe_2_NC demonstrate that DAN design can be extended to non-metal heteroatoms and homometallic configurations. Through the utilization of synergistic interatomic interactions, DANs can precisely control catalytic pathways. This allows them to adjust ROS levels, improve substrate specificity, and achieve multifunctional therapeutic effects. These advances make DANs important tools for next-generation catalytic medicine.

#### 6.1.3 Heteroatom engineering

To tackle synthetic challenges and enhance catalytic efficiency, heteroatom engineering has emerged as a promising strategy.^190,191^ The introduction of heteroatoms into the coordination environment modulates the electronic structure of metal centers by altering charge distribution and coordination symmetry, thereby tuning catalytic activity and selectivity. Tian *et al.* encapsulated Fe dual-atom nanozymes (Fe_2_NC) within a porous selenium-doped metal–organic framework (Se-MOF) shell to form Fe_2_NC@Se nanoparticles.^189^ The Se-MOF layer not only stabilized the Fe_2_NC core but also mimicked GPx activity, leading to improved cytocompatibility compared to unmodified Fe_2_NC. This approach demonstrates that heteroatom incorporation can serve dual functions by stabilizing the active core while contributing additional enzyme-mimetic activity through the host framework. Zhou *et al.* incorporated S into a diatomic iron nanozyme (Fe_2_NC) to form Fe_2_SNC. Heteroatom doping with S modulates the electronic structure of both single- and diatomic metal centers, thereby boosting catalytic performance. Their findings show that Fe–Fe diatomic catalysts outperform Fe single-atom systems. The Fe–Fe diatomic catalyst supported on S-doped graphitic carbon nitride exhibits superior oxidative capacity. It can effectively activate PMS and promote electron transfer mediated by the catalyst. This process damages viral genomes and proteins, and damages viral proteins and genomes and impairs viral internalization into host cells. It also significantly improves disinfection efficacy.^192^ The S-doped system illustrates that heteroatoms with different electronegativity from nitrogen can fine-tune the electronic properties of adjacent metal sites, with sulfur doping specifically enhancing the oxidative capacity of Fe–Fe diatomic configurations. Similarly, Jiao *et al.* designed boron-doped Fe–N–C SANs (FeBNC SANs), where boron incorporation facilitated electronic structure rearrangement at the Fe active site.^193^ Theoretical calculations showed that charge transfer caused by boron changed the positive charge of Fe atoms. This change lowered the energy barrier for the formation of hydroxyl radicals. It also made the POD-like activity of the material stronger. These related changes greatly improved the catalytic performance of the artificial enzyme. Boron doping, with its electron-deficient nature, induces charge redistribution that reduces the activation barrier for ·OH generation, highlighting that the choice of heteroatom must be matched to the desired catalytic pathway. Niu *et al.* constructed a Cu-NS single-atom nanozyme (Cu-NS@UK@POx) by innovatively substituting nitrogen with weakly electronegative sulfur, breaking the inherent spatial symmetry of Cu–N_4_ electronic distribution. The Cu-NS SANs possess a high specific surface area (817.77 m^2^ g^−1^) and mesoporous structure that facilitate both exposure of Cu single-atom sites and efficient drug loading. Unlike symmetric Cu–N_4_ configurations, S-doping creates Cu–N_3_S_1_ active sites with enhanced electron transfer capability, optimizing the free energy landscape of catalytic steps. This modification lowers the activation energy of the rate-determining step, accelerating reaction kinetics and strengthening oxidase-like activity. The resulting elevated ROS production effectively induces pyroptosis.^194^ The Cu–NS system demonstrates that heteroatom substitution can break symmetry in the coordination environment, creating asymmetric active sites with distinct electronic properties that are inaccessible to conventional symmetric M–N_4_ configurations.

To address system instability, Wang *et al.* synthesized four noble metal–porphyrin complexes (MxP, *x* = Ir, Ru, Pt, Pd) mimicking natural peroxidase active sites.^195^ Beyond heteroatom-doped carbon supports, molecular catalysts with well-defined coordination structures offer an alternative strategy for enhancing SAN stability and activity. MIrP has higher catalytic activity than horseradish peroxidase (HRP). This superior activity originates from the Ir–porphyrin site's strong H_2_O_2_ adsorption capacity, unique rate-determining step, and low energy barrier, which collectively enhance the material's POD-like activity. Moreover, MIrP maintains exceptional chemical stability under both ambient temperature and high H_2_O_2_ conditions, providing an innovative strategy for enhancing the catalytic performance and stability of SANs. The porphyrin-based system shows that precise molecular design can achieve catalytic efficiency exceeding that of natural enzymes while maintaining robust stability, offering a complementary approach to support-based heteroatom engineering.

### 6.2 Non-metal single-atom nanozymes

Current research on SANs remains predominantly focused on metal-based systems.^196^ The synthesis of non-metallic single-atom catalysts is a complex process, which is more challenging to design than their metallic counterparts. However, there are significant safety concerns associated with the use of conventional metallic SANs. These catalysts undergo solubilization within proteins and organic matter *in vivo*, leading to the accumulation of cellular metal ions and complex biophysicochemical interactions. These interactions ultimately result in acute inflammation and cell death. The development of non-metal SANs addresses this safety concern by utilizing main-group elements that lack the redox versatility of transition metals but can achieve catalytic activity through polarized heteroatom–carbon bonds. It is therefore imperative to explore the development of non-metallic single-atom catalysts for applications in nanomedicine as an alternative to metallic nanozymes, despite the significant challenges involved.

Cheng *et al.* recently achieved a breakthrough by constructing selenium SANs (SeSANs) through precise anchoring in nitrogen-doped carbon (Se_1_-NC) matrices.^197^ These nanozymes exhibit POD-like, glutathione oxidase (GSHOx)-like, and NADPH OXD-like activities. Control experiments demonstrated that this enzymatic activity originated exclusively from atomically dispersed selenium sites. Researchers prepared polyethylene glycol-functionalized SeSAEs (PSeSAEs) to improve stability. These PSeSAEs maintained stable catalytic activity over a wide range of pH values, organic solvent concentrations and temperatures. *In vivo* tests in mouse models confirmed that SeSAEs can be used as antitumor agents. They showed marked treatment effects and very low systemic toxicity. These materials may offer a safer alternative than metal-based nanozymes. The SeSAN system demonstrates that non-metal single atoms can mimic multiple enzyme activities through polarized C–Se–C bonds, which create localized positive charges that facilitate electron transfer without the metal-associated toxicity of conventional nanozymes.

Wang *et al.* aimed to systematically compare the active sites and reaction mechanisms between non-metallic and metallic single-atom catalysts. They developed an electrochemically active multilayer porous carbon membrane. This membrane holds atomically dispersed selenium, and is denoted as SeNCM. The pronounced outer orbital overlap between Se and carbon atoms induces positive charge localization on Se sites. Incorporation of larger-radius Se atoms expands graphite interlayer spacing, generating structural strain and edge defects that enhance mass transport within the carbon framework. The Se sites in SeNCM carry positive charges. These sites work with enlarged interlayer channels and stable electrochemical properties. This total effect makes SeNCM show higher catalytic activity than the commercial Pt/C catalyst.^198^ The SeNCM system reveals that the catalytic mechanism of non-metal SANs differs fundamentally from that of metal-based systems: rather than relying on reversible redox cycling of the metal center, the activity originates from charge polarization at the Se–C interface, which is further amplified by structural strain and defect engineering. Hu *et al.* synthesized a selenium single-atom catalyst (Se SAs@NC) on a nanocage framework for the comparison of non-metallic and metallic SACs. DFT calculations indicated that C–Se–C bonds serve as effective catalytic sites, significantly reducing the ORR free-energy barrier. Se@NC demonstrated superior electrocatalytic performance to commercial Pt/C, with a higher onset potential (0.95 V), half-wave potential (0.85 V), and reduction current density (5.53 mA cm^−2^). After 10 h, Se@NC retained 96% activity *versus* 78% for Pt/C, surpassing most reported nanocatalysts and benchmark noble-metal catalysts.^158^ The Se SAs@NC system further validates the C–Se–C bond as the active motif for non-metal SANs, with the DFT calculations providing mechanistic insight into how the electronic structure of main-group elements can be harnessed for efficient catalysis.

These studies together show the potential of non-metal single-atom systems and heteroatom-modified hybrid materials. They can address the limitations of traditional metal-based nanozymes. Two complementary strategies emerge from these examples. The first is the design of non-metal SANs, exemplified by SeSANs and Se SAs@NC, where isolated main-group atoms serve as catalytic centers through polarized heteroatom-carbon bonds. The second is the engineering of heteroatom-modified carbon frameworks, exemplified by SeNCM, where structural strain and edge defects induced by heteroatom incorporation enhance mass transport and catalytic activity. Both approaches achieve a balance between biocompatibility and catalytic efficiency, offering safer alternatives to metal-based nanozymes. This progress supports safer and more effective therapeutic applications in nanomedicine.

### 6.3 External stimuli-enhanced catalysis

The catalytic performance of SANs depends on both their intrinsic features and external conditions. Certain metal atoms possess unique physicochemical traits that enable them to exceed their intrinsic catalytic activity when external stimuli such as electric fields, light, or ultrasound are applied. Building on established principles from chemical catalysis, a growing number of studies are now expanding the biomedical applications of these materials through external energy input.

Zhong *et al.* demonstrated this concept by using ruthenium-based systems. In these systems, lattice swelling in Ru nanocrystals made nanozymes respond better to self-powered electric fields.^199^ The optimized Ru_1000_ catalyst reached its highest catalytic activity under electrical stimulation. This stimulation used human self-propelled friction devices and was set at 50 V and 3 Hz. It greatly improved the treatment results in mouse breast cancer models. The Ru system shows that electrical stimulation can modulate catalytic activity by inducing lattice strain, providing a means for on-demand activation without the need for exogenous chemical triggers.

Wang *et al.* developed a novel iridium-based single-atom nanocatalyst (Ir/CN SAN) anchored on nitrogen-doped carbon.^200^ When near-infrared irradiation (NIR-II, 1270 nm) is applied, Ir/CN SAN turns light energy into heat and catalyzes the generation of ROS. This combined photothermal and catalytic effect removes bacteria effectively *in vitro*. It also speeds up wound healing and improves lung tissue repair in MRSA-infected models. Wang's team further showed environmental modulation with Mn/N–HCN single-atom catalysts.^153^ These systems combined broad-spectrum NIR-II photoacoustic imaging capability with enhanced enzyme-mimetic activity, enabling synergistic tumor cell apoptosis through combined photothermal therapy and nanocatalytic reactions. The Ir/CN and Mn/N–HCN systems demonstrate that NIR-II irradiation can simultaneously activate catalytic ROS generation and photothermal effects, with the Mn system additionally incorporating photoacoustic imaging for theranostic applications. Complementing these approaches, Li *et al.* developed Pt SANs supported on N-doped porous carbon.^201^ The strong NIR absorption (808 nm laser) of the material permitted precise temperature-controlled sterilization through short-term photothermal activation, achieving effective antimicrobial action without inducing significant organ toxicity. The Pt system extends the photothermal strategy to NIR-I wavelengths, enabling precise temperature control for sterilization while maintaining a favorable biosafety profile. Yin *et al.* coordinated Mn atoms into C_3_N_4_ nanosheet cavities *via* photodynamic therapy, forming C_3_N_4_–Mn SAN.^202^ Under 660 nm irradiation, C_3_N_4_–Mn SAN splits water to generate ·OH, thereby enabling oxygen-independent photodynamic cancer treatment. Resultant lipid peroxidation effectively kills cancer cells, demonstrating significant potential for photothermal therapy-based biomedical applications (Fig. 12). The C_3_N_4_–Mn system provides a distinct mechanism from the previous examples: rather than photothermal conversion, it utilizes visible light to drive water splitting for ·OH generation, offering an oxygen-independent pathway that is particularly relevant for hypoxic tumor environments.

**Fig. 12:**
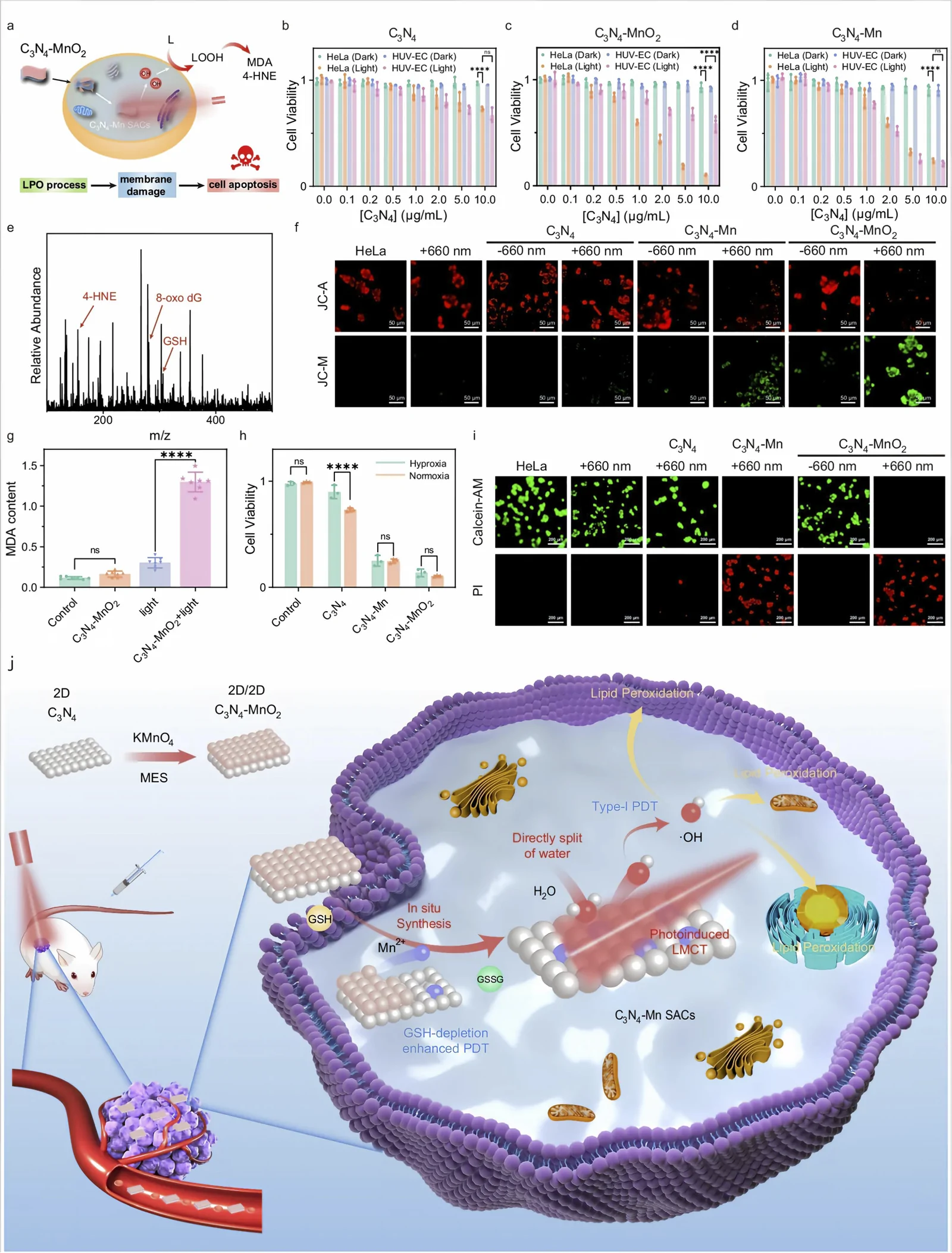
Investigation of the intracellular LPO process by *in situ* synthesized C_3_N_4_–Mn SACs in cancer cells. (a) Scheme of ·OH generation and LPO process by *in situ* synthesized C_3_N_4_–Mn SACs. (b) MTT assay of HeLa and HUV-EC cells incubated with C_3_N_4_. (c) MTT assay of HeLa and HUV-EC cells incubated with C_3_N_4_–MnO_2_ precursors. (d) MTT assay of HeLa and HUV-EC cells incubated with C_3_N_4_–Mn SACs. (e) MS spectrum of HeLa cells after PDT by the *in situ* synthesized C_3_N_4_–Mn SACs for 10 min. The experiment was repeated three times independently with similar results. (f) CLSM images of HeLa cells after different treatments. The cells were stained with JC^−1^. (g) MDA content of HeLa cells after PDT for 10 min. (h) Cell viability of HeLa cells treated with C_3_N_4_, C_3_N_4_–Mn, and C_3_N_4_–MnO_2_ under light irradiation in hypoxia or normoxia environments. (i) CLSM images of the HeLa cells with different treatments. The cells were stained with Calcein-AM and propidium iodide (PI). The experiment was repeated three times independently with similar results. (j) Using PDT therapy, investigation of the intracellular LPO process by *in situ* synthesized C_3_N_4_–Mn SANs in cancer cells. Data are presented as mean ± SD. [C_3_N_4_] = 10 µg mL^−1^. Light irradiation: 660 nm, 0.4 W cm^−2^, 10 min. Reproduced with permission.^202^ Copyright 2024, Springer Nature.

Zhu *et al.* developed a Ru single-atom nanozyme-enhanced titanium dioxide-based sonosensitizer (Ru/TiO_2−*x*_ SAE) with abundant oxygen vacancies and high electron–hole separation efficiency.^203^ This design stabilized Ru single atoms while enhancing oxygen adsorption and accelerating reaction kinetics. Under ultrasound irradiation, the system initiated sonodynamic therapy-amplified ferroptosis by suppressing electron–hole recombination and overcoming the tumor microenvironment's hypoxic and reductive state. This process simultaneously downregulated GPX4 expression through consumption of overproduced glutathione, thus triggering ferroptosis. Density functional theory calculations and experimental results confirmed that the oxygen vacancy-rich Ru/TiO_2−*x*_ SAE exhibited superior catalytic mechanisms and therapeutic efficacy compared to conventional TiO_2_-based sonosensitizers. The Ru/TiO_2−*x*_ system introduces ultrasound as an external stimulus, where oxygen vacancies and Ru single atoms synergistically enhance electron–hole separation and amplify sonodynamic therapy efficacy. Zhou *et al.* synthesized a Cu single-atom-modified WO_3−*x*_ nanocomposite (Cu–WO_3−*x*_) for sonodynamic therapy. Under ultrasound irradiation, its ROS production markedly exceeded that of WO_3−*x*_, Cu–SA-modified WO_3_, and other M–WO_3−*x*_ nanosheets. This enhancement stems from the synergy between copper single atoms and oxygen vacancies in WO_3−*x*_. Cu–WO_3−*x*_ activated both apoptotic and TNF signaling pathways during SDT, effectively eliminating cancer cells and suppressing tumor growth.^174^ The Cu-WO_3−*x*_ system further validates the ultrasound strategy while demonstrating that the choice of metal center and support material can be tailored to achieve distinct therapeutic outcomes, with Cu specifically activating apoptotic and TNF pathways.

## 7. Biosafety assessment

The toxicity of metallic nanozymes can arise from mechanisms including DNA interactions, ROS production, and metal-ion release.^204,205^ Compared with conventional nanozymes, SANs may offer biosafety benefits because their atomically dispersed active sites enable near-complete atomic utilization, thereby reducing the metal loading required for therapeutic efficacy and lowering the risks of metal-ion release and systemic accumulation.^127^ In contrast, conventional nanozymes may require higher material loadings to achieve comparable catalytic activity because only a fraction of their metal atoms serve as accessible active sites.^206^ This may increase the risk of metal-ion release and bioaccumulation, potentially contributing to adverse biological responses. Additionally, the size-dependent clearance behavior of nanomaterials may contribute to the potential biosafety benefits of SANs. The *in vivo* distribution and clearance of nanomedicines are influenced by their size, surface properties, and administered dose, which can substantially affect their biosafety profiles. Larger nanomaterials are commonly cleared through hepatosplenic pathways, whereas sufficiently small nanomaterials may undergo renal clearance, suggesting size-dependent clearance behavior *in vivo*.^207^ However, systematic studies on the long-term biodistribution and clearance of SANs remain limited. Nevertheless, under the conditions evaluated in individual studies, some SANs have demonstrated high catalytic activity together with favorable biosafety profiles and acceptable biocompatibility.^208^

### 7.1 *In vitro* and *in vivo* biosafety evaluations

The biocompatibility of SANs is commonly evaluated through MTT or CCK-8 assays and hemolysis tests. Qin *et al.* demonstrated that the synthesized Fe SANs induced no significant cytotoxicity in either tumor (SMMC-7721) or normal (NIH-3T3) cell lines, even at concentrations up to 1000 ppm in MTT assays, indicating favorable *in vitro* biocompatibility. Selective tumor cell destruction occurred only upon synergistic application of NIR light and H_2_O_2_. Fe atoms are fixed inside Fe–N_4_ coordination structures. These atoms did not release any detectable Fe^2+^ or Fe^3+^ ions after 72 hours of incubation under weakly acidic conditions (pH = 5.0). This finding indicates minimal ion leakage under the tested conditions, suggesting that concerns regarding metal-related biological toxicity may be mitigated for this specific system. *In vivo* studies confirmed that Fe SANs showed hepatic and renal distribution/clearance and preferential accumulation at tumor sites. Quantitative ICP-AES analysis of silicon distribution revealed only 13.8% residual Si after 24 hours, suggesting reduced systemic accumulation over this time frame (Fig. 13).^209^ However, long-term retention and chronic toxicity remain to be evaluated. The Fe SAN system illustrates that strong metal–nitrogen coordination may stabilize the metal atoms and limit ion leakage even under weakly acidic conditions that mimic the tumor microenvironment. Li *et al.* developed ZIF-8@GOx-Pt by employing GOx as a protein scaffold for Pt coordination, overcoming the biocompatibility limitations of conventional inorganic carriers. The coordination-based dispersion of Pt atoms is designed to suppress metal ion leakage, a common concern with conventional Pt-based agents. At equivalent concentrations, Pt SANs showed significant cytotoxicity against HCCLM3 cancer cells while maintaining minimal impact on L929 normal cells. Throughout the murine treatment period, body weight remained stable without notable fluctuations. Comprehensive biochemical analysis, including liver and kidney function markers (ALT, AST, UREA, CR), protein metabolism parameters (TP, ALB, GLB), and other relevant indices, revealed all values within normal ranges. Furthermore, hematoxylin and eosin (H&E) staining of major organs (heart, liver, spleen, lungs, kidneys) revealed no observable pathological abnormalities such as inflammation or necrosis under the evaluated conditions.^210^ The protein scaffold approach used in the ZIF-8@GOx-Pt system provides an alternative strategy for potentially improving biocompatibility, where the biological carrier itself contributes to biocompatibility while the coordinated Pt atoms maintain catalytic activity.

**Fig. 13:**
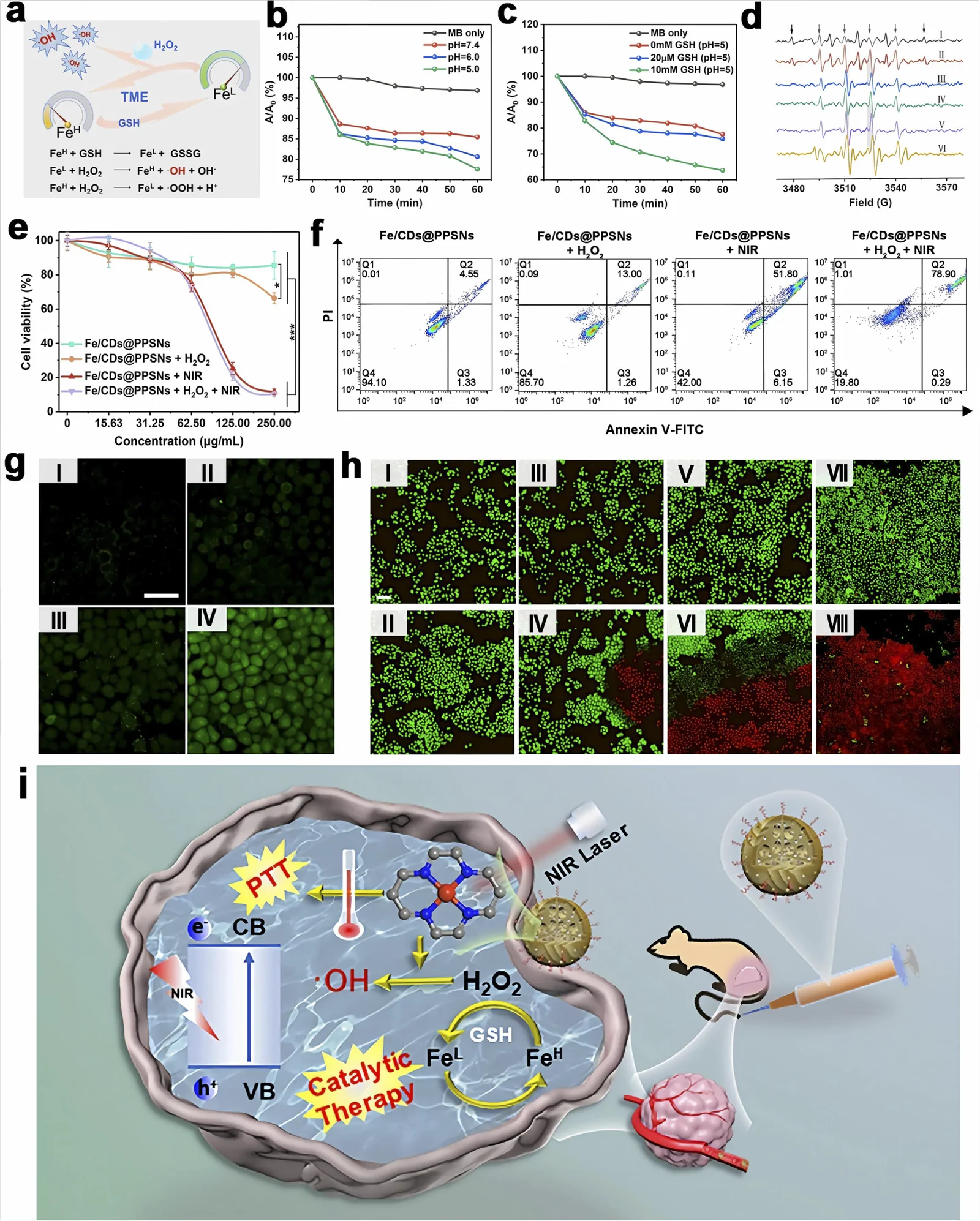
(a) Schematic diagram of tumor catalytic therapy of Fe/CDs@PPSNs. (b) and (c) MB degradation in PBS (10 mM) at various pH values and GSH concentrations, respectively. (d) 5,5-dimethyl-1-pyrroline N-oxide (DMPO) spin-trapping ESR spectra of Fe/CDs@PPSNs under different conditions (I: Fe/CDs@PPSNs (pH = 7.4), II: Fe/CDs@PPSNs + 100 µM H_2_O_2_ (pH = 7.4), III: Fe/CDs@PPSNs + 100 µM H_2_O_2_ (pH = 6.0), IV: Fe/CDs@PPSNs + 100 µM H_2_O_2_ (pH = 5.0), V: Fe/CDs@PPSNs + 100 µM H_2_O_2_ + 20 µM GSH (pH = 5.0), VI: Fe/CDs@PPSNs + 100 µM H_2_O_2_ + 10 mM GSH (pH = 5.0). (e) Cell viability by MTT assay and (f) apoptotic results by flow cytometry analyses of SMMC-7721 cells treated with Fe/CDs@PPSNs. (g) Detection of oxidative stress in SMMC-7721 cells after different treatments: (I) control, (II) H_2_O_2_, (III) Fe/CDs@PPSNs, (IV) Fe/CDs@PPSNs + H_2_O_2_ (scale bar = 50 µm). (h) Staining of living/dead SMMC-7721 cells by different treatments: (I) control group, (II) laser irradiation only, (III) CDs@PPSNs, (IV) CDs@PPSNs + NIR, (V) Fe/CDs@PPSNs, (VI) Fe/CDs@PPSNs + NIR, (VII) Fe/CDs@PPSNs + 100 µM H_2_O_2_, (VIII) Fe/CDs@PPSNs + 100 µM H_2_O_2_ + NIR, all groups were stained with calcein AM and PI, where green fluorescence from calcein AM and red fluorescence from PI indicate living and dead cells, respectively (scale bar = 50 µm). (i) Schematic representation of the mechanism of action of Fe/CDs@PPSNs. Reproduced with permission.^209^ Copyright 2022, Springer Nature.

The MTT assay has become a widely adopted method for evaluating the cytotoxicity and biocompatibility of biomaterials. In Zhu's study, the MTT method was employed to assess Cu–N–C toxicity toward HT29 cells. After 12 h incubation at 0.5 mg mL^−1^, cell viability remained at 77.8%, indicating relatively low cytotoxicity under these conditions. Hemolysis tests were conducted at the same time. The results showed that the hemolysis rate of rat red blood cells remained below 4% even when the concentration of Cu–N–C was 300 µg mL^−1^, comparable to that of the PBS control group. These results suggest that Cu–N–C exhibits favorable hemocompatibility *in vitro*.^211^ The Cu–N–C system demonstrates that copper-based SANs, despite using a metal often associated with cytotoxicity in the nanoparticle form, can achieve favorable biocompatibility through atomic dispersion and carbon matrix encapsulation. Wang *et al.* synthesized Cu SASs/NPCs with a uniform size distribution (193 ± 19 nm, PDI ≈ 0.32), demonstrating good dispersion stability, which may reduce the risk of aggregation-associated toxicity. The material maintained structural integrity and catalytic performance over five laser ON/OFF cycles, indicating good stability under the tested conditions. At concentrations up to 100 µg mL^−1^, minimal hemolysis was observed. MTT assays further revealed low cytotoxicity toward human umbilical vein endothelial cells (HUVECs), suggesting favorable biocompatibility toward this cell type under the tested conditions.^212^ The Cu SASs/NPC system further emphasizes that particle size uniformity and dispersion stability may influence biosafety, as aggregation can induce local toxicity even when the intrinsic material properties are benign.

Overall, SANs may offer potential biosafety advantages over conventional nanozymes under certain conditions, which may arise from their atomically precise architecture and strong metal–support interactions that can mitigate metal ion leakage. Their high atomic utilization efficiency may enable therapeutic efficacy at lower metal loadings and thereby potentially reduce systemic metal burden, while stimuli-responsive activation may further contribute to their biosafety. Nevertheless, the clinical translation of SANs still faces challenges, including insufficient long-term safety validation and the absence of unified evaluation criteria. Incorporating biosafety considerations into the initial material design based on the structure–safety relationship summarized herein may facilitate the development of safer SAN-based nanomedicines for diverse disease therapies.

### 7.2 Mechanisms of SANs' toxicity and mitigation strategies

While SANs may offer potential safety advantages over conventional nanoparticles under certain conditions, they are not devoid of toxicity. Understanding the mechanistic pathways through which SANs may exert adverse effects is essential for their safe design.

Although SANs are deployed to generate ROS for therapeutic purposes, off-target ROS production in healthy tissues can lead to oxidative damage to lipids, proteins, and DNA.^213^ This risk may be particularly relevant for SANs with strong Fenton-like or POD-like activities. The key to mitigating this toxicity lies in exploiting the TME. SANs can be designed to exhibit pH-dependent or H_2_O_2_-dependent activity, exhibiting relatively low activity under physiological conditions (pH 7.4, low H_2_O_2_) while showing enhanced activity in the acidic, H_2_O_2_-rich TME.^214,215^ For example, a triple-enzyme-mimetic PtN_4_C single-atom nanozyme (PtN_4_C-SAzyme) was reported to generate elevated ROS predominantly under TME-like conditions with weak acidity, high H_2_O_2_ and abundant GSH *via* reversible Pt^4+^/Pt^2+^ valence cycling; it showed limited catalytic activity under neutral, normal-tissue-like conditions with limited substrates and avoided superfluous ·OH production. At 100 µg mL^−1^, this nanozyme induced minimal ROS accumulation and kept over 91% viability of normal HC11 mammary epithelial cells but induced severe ROS-mediated death in 4T1 tumor cells. *In vivo* ROS imaging revealed that ROS overproduction occurred predominantly in tumor tissues, without observable oxidative elevation in the heart, liver, spleen, lung, or kidney under the tested conditions. When combined with X-ray irradiation, it predominantly boosted radiation-induced ROS inside tumors, distinct from cisplatin that releases Pt^2+^ systemically and causes widespread organ oxidative injury.^216^

Leakage of redox-active metal ions, particularly Fe and Cu, may promote Fenton or Fenton-like reactions in healthy tissues, potentially leading to ROS accumulation and disruption of cellular redox homeostasis. Specifically, excessive accumulation of iron and copper ions may contribute to ferroptosis and cuproptosis, respectively. Robust M–N_*x*_ coordination represents one of the effective strategies for limiting metal-ion leakage. Beyond interfacial coordination modulation, surface passivation with biocompatible shells (*e.g.*, SiO_2_, polydopamine, and lipid bilayers) can further construct a physical barrier against ion release. A representative design is bioorthogonally modified Cu single-atom nanozyme CuSACO, which features tightly bound planar Cu–C_3_ coordination to restrict spontaneous Cu ion efflux, together with DSPE-PEG lipid coating for extra surface shielding.^217^ Cu ion release was observed to occur gradually under TME-specific high GSH and acidic conditions, with merely 1.84% Cu detected after 24 h incubation in tumor-mimicking buffer; minimal Cu leakage and limited off-target ROS generation were observed under neutral, low-GSH physiological conditions in this study. Benefiting from limited ion escape in healthy organs, CuSACO causes no observable systemic oxidative damage to major visceral tissues *in vivo* under the evaluated conditions, while the GSH-triggered Cu release inside tumors selectively provokes cuproptosis alongside substantial ROS production driven by its triple POD/CAT/OXD-like catalytic activities.

The immune response to nanomaterials is largely dictated by surface characteristics such as charge, hydrophobicity, and protein corona composition, which can trigger complement activation and inflammatory cascades that may culminate in hypersensitivity. PEGylation remains the most established strategy to reduce immunogenicity: it creates a hydrophilic stealth layer that effectively attenuates opsonization and macrophage clearance.^218,219^

Beyond surface chemistry, the long-term fate of the carrier is equally critical. Non-degradable supports, including some carbon-based and MOF systems, may exhibit prolonged retention in organs such as the liver, spleen, and lungs, potentially increasing the risk of chronic inflammatory responses. Therefore, the choice of support material is as important as the selection of the metal center. Accordingly, appropriately designed biodegradable or bioresorbable matrices, including certain mesoporous silica, polydopamine, and ZIF-based systems, may offer advantages by facilitating material degradation and/or clearance.^220^

## 8. Nanomedicine applications of SANs

SANs have emerged as transformative agents in nanomedicine by mediating diverse catalytic reactions with atomic precision.^53,221^ This review systematically examines the functional versatility and mechanistic principles of custom-engineered SANs across four key biomedical domains, namely antimicrobial therapy, oncology, biosensing, and oxidative stress cytoprotection. SANs have adjustable coordination environments and their atomic utilization can reach more than 95%. The ability of single-atom nanozymes to mediate targeted therapy while minimizing off-target effects constitutes a pivotal advantage for their clinical translation.

### 8.1 Antibacterial therapy

Conventional metal-based antibacterial agents are often plagued by metal ion leakage, which raises toxicity concerns and hampers clinical translation. SANs, with atomically dispersed metal sites, minimize such leakage while maintaining potent bactericidal activity and favorable biocompatibility under evaluated conditions.^222^ SANs exert bactericidal effects through two primary pathways: Fenton-like reactions that generate ·OH radicals, and non-radical oxidative mechanisms mediated by species such as ^1^O_2_. Moreover, their unsaturated coordination structures can enhance POD-like activity, enabling the decomposition of H_2_O_2_ into highly oxidative ·OH radicals for effective antimicrobial activity (Fig. 14). This combination of antibiotic-free bactericidal activity, broad-spectrum potential, and potentially reduced risk of resistance highlights SANs as promising platforms for next-generation antimicrobial strategies (Table 4).

**Fig. 14:**
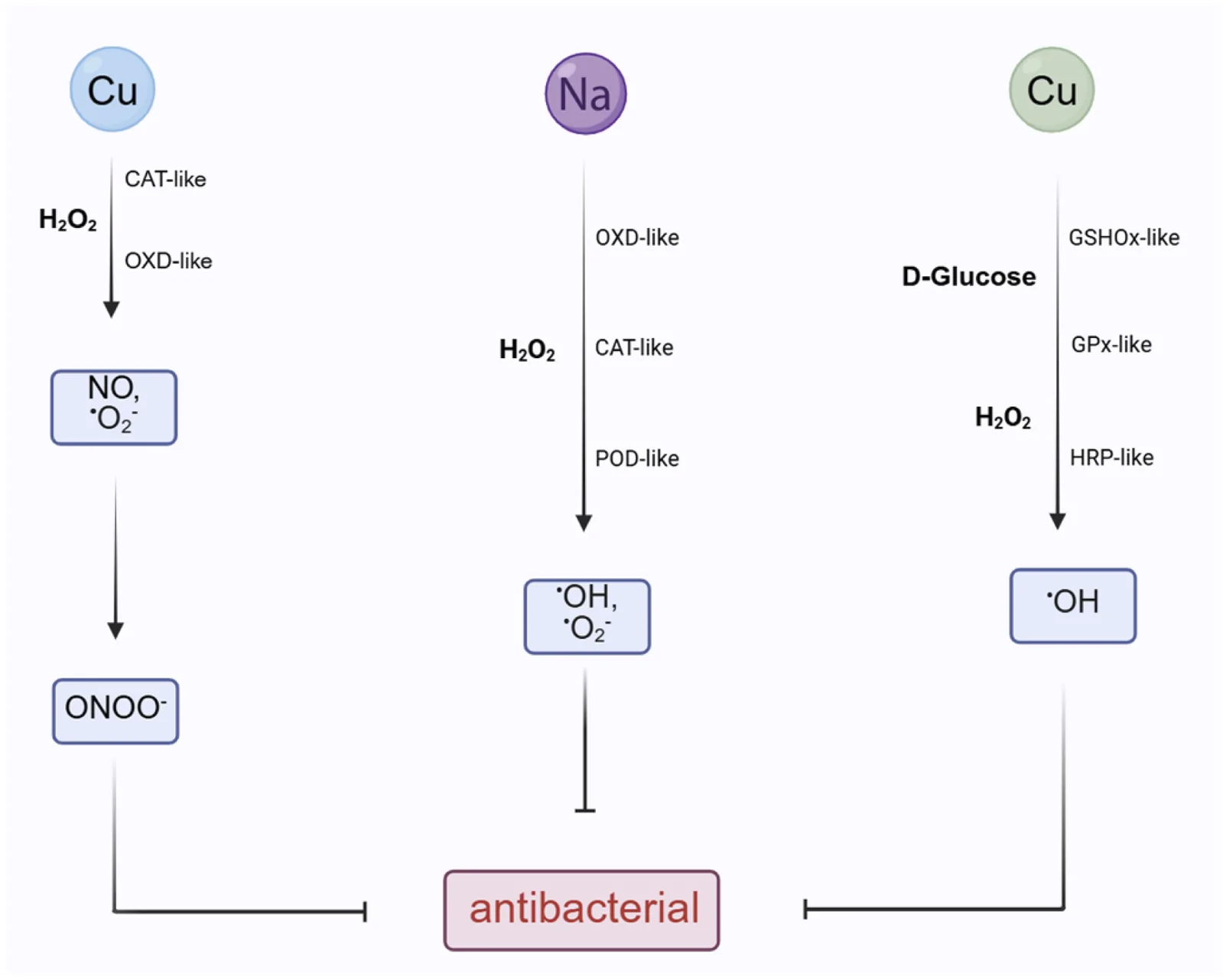
Schematic illustration of the antibacterial mechanisms of three representative SAN systems. Left: Cu-based SANs exhibit catalase (CAT)- and oxidase (OXD)-like activities, promoting the generation of NO and superoxide radicals (O_2_˙^−^), which react to form highly reactive peroxynitrite (ONOO^−^) for antibacterial activity. Middle: Na-based SANs exhibit OXD-, CAT-, and POD-like activities in the presence of H_2_O_2_, generating reactive oxygen species, including ·OH and O_2_·^−^ radicals, that contribute to bacterial elimination. Right: Cu-based SANs exhibit GSHOx-, GPx-, and HRP-like activities, promoting H_2_O_2_-mediated ·OH generation and disruption of bacterial redox homeostasis. The figure was created using https://BioRender.com.

**Table 4:** Comparison of representative SANs for antibacterial applications

SAN system	Active site structure	Core catalysis	Antibacterial performance	Working mechanism	Test model	Ref.
Ru/NiFe-LDH	O-coordinated Ru single atoms	PMS activation, ^1^O_2_-dominant	7-Log *E. coli* killed in 90 s (40 mg L^−1^ SANs + 5 mg L^−1^ PMS)	^1^O_2_ triggers lipid/protein damage; weak ·OH/SO_4_·^−^ byproducts	*E. coli*	223
HMMo/GOx@HA	Mo–N single-atom sites	POD-like	90% *in vitro* sterilization; 95.72% wound bacteria clearance	GOx produces H_2_O_2_; acidic microenvironment boosts ·OH generation	*S. aureus*	224
Cu-CNNDs	Cu–N_3_ coordination sites	POD-like	99% *E. coli* & 99.7% *S. aureus* killing, 6-log bacterial reduction	Optimized Cu–N_3_ coordination maximizes active site accessibility; H_2_O_2_ → ·OH	*E. coli* and *S. aureus*	225
Cu SAN-BDC	Cu–N_4_ single atoms	GOx-like + POD-like	<1.5% viable MRSA/*E. coli*, 95% wound repair	Biofilm-targeted glucose/GSH depletion + sustained ·OH production	*E. coli* and MRSA	226

The first pathway, non-radical oxidative damage, is exemplified by systems that generate singlet oxygen as the dominant bactericidal species. Xiang *et al.* demonstrated SAN-mediated Fenton-like catalysis using Ru/NiFe-LDH. Atomically dispersed Ru supported on layered double hydroxide (LDH) enabled targeted inactivation of *E. coli*.^223^ The Ru/NiFe-LDH structure facilitated electrostatic adsorption of *E. coli* while stabilizing Ru single atoms for localized ROS production. This increase in ROS production results in a corresponding increase in the rate of inactivation of *E. coli*. At very low doses (40 mg L^−1^ Ru/NiFe-LDH plus 5 mg L^−1^ peroxymonosulfate), this system achieved a 7-log reduction of *E. coli* within 90 seconds. It worked mainly through singlet oxygen (^1^O_2_)-mediated protein crosslinking and lipid peroxidation. Remarkably, metal leaching remained low, addressing a key limitation of conventional antimicrobials. The pH-dependent studies showed that the effect became weaker under acidic or alkaline conditions. This was caused by poor PMS adsorption and lower ROS generation. Neutral pH best improved the electrostatic co-localization of *E. coli* and oxidants. In a neutral environment, the positively charged material attracts both negatively charged *E. coli* and PMS. The ROS generated can act on *E. coli* immediately. This resulted in the most efficient inactivation under the studied pH conditions. Mechanistic analysis identified ^1^O_2_ as the dominant bactericidal species, with secondary contributions from sulfate (SO_4_·^−^) and ·OH, collectively disrupting bacterial membranes *via* protein aggregation and lipid oxidation. Concurrently, the catalyst further prevented secondary contamination by degrading lysed cellular debris, demonstrating dual bactericidal and environmental remediation capabilities. The Ru/NiFe-LDH system demonstrates that non-radical pathways, particularly ^1^O_2_-mediated damage, can achieve rapid and efficient bacterial inactivation while minimizing the reliance on ·OH generation.

The second pathway involves cascade catalysis that utilizes endogenous glucose to generate H_2_O_2_ and subsequently produce ·OH. Zhang *et al.* developed a hollow mesoporous molybdenum SAN (HMMo-SAzyme) demonstrating POD-like activity and mesoporous nanocarrier functionality.^224^ Loading GOx onto HMMo-SAzyme followed by hyaluronic acid (HA) encapsulation produced a glucose-responsive nanoreactor (HMMo/GOx@HA), effective against bacterial infections *in vivo*. At infection sites, hyaluronidase (HAase) degraded HA, enabling GOx to convert glucose into gluconic acid and H_2_O_2_. The resulting acidic environment enhanced HMMo-SAzyme's catalytic activity, converting H_2_O_2_ into ·OH for bacterial eradication. As shown in Fig. 15, this nanozyme caused minimal cytotoxicity *in vitro* and no appreciable systemic toxicity *in vivo*, while markedly inhibiting bacterial growth. The HMMo/GOx@HA system introduces glucose responsiveness as a triggering mechanism, enabling bacteria-activated therapy that only generates ·OH in the presence of infection-site glucose and hyaluronidase.

**Fig. 15:**
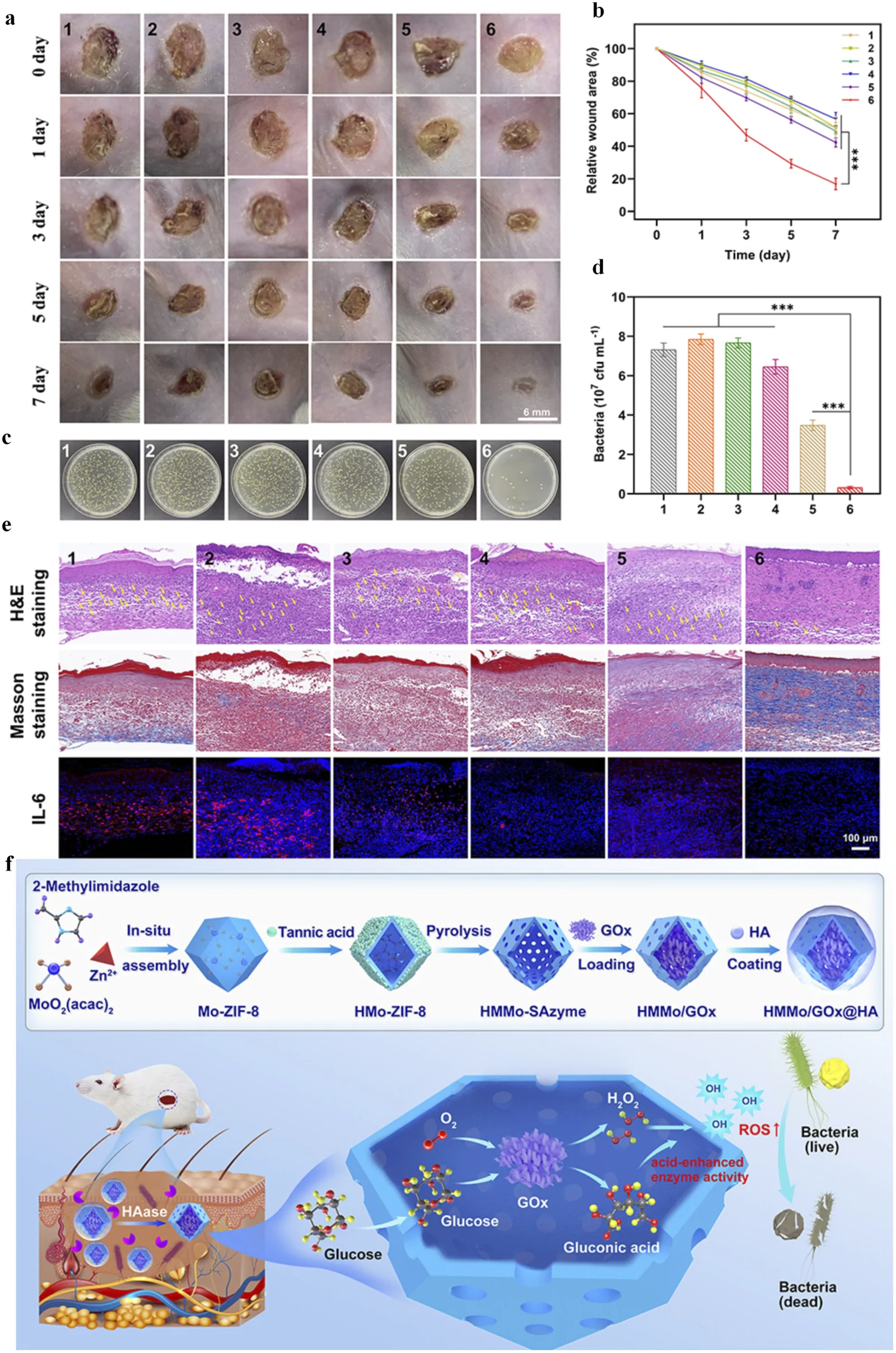
(a) Photographs of wounds of mice infected with *S. aureus* after receiving different treatments. (b) Relative wound area of mice. (c) Photographs of bacterial colonies formed by bacteria obtained from wound tissues. (d) Number of surviving bacteria in the infected wounds of mice with different treatments. (e) H&E staining, Masson staining, and immunofluorescence staining for IL-6 of wounds after 7 days of treatment. Yellow arrows indicate the infiltration of inflammatory cells. Different treatments in (a–e): (1) PBS, (2) glucose, (3) HMMo/GOx@HA, (4) glucose + HMMoSAzyme, (5) glucose + GOx, (6) glucose + HMMo/GOx@HA. (f) Schematic representation of the mechanism of action of HMMo/GOx@HA. Reproduced with permission.^224^ Copyright 2023, Dove Medical Press.

A third strategy integrates multiple reactive species, including both ROS and RNS, for synergistic antibacterial effects. Copper-based SANs exemplify multifunctional catalysis by exhibiting dual CAT- and OXD-like activities at the Cu single-atom sites, which convert H_2_O_2_ into O_2_ and ROS for synergistic bacterial eradication. Qiu *et al.* synthesized l-Arg@Cu-SAzymes, which consist of multifunctional Cu single-atom nanozymes loaded with l-arginine. Low levels of H_2_O_2_ were used as triggers.^152^ Capitalizing on the CAT- and OXD-like activities of the Cu sites to produce cytotoxic·O_2_^−^, and on the ability of l-Arg to react with H_2_O_2_ to produce NO, the concurrent generation of·O_2_^−^ and NO led to peroxynitrite (ONOO^−^) formation, amplifying RNS and ROS. These reactive species depolarized MRSA membranes, increasing permeability and inducing lethal ion imbalance while sparing host tissues. Simultaneously, the system stimulated fibroblast proliferation, collagen deposition, and angiogenesis, accelerating wound regeneration. Additionally, macrophage polarization to a reparative M2 phenotype was promoted, which accelerated wound closure and tissue remodeling. The l-Arg@Cu-SAzyme system achieves enhanced bactericidal efficacy through the cooperative action of multiple reactive species, where ONOO^−^ formation provides a distinct antimicrobial mechanism beyond ROS alone. In a related development, Dai and colleagues have highlighted that the exceptional POD-like activity of Cu SANs (Cu-CNNDs) can be attributed to the size effect of the optimized coordination structure.^225^ The porous carbon support maximized the accessibility of Cu–N_3_ active sites, thereby enhancing the capacity of H_2_O_2_ to generate ·OH. *In vitro*, these composites displayed potent bactericidal activity under the tested conditions. The Cu-CNND system further validates that copper-based SANs can achieve potent antibacterial activity through coordination environment optimization, with the Cu–N_3_ configuration enhancing accessibility of active sites.

Beyond compositional and structural optimization, spatiotemporal control of SAN synthesis offers an additional dimension for enhancing antibacterial performance. Huang *et al.* developed a spatiotemporal synthesis strategy for Cu SANs (Cu SAN-BDC), which bypasses traditional MOF pyrolysis constraints to achieve high atomic dispersion.^226^ This approach involves a spatiotemporally guided Cu single-atom bio-nano-enzyme (Cu SAN-BDC) strategy for precise anti-biofilm treatment. The Cu–N_4_ SAzyme was combined with GOx and ConA. GOx oxidized glucose to generate H_2_O_2_ and gluconic acid, while ConA aided biofilm localization; Cu sites mediated downstream peroxide/GSH reactions. This enables the product to localize the biofilm glycocalyx and catalyze the conversion of endogenous glucose into hydrogen peroxide and gluconic acid. This process results in the consumption of glucose and glutathione, while producing ·OH. The spatiotemporally guided bio-nanocatalyst demonstrated efficacy in the inhibition of *E*. *coli* and methicillin-resistant *Staphylococcus aureus* biofilms in both *in vitro* and *in vivo* settings. Additionally, it exhibited wound-healing promotion properties. The Cu SAN-BDC system introduces spatiotemporal synthesis as a design parameter, demonstrating that the synthesis route itself can be engineered to achieve high atomic dispersion and biofilm-localized activity, offering a complementary approach to conventional pyrolysis-based methods.

### 8.2 Cancer therapy

Conventional cancer therapies such as chemotherapy and radiotherapy eliminate tumor cells but indiscriminately harm healthy tissues. In contrast, TME-responsive nanocatalytic therapy offers a targeted alternative for cancer treatment. The therapeutic efficacy of SANs in oncology stems from their ability to exploit the unique chemical features of the tumor microenvironment, including elevated H_2_O_2_ levels, acidic pH, and glutathione overexpression, to achieve cytotoxicity preferentially in tumor cells, with markedly lower activity toward normal cells under tested conditions. SANs are widely utilized in tumor therapy due to their precise catalytic capabilities (Fig. 16). Within tumor cells, SANs drive Fenton-like reactions that convert H_2_O_2_ into ·OH, generating ROS to directly kill cancer cells and regulate antitumor immunity.^227^ The TME's elevated H_2_O_2_ levels amplify nanozyme-mediated chemodynamic therapy (CDT), while limited H_2_O_2_ in normal cells restricts off-target Fenton reactions, preserving healthy tissue. The first strategy involves direct CDT utilizing single-metal SANs with intrinsic TME responsiveness. Qiao *et al.* developed manganese-based SANs (Mn-N/C) by embedding Mn single atoms into ZIF-8 frameworks.^29^ Under acidic TME conditions, Mn-N/C showed stronger POD-like activity than MnO_2_ nanoparticles, producing higher ROS levels while maintaining Fenton-like functionality. Intratumoral injection of Mn-N/C (70 mg kg^−1^, 50 µL in saline) suppressed CT26 colon tumor growth, reduced tumor mass, and extended murine survival. Critically, Mn-N/C showed no detectable toxicity to normal intestinal epithelial cells (IEC), which supports its favorable *in vitro* biosafety profile. The Mn-N/C system demonstrates that manganese-based SANs can achieve enhanced POD-like activity compared to conventional MnO_2_ nanoparticles, with the acidic TME serving as an endogenous trigger for selective tumor killing. Similarly, Jin *et al.* prepared Mn-CDs SANs on Mn-N_2_-configured CD carriers.^228^ Mn-CDs SANs exhibit excellent dispersibility and biological safety. These nanostructures achieve deeper two-photon imaging (34 µm) and lysosome-specific targeting (Pearson's coefficient = 0.752) at the cellular level. Their Fenton-like reactions and POD-like activity (SA = 0.64 U mg^−1^) effectively kill tumor cells and contract tumor tissue *in vitro* and *in vivo* (Fig. 17). The Mn–CD system extends the functionality of Mn-based SANs by integrating two-photon imaging capability, enabling simultaneous therapeutic intervention and real-time tumor visualization.

**Fig. 16:**
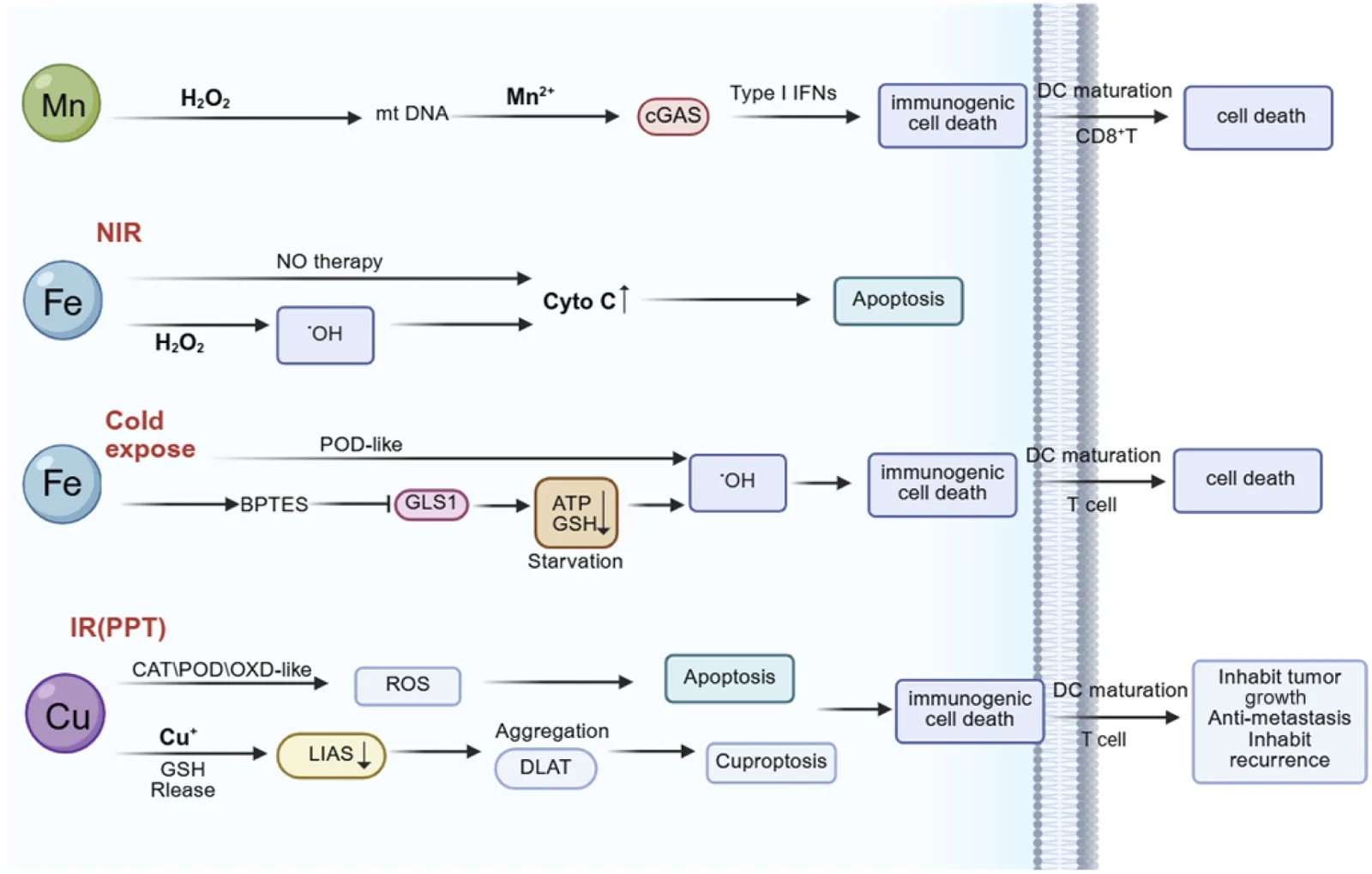
Schematic illustration of the principal mechanisms by which SANs induce tumour-cell death and antitumour immunity. Mn-based SANs promote mitochondrial DNA damage and Mn^2+^-mediated activation of the cGAS pathway, leading to type I interferon production, immunogenic cell death, dendritic-cell maturation, and CD8^+^ T-cell activation. Fe-based SANs under near-infrared (NIR) irradiation combine NO therapy with H_2_O_2_-mediated ·OH generation to promote cytochrome c release and apoptosis. Exosome-delivered Fe-based SANs exhibit POD-like activity and modulate glutamine metabolism, leading to ATP and glutathione (GSH) depletion, metabolic starvation, ·OH generation, and immunogenic cell death. Cu-based SANs under infrared photothermal therapy [IR(PTT)] exhibit CAT-, POD-, and OXD-like activities and release Cu^+^, promoting ROS-mediated apoptosis and cuproptosis through lipoyl synthase (LIAS) downregulation and dihydrolipoamide S-acetyltransferase (DLAT) aggregation. The resulting immunogenic cell death promotes dendritic-cell maturation and T-cell activation, contributing to tumour-growth inhibition, antimetastatic effects, and immune responses. The figure was created using https://BioRender.com.

**Fig. 17:**
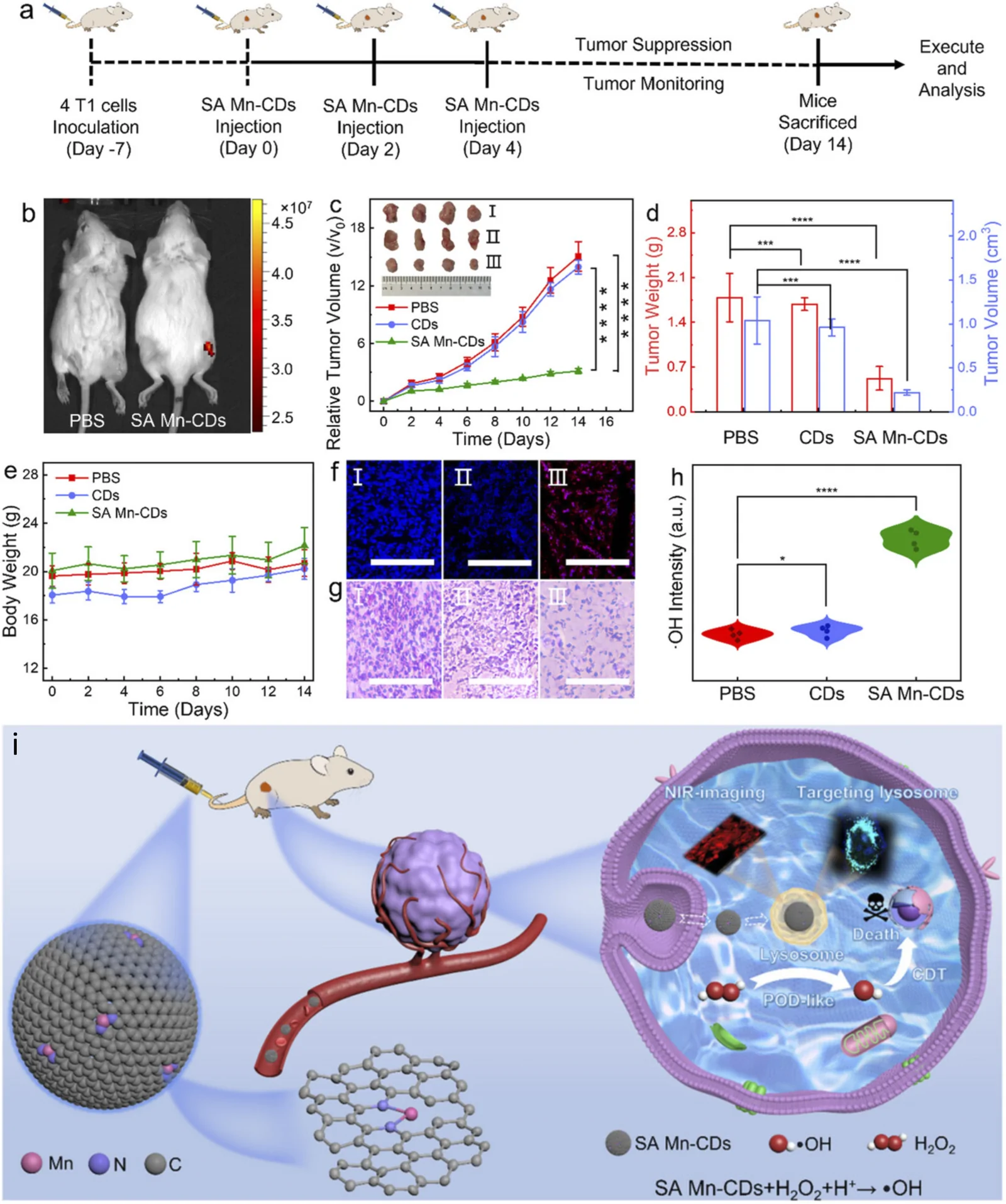
*In vivo* tumor catalytic therapy of SA Mn-CDs. (a) Schematic diagram of treatment schedule. (b) Fluorescence imaging of tumor-bearing mice intravenously inoculated with SA Mn-CDs under 680 nm excitation. (c) Tumor growth curves of mice under various treatments for a period of 14 days after receiving the first dose of administration. The inset (c) shows the tumors collected from mice treated under different conditions on day 14. (d) Average weights and volume of tumors collected from mice treated under different conditions on day 14. (e) Body weight changes of mice treated under different conditions within 14 days. (f) TUNEL confocal fluorescence images of dissected tumor tissue after 120 h treatment with PBS (I), CDs (II) SA Mn-CDs (III). (g) H&E-stained tumor sections from mice with different treatments. (I) PBS, (II) CDs, and (III) SA Mn-CDs. (h) ·OH intensity quantified from 8 random fields of view selected from each group. (i) Schematic representation of the mechanism of action of SA Mn-CDs. The scale bar is 100 µm. Reproduced with permission.^228^ Copyright 2025, Wiley-VCH GmbH.

Beyond direct CDT, metabolic modulation offers a complementary approach to enhance therapeutic outcomes. As the use of CDT and single-atom nanomedicines in tumor therapy becomes more prevalent, a novel therapeutic approach, cold exposure (CE) therapy in conjunction with nanomedicines, which results in the metabolic starvation of tumor cells, offers a new perspective on the potential of nanomedicine combination therapy modalities. Zhang *et al.* engineered a platelet membrane-coated system (PFB) co-delivering Fe-SANs and the glutaminase inhibitor BPTES to tumors.^138^ The substantial quantity of hydrogen peroxide present within tumor cells impairs the integrity of the PM, facilitating the release of BPTES and Fe-SAEs encapsulated within the PM to form a PFB. Concurrently, the enhanced POD-like activity of Fe-SAEs transforms H_2_O_2_ into ·OH, thereby inducing an elevation in oxidative stress. Subsequently, CE synergizes with BPTES to block glucose uptake, intensifying ROS cytotoxicity through dual metabolic starvation. The PFB system illustrates that SANs can be integrated with metabolic inhibitors and physical stimuli such as cold exposure, where oxidative stress and metabolic blockade cooperatively amplify tumor cell death.

A third paradigm integrates SANs with multiple therapeutic modalities including gas therapy, photothermal therapy (PTT), and copper-induced proteotoxicity to further enhance antitumor efficacy. Xie *et al.* demonstrated that synergistic SAN-catalyzed NO gas therapy against *in situ* oral squamous cell carcinoma (OSCC) could be achieved through the design of Fe SANs (SAF NCs) loaded with S-nitrosothiol (SNO) donors.^229^ Concurrently, PTT utilizing near-infrared laser irradiation can augment the Fenton-like catalytic activity of SAF NCs while inducing NO release through SNO thermolysis, thereby generating both toxic ·OH and anti-tumor therapeutic NO gases at the tumor site. This subsequently induces apoptotic cell death *via* the mitochondrial apoptotic pathway. The SAF NC system combines CDT, NO gas therapy, and PTT within a single platform, demonstrating that SANs can serve as multimodal therapeutic hubs where each modality complements the others. Wu *et al.* synthesized a sputtered Cu SAN (CuSACO) using *in situ* bombardment embedding.^217^ CuSACO exhibits a distinctive planar CuC_3_ coordination configuration, which not only achieves near-complete utilization of reactive atoms but also exhibits exceptional CAT, OXD, and POD-like activities. In acidic/GSH-rich TME, CuSACO generates cytotoxic ROS (*e.g.*, ·OH) to eradicate tumors. Furthermore, CuSACO downregulates lipoic acid synthase (LIAS), inhibits dihydrolipoyl transacetylase (DLAT) activity, and induces proteotoxic stress through copper–protein aggregation, thereby enhancing the efficacy of cancer therapy. Meanwhile, under NIR irradiation, CuSACO's photothermal effect further amplifies ROS production. In conclusion, the combination of enzyme-mimicking catalytic therapy with CuSACO, copper dysregulation and PTT has significantly enhanced the role of immunotherapy, potently activated ICD and elicited strong antitumor immune responses, leading to enhanced anticancer efficacy. The CuSACO system introduces a distinct mechanism by exploiting copper-induced proteotoxic stress alongside catalytic ROS generation, where the planar CuC_3_ coordination configuration enables both high atomic utilization and multi-enzyme activity. Niu *et al.* constructed a piezoelectric charge-assisted ultrasound-driven co-delivery system incorporating triphenylphosphonium (TPP) and methyl (*Z*)-4-(chloro(2-phenylhydrazono)methyl)benzoate (MMB), forming an Fe-based single-atom nanozyme (BFTM).^230^ This system activates apoptosis and promotes fumarate metabolism. Simultaneously, the positive and negative charges generated by barium titanate (BTO) modulate the electron density of Fe single atoms, enhancing their ROS generation efficiency. Furthermore, bioorthogonal consumption of intracellular fumarate augments pyroptosis, triggering robust immunogenic cell death in tumor cells and activating T-cell responses, thereby significantly improving antitumor immunotherapy efficacy. The BFTM system extends the multimodal paradigm by incorporating piezoelectric charge assistance and bioorthogonal chemistry, demonstrating that external physical stimuli and metabolic reprogramming can be integrated with SAN catalysis to achieve synergistic immunotherapeutic effects. These advances expand the scope for integrating SANs with multimodal therapeutic strategies (Table 5).

**Table 5:** Comparison of representative SANs for cancer therapeutic applications

SAN system	Active site	Core catalysis	Antitumor effect	Therapeutic route	Animal model	Ref.
Mn-N/C	Unsaturated Mn–N	POD-like	Tumor inhibition, CD8^+^ T elevation, low normal cell toxicity	Acidic TME ·OH chemodynamic therapy	CT26 colon tumor	29
Mn-CDs SANs	Mn–N_2_	Fenton + POD-like	Lysosome-targeted therapy, deep two-photon imaging	CDT with intracellular fluorescence tracing	4T1 subcutaneous tumor	228
PFB (Fe-SANs + BPTES)	Fe single-atom sites	POD-like	Dual metabolic starvation amplifies tumor oxidative stress	Cold exposure + BPTES metabolism block + CDT	4T1 primary & lung metastasis	138
SAF NCs (Fe SANs + SNO donors)	Fe single-atom sites	Fenton + POD-like	PTT synergistic tumor ablation	CDT + NO gas therapy + NIR photothermal therapy	Orthotopic oral cancer (OSCC)	229
CuSACO	Planar Cu–C_3_	CAT + OXD + POD-like	Trigger cuproptosis & ICD; PTT enhancement	Copper proteotoxicity + TME-responsive ROS + PTT	4T1 breast/GL261 glioma	217
BFTM (Fe SANs + BTO + MMB + TPP)	Piezoelectric Fe sites	GSHOx + POD-like	Pyroptosis, boosted anti-tumor T-cell immunity	Ultrasound-activated ferroptosis immunotherapy	4T1 bilateral/lung metastasis	230

### 8.3 Biosensing

Single-atom catalysts possess a multitude of advantageous properties, which have led to their extensive utilization in biosensing applications,^231^ in addition to their applications in pollutant degradation and energy conversion.^232^ The application of SANs in biosensing relies on their ability to catalyze signal-generating reactions with high specificity and efficiency, where the well-defined active sites enable reproducible and quantifiable responses to target analytes (Table 6). Zhou *et al.* developed a Ni single-atom sensor anchored on N-doped hollow carbon spheres (Ni SACs/NC) for cellular NO detection. Using HUVECs, the sensor showed no apparent cytotoxicity while maintaining nanomolar sensitivity, enabling real-time monitoring of drug- and stretch-induced NO release. This confirms its potential for electrochemical sensing applications.^233^ The Ni SACs/NC system demonstrates that single-atom catalysts can serve as electrochemical sensors for direct detection of biologically relevant small molecules, with the hollow carbon sphere structure facilitating mass transport and active site accessibility.

**Table 6:** Comparison of representative SANs for biosensing applications

SAN system	Metal/active site	Sensing mechanism	Detection target	LOD/performance	Application	Ref.
Ni SACs/NC	Ni single atomic sites	Electrochemical real-time monitoring	Intracellular nitric oxide (NO)	Nanomolar detection limit	Dynamic NO tracking in HUVEC cells	233
Fe-N/C SANs	Fe–N_*x*_ coordination sites	BPE-ECL dual signal amplification (POD + CAT mimicry)	Prostate-specific antigen (PSA)	0.62 pg mL^−1^	Clinical tumor immunoassay	234
RuLDH	Dispersed Ru single atom dopants	Optical spectral modulation	Tetracycline (TC) antibiotics	1.0 mg L^−1^	Environmental antibiotic detection	236
Co SANs (ZIF-8 derived)	Co single atoms	Luminol chemiluminescence *via* a Fenton-like reaction	5-Fluorouracil (5-FU)	Quantitative detection in human serum	Small-molecule competitive immunoassay	237
Au-SANs	Au single atoms (Au–N_4_)	Label-free aptamer electrochemistry/colorimetry (POD-like)	Thrombin; C-reactive protein (CRP)	550 pM (thrombin); 500 pg mL^−1^ (CRP)	Serum protein biomarker screening	238
Ru NC_Cl@µPAD	Out-of-plane Ru–Cl coordination	Near-neutral exclusive POD catalytic color reaction	Glucose, lactate, cholesterol, choline	Multiplex high recovery detection	Paper-based POC microfluidic diagnosis	235

For biomolecule detection, the catalytic signal amplification capability of SANs enables ultrasensitive immunoassays. To achieve the efficient determination of prostate-specific antigen (PSA), Chen *et al.* proposed an electrochemiluminescence (ECL) immunoassay based on bipolar electrodes (BPEs) that employs Fe single-atom nanozymes (Fe-N/C SANs) as a dual-signal amplifier.^234^ By leveraging the POD-like and CAT-like capabilities, Fe-N/C SANs situated at the cathode can catalyze the decomposition of H_2_O_2_ due to the presence of Fe–N ligand sites. Simultaneously, Fe-N/C catalyzed 4-CN/H_2_O_2_ precipitation, suppressing electron transfer at the anode. This generated an inverse correlation between PSA concentration and ECL signal intensity. The Fe-N/C system achieved a PSA detection limit of 0.62 pg mL^−1^, meeting clinical diagnostic requirements, and illustrates how SANs can amplify detection signals through dual POD-like and CAT-like pathways to create a signal inversely proportional to target concentration.

Antibiotic monitoring has become a critical demand in modern healthcare. To enable rapid and sensitive antibiotic detection for biomonitoring under environmentally challenging conditions, Zheng *et al.* developed a Ru single-atom catalyst (RuLDH) with notable stability and optical sensing activity.^236^ The Ru single-atom doping introduces charge-trapping sites that enhance the material's optical properties, achieving a detection limit of 1.0 mg L^−1^ for TC. This work provides a novel perspective on the design of single-atom catalysts for biosensing applications. The RuLDH system extends the application of SANs to optical sensing, where single-atom doping creates charge-trapping sites that modulate optical properties in response to antibiotic binding.

Chemiluminescence-based detection represents another important sensing modality for SANs. To achieve an efficient determination of 5-fluorouracil (5-FU) in human serum, Li *et al.* established Co SANs using ZIF-8 MOFs.^237^ Co SANs exhibit Fenton-like activity, catalyzing the decomposition of H_2_O_2_ to generate superoxide radical anions. This amplifies the chemiluminescence signal of the luminol–H_2_O_2_ system and provides stable signal output over the course of the immunoassay. The immunosensing platform employed carboxyl-modified resin beads to immobilize 5-FU-conjugated antigens. In this competitive immunoassay, free 5-FU standards compete with immobilized 5-FU antigens for limited antibody binding sites on the carboxylated microspheres. This competition depends on 5-FU concentration, generating corresponding chemiluminescence signals that enable quantitative detection in human serum and provide a sensitive analytical approach for 5-FU and analogous small molecules. The Co SAN system demonstrates that Fenton-like activity can be harnessed for chemiluminescence amplification, enabling quantitative detection of 5-FU in human serum. Aptamer-based sensors offer a label-free approach for protein detection using SANs. In the context of human serum and other substances present in plasma, Ali *et al.* have developed Au SANs for the purpose of detecting thrombin and C-reactive protein (CRP).^238^ In the presence of AuN_4_, the POD-like Au SANs demonstrated activity, adsorbing H_2_O_2_ and converting it to ·OH and ^1^O_2_. Ultimately, an aptamer-based sensor was devised for the detection of thrombin and CRP. This was achieved through the label-free integration of the sulfhydryl-terminal aptamer onto the surface of individual Au atoms (aptamer/Au-SANs), resulting in satisfactory accuracy and precision with detection limits of 550 pM and 500 pg mL^−1^, respectively. The aptamer/Au-SANs system introduces a label-free detection strategy where aptamer recognition is directly coupled to the POD-like activity of Au single atoms, eliminating the need for secondary antibody labeling. Beyond single-analyte detection, SANs can be engineered for multiplexed biomarker quantification through rational ligand design. Park *et al.* constructed a Ru NC_Cl complex with an out-of-plane coordinated active center.^235^ Cl ligands inhibit additional H_2_O_2_ adsorption, suppressing CAT-like activity. These atomically dispersed 3D active sites display exclusive POD-like functionality under near-neutral conditions. In the CAT-like reaction of Ru NC_Cl, the rate-determining step involves introducing an additional H_2_O_2_ molecule (·O → ·O + ·HOOH), with an energy barrier of 1.62 eV. In contrast, the POD-like reaction exhibits a dramatically lower barrier of only 0.23 eV for the rate-determining step of water release. Rational ligand design also maximizes Ru utilization. It allows accurate quantification of glucose, lactate, cholesterol, and choline in human serum with recoveries and reproducibility demonstrated in spiked human serum samples. This makes the complex a reliable platform for point-of-care (POC) detection (Fig. 18). The Ru NC_Cl system demonstrates that ligand engineering can suppress unwanted enzymatic pathways while enhancing the desired POD-like activity, enabling multiplexed detection of multiple biomarkers on a single paper-based microfluidic device. The low energy barrier of 0.23 eV for the rate-determining step underpins the high catalytic efficiency at near-neutral pH, which is essential for physiological samples.

**Fig. 18:**
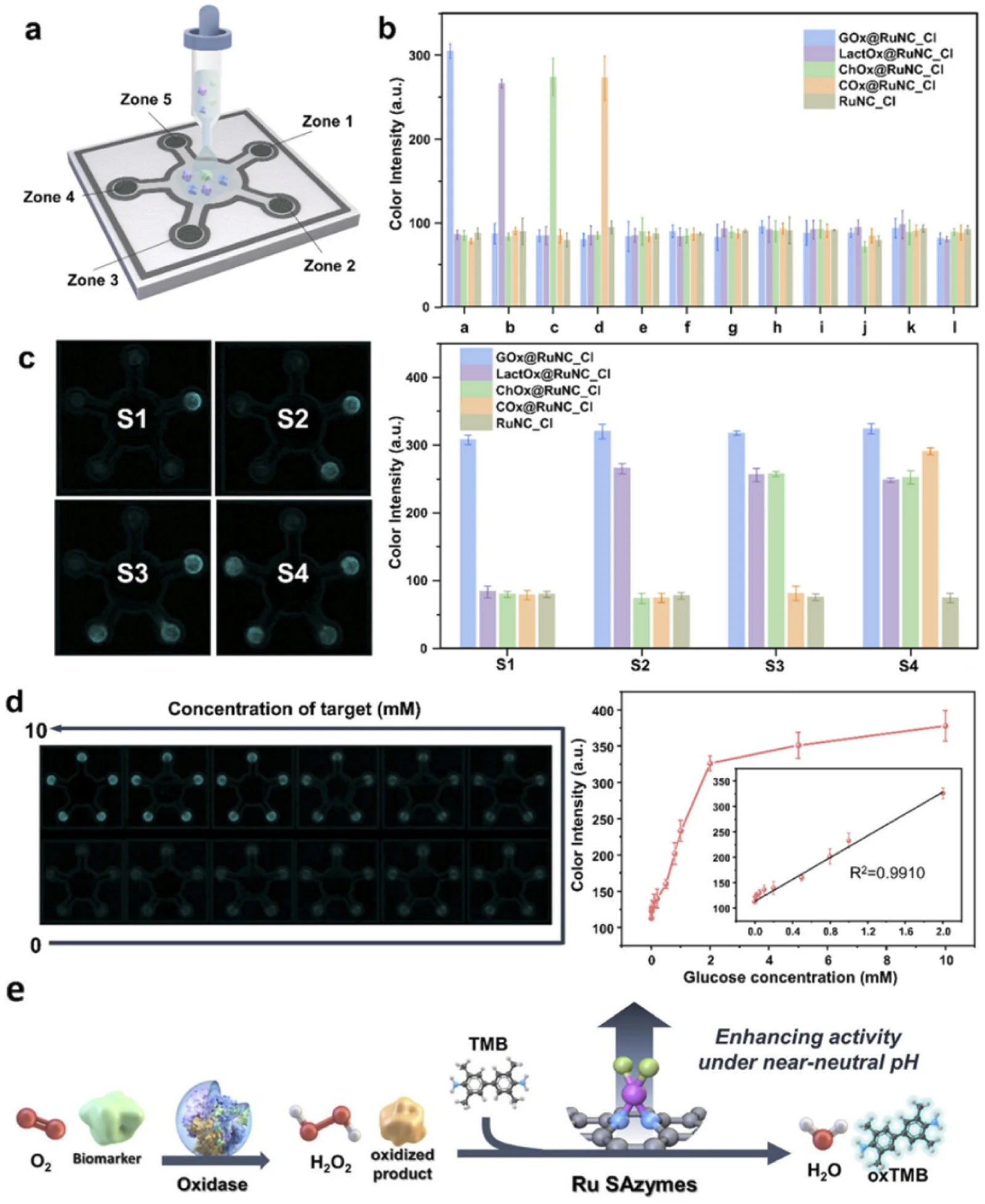
Biomarker quantification with paper-based microfluidic sensors using RuNC_Cl. (a) Illustration of the RuNC_Cl-based paper microfluidic devices (RuNC_Cl@µPAD) for multiple target detection in which GOx@RuNC_Cl (zone 1), LactOx@RuNC_Cl (zone 2), ChOx@RuNC_Cl (zone 3), COx@RuNC_Cl (zone 4), and RuNC_Cl (zone 5). (b) Specificity of the RuNC_Cl@µPAD for the detection of single analyte ((a): glucose, (b): lactate, (c): cholesterol, (d): choline, (e): galactose, (f): BSA, (g): cysteine, (h): ascorbic acid, (i): uric acid, (j): Na^+^, (k): Ca^2+^, (l): buffer). (c) Specificity of the device for the detection of multiple biomarkers: solution 1 (glucose), solution 2 (glucose + lactate), solution 3 (glucose + lactate + cholesterol), and solution 4 (glucose + lactate + cholesterol + choline). (d) The dose–response curve and photographs of the glucose detection using the GOx@RuNC_Cl-based paper device. (e) Mechanism of action of respective enzyme@RuNC_Cl. Reproduced with permission.^235^ Copyright 2025, Wiley-VCH GmbH.

### 8.4 Oxidative-stress protection

Oxygen drives essential redox processes in biological systems, including the generation of ROS. Under physiological conditions, moderate ROS levels participate in fundamental cellular functions by regulating the metabolism of lipids, proteins, and nucleic acids, contributing to biomolecule synthesis, signal transduction, and programmed cell death. However, redox homeostasis is tightly controlled; excessive ROS accumulation disrupts this balance and inflicts oxidative damage on cellular structures and surrounding tissues. Antioxidants, particularly SOD and CAT, therefore serve as critical guardians against oxidative stress. Yet, natural enzymes suffer from limited stability under physiological conditions, hampering their therapeutic application. This limitation has spurred the search for efficient artificial enzyme mimics. Owing to their outstanding enzyme-mimetic activity and superior stability, SANs have emerged as highly promising candidates for protecting cells from oxidative insult.^239,240^ The cytoprotective function of SANs is achieved through their ability to scavenge multiple ROS species *via* SOD-like, CAT-like, and GPx-like activities, with the specific combination of enzyme-mimetic functions tailored to the pathological context of each disease (Table 7).

**Table 7:** Comparison of representative SANs for oxidative-stress cytoprotection applications

SAN platform	Active site structure	Catalytic functions	Core protective effects	Working mechanism	Disease model	Ref.
Zn/C-dots	Isolated Zn single atoms on carbon dots	POD + SOD-like	Efficient˙OH/O_2_^−^ scavenging, reduced inflammatory cytokines	Mitochondrial accumulation to eliminate excess ROS	Diabetic wound healing	241
Pt SA/C_3_N_4_	Pt single atomic sites on carbon nitride	SOD + CAT-like	Alleviate oxidative phosphorylation disorder, slow OA progression	NIR light boosts antioxidant activity to remodel cell redox balance	Osteoarthritis (OA) progression model	151
Pt/CeO_2_	Pt single atoms	CAT + SOD-like	Lower brain inflammatory factors, protect dopaminergic neurons	Target damaged mitochondria to trigger mitophagy, cut ROS source	Parkinson's disease (PD)	242
Ru SANs	Ru single atoms	SOD + CAT + GPx-like	Suppress lipid peroxidation, relieve ferroptosis risk	Multifunctional ROS clearance pathway	Intrauterine adhesions (IUAs)	243
Fe/Mn DAN	Bimetallic Fe–N & Mn–N dual sites	SOD + CAT-like	Reduce ocular ROS, inhibit NLRP3 inflammasome	Complementary redox cycling to relieve ocular inflammation	Dry eye disease	244

In the context of diabetic wounds with elevated oxidative stress, bacterial infections, and chronic inflammation during the healing process, Dai *et al.* developed and synthesized an ultrasmall carbon dot (C-dots) formulation loaded with zinc SANs (Zn/C-dots).^241^ These SANs are capable of effectively entering cells and accumulating in mitochondria. They exhibit excellent POD-like and SOD-like activities, effectively protecting cells from oxidative stress damage and limiting the release of pro-inflammatory cytokines by efficiently scavenging ·OH and O_2_·^−^ radicals. This ultimately results in the removal of excess ROS, thereby reducing inflammation and playing an anti-inflammatory role. This approach accelerated diabetic wound healing in murine models. The Zn/C-dot system demonstrates that ultrasmall SANs can achieve mitochondrial accumulation and dual POD/SOD activity, addressing both oxidative stress and inflammation in diabetic wound healing.

Xiang *et al.* reported the synthesis of Pt SANs that, in turn, were shown to slow the progression of OA by mimicking the activities of SOD and CAT through the modulation of the oxidative phosphorylation pathway.^151^ The progression of OA has been reported to be due to the overproduction of ROS and RNS, as well as aberrant ATP energy metabolism, which is associated with oxidative phosphorylation pathways in mitochondria. Furthermore, the research team discovered that Pt SA/C_3_N_4_ displays remarkable photothermal conversion capabilities when exposed to NIR (808 nm) laser radiation. Additionally, initial NIR exposure (5 minutes) induced negligible additional ROS production. Concurrently, the exceptional SOD-like and CAT-like capabilities of Pt SA/C_3_N_4_, particularly when aided by NIR light irradiation, facilitate the elimination of ·NO, O_2_·^−^, and ·OH, thereby remodeling the cellular microenvironment and promoting the alleviation of OA progression. The Pt SA/C_3_N_4_ system extends the antioxidant strategy by integrating SOD/CAT activities with photothermal modulation, where NIR irradiation enhances enzyme-mimetic performance while simultaneously addressing aberrant mitochondrial energy metabolism in osteoarthritis. Li *et al.* synthesized Pt/CeO_2_ SANs that suppress neuroinflammation through dual mechanisms. The Pt SANs mimic multiple enzymatic activities, including CAT and SOD, to efficiently scavenge intra- and extracellular ROS, rapidly alleviating oxidative stress. Simultaneously, Pt SANs target dysfunctional mitochondria *via* electrostatic adsorption. By depleting local H^+^ ions, they disrupt both the α-glycerophosphate and malate–aspartate shuttle pathways, leading to mitochondrial membrane depolarization and triggering mitophagy. This process eliminates ROS production at its source, preventing recurrent inflammation. In a Parkinson's disease (PD) mouse model, Pt SAN treatment significantly reduced brain levels of proinflammatory cytokines (IL-1β, IL-6, TNF-α), restoring them to near-normal ranges. The therapy also reduced dopaminergic neuron damage and neuronal apoptosis, resulting in marked improvement in motor and non-motor symptoms. These outcomes confirm the efficacy of Pt SANs in mitigating neuroinflammation and preserving neuronal integrity.^242^ The Pt/CeO_2_ system introduces a distinct mechanism beyond ROS scavenging: mitochondrial targeting and mitophagy induction eliminate ROS production at its source, providing a more durable therapeutic effect compared to strategies that only neutralize existing ROS.

Oxidative stress interacts synergistically with other mechanisms, frequently precipitating the onset and progression of disease. For example, Liang and colleagues demonstrated that oxidative stress and ferroptosis are pivotal in the progression of intrauterine adhesions (IUAs). In contrast, Ru SANs, by virtue of their excellent SOD-like, CAT-like, and GPx-like activities, can scavenge ROS, including H_2_O_2_, ·OH, and O_2_·^−^, and can also utilize the antioxidant properties of Ru SANs to target ferroptosis, thus providing an effective therapeutic approach for IUA.^243^ The Ru SAN system adds GPx-like activity to the antioxidant repertoire, enabling the simultaneous targeting of ferroptosis, a regulated cell death pathway driven by lipid peroxidation, which is particularly relevant for diseases where iron-dependent oxidative damage is implicated. In addition to conventional SANs, dual SANs have the potential to play a significant role in protecting cells from oxidative stress. Chu *et al.* developed an antioxidant DAN by embedding Fe and Mn bimetallic single-atom sites into an N-doped carbon material, and this novel coordination strategy has demonstrated enhanced efficacy in preventing oxidative stress and anti-inflammatory responses in cells.^244^ The bimetallic synergy between Fe and Mn atoms effectively neutralizes the excessive ROS in the body environment, thus reducing oxidative stress and direct oxidative damage. This results in excellent biological activities in scavenging excessive ROS on the ocular surface, inhibiting the activation of NLRP3 inflammasome, decreasing the expression of pro-inflammatory cytokines, and inhibiting apoptosis. These effects contribute to the excellent efficacy of this treatment for dry eye disease. The Fe/Mn DAN system demonstrates that bimetallic synergy can outperform single-metal SANs in ROS scavenging and anti-inflammatory efficacy, with the Fe–Mn coordination providing complementary redox activities that are particularly effective for ocular surface inflammatory conditions such as dry eye disease.

## 9. Conclusions and outlook

Recent advances have established SANs as a promising platform in antimicrobial and anti-inflammatory therapies, cancer theranostics, and biosensing, owing to their cost-effectiveness, tunable synthesis, and high atomic utilization efficiency. Compared with conventional nanoparticle catalysts, SANs may offer enhanced catalytic activity, potentially reduced metal leaching, and favorable stability profiles under controlled conditions, yet nanoparticles retain practical merits including scalable production, morphological versatility, and design flexibility. Despite this progress, fundamental gaps remain. The precise link between the atomic-scale architecture of SANs and their catalytic function, particularly relative to atom clusters, has not been fully deciphered. Addressing this gap requires a systematic framework that connects structural features including coordination number, heteroatom doping, spatial configuration, and metal nuclearity to specific catalytic outcomes and biological functions.

Based on the structure–activity relationships discussed throughout this Review, several emerging design paradigms can be identified to guide future development.

(1) From single atoms to functional ensembles: while isolated metal atoms maximize atomic efficiency, reactions requiring multi-step catalysis or cooperative substrate activation, such as cascade antioxidant processes, often benefit from dual-atom or few-atom cluster configurations. The optimal nuclearity is reaction-dependent, necessitating a shift from a blanket “smaller is better” assumption to a functionally optimal design philosophy.

(2) Coordination environment as a tunable handle: beyond the conventional M–N_4_ motif, the coordination number, heteroatom doping, and axial ligation offer rich opportunities for electronic structure modulation. For instance, boron doping has been shown to enhance the three-dimensional accessibility of active sites, while phosphorus coordination at the remote position of Fe–N–C centers can induce lipid oxidase-like activity even at cold-chain temperatures. These findings underscore the importance of precision coordination engineering for tailoring catalytic selectivity.

(3) Spatial configuration matters: the precise positioning of active sites, whether interlayer, intraplanar, or at the metal–support interface, critically influences electron transfer pathways and substrate accessibility. This underexplored dimension offers new avenues for activity enhancement beyond traditional compositional optimization.

(4) Non-metal alternatives may offer opportunities to address biosafety concerns: the potential toxicity and potential accumulation of transition metals underscore the importance of developing non-metal SANs, including Se, B, and P, that combine catalytic activity with superior biocompatibility. Recent demonstration of Se single-atom nanozymes with NADPH oxidase-mimetic activity and low systemic toxicity under the evaluated conditions illustrates the potential of this direction, although significant synthetic and long-term safety challenges remain.

Despite remarkable preclinical progress, translating SANs from bench to bedside faces several hurdles that demand concerted efforts.

(1) Scalable synthesis with batch-to-batch reproducibility: current production methods, particularly pyrolysis and ALD, are inherently limited in throughput and consistency. The development of scalable, green synthetic routes that maintain atomic dispersion at loadings relevant for biomedical applications while ensuring batch-to-batch reproducibility represents a critical priority for industrial and clinical translation.

(2) *In vivo* fate and long-term safety: the biodistribution, metabolism, and clearance pathways of SANs remain poorly understood. While existing studies indicate favorable biocompatibility, systematic long-term toxicological evaluations in large animal models are urgently needed to assess chronic exposure risks, particularly for metal-based systems.

(3) Real-time catalytic monitoring *in vivo:* the absence of tools for visualizing and quantifying SAN-catalyzed reactions in living systems impedes mechanistic understanding and therapeutic optimization. Developing activatable probes and *operando* spectroscopic techniques for *in vivo* monitoring would be transformative for both fundamental research and clinical translation.

(4) Regulatory pathway uncertainty: given their classification as nanomedicines rather than small-molecule drugs, SANs face unclear regulatory pathways. Establishing standardized characterization protocols, engaging regulatory agencies early in development, and prioritizing locally administered formulations such as wound dressings, implant coatings, and local injections for early clinical entry may accelerate translation.

(5) Expanding therapeutic horizons: while antimicrobial and anticancer applications dominate current research, recent studies have demonstrated SANs' potential in treating diabetic wounds, Parkinson's disease, and Alzheimer's disease. These underexplored areas, along with diagnostics and tissue regeneration, offer exciting opportunities for future investigation.

In summary, SANs stand at a pivotal juncture where mechanistic understanding is rapidly converging with engineering innovation. The transition from empirical discovery to rational design, guided by the structure–activity frameworks articulated in this review, will define the next phase of SANs development. Addressing the translational barriers outlined above will require interdisciplinary collaboration spanning chemistry, materials science, biology, and clinical medicine.

Critical evidence gaps remain, particularly regarding long-term toxicological data in large animal models, standardized characterization and quality control protocols, and the translation of *in vitro* catalytic activity to complex biological environments.

As discussed above, locally administered formulations offer the most clinically accessible near-term entry points, providing valuable proof-of-concept data while minimizing systemic exposure.

In the longer term, systemically administered SANs hold potential for deep-seated tumors, metastatic disease, and systemic inflammatory conditions, provided that the translational barriers outlined earlier are addressed through interdisciplinary collaboration.

If these prerequisites are met, SANs may eventually join the clinical armamentarium. The path from proof-of-concept to patient benefit will be incremental, shaped by iterative feedback between fundamental discovery, engineering innovation, and clinical experience. The journey is promising, but it will require sustained effort, realistic expectations, and rigorous evidence at every stage.

## Author contributions

All authors approved the final version of the manuscript. Jinpei Cui: methodology, software, data curation, investigation, validation, formal analysis, writing – original draft, writing – review & editing. Xiaomin Xia: methodology, software, data curation, investigation, validation, formal analysis, writing – review & editing, writing – original draft. Xingyun Li: conceptualization, visualization, project administration, funding acquisition, resources, supervision, writing – review & editing. Dan Luo, Kaiqing Song and Sa Sun: methodology, data curation, investigation, formal analysis. Jie Liu: conceptualization, project administration, supervision, writing – review & editing, visualization. Shubiao Xia: conceptualization, visualization, project administration, supervision, resources, writing – review & editing. Mostafa Kamal Masud: conceptualization, validation, visualization, writing – review & editing, funding acquisition, resources, supervision. Yusuke Yamauchi: conceptualization, funding acquisition, writing – review & editing, visualization, project administration, resources, supervision. Xue Li: conceptualization, resources, project administration, writing – review & editing, funding acquisition, supervision, visualization.

## Conflicts of interest

There are no conflicts to declare.

## Data Availability

No new data were generated or analysed in this study.
